# Operating characteristics of agreement metrics in AI-based scoring: a Monte Carlo simulation

**DOI:** 10.3389/fpsyg.2026.1705653

**Published:** 2026-02-19

**Authors:** Alperen Yandi

**Affiliations:** 1 Department of Educational Sciences, Faculty of Education, Bolu Abant Izzet Baysal University, Bolu, Türkiye; 2 Department of Educational Measurement and Evaluation, Faculty of Education, Bolu Abant Izzet Baysal University, Bolu, Türkiye

**Keywords:** artificial intelligence, scoring reliability, Monte Carlo simulation, agreement metrics, measurement and evaluation in education and psychology

## Abstract

**Introduction:**

This study analyzed the threshold-exceedance performance of human and AI scoring agreement measures for scoring of open-ended items. Monte Carlo simulation was performed to represent the different types of errors encountered in automatic scoring determined by using studies in the literature. Accordingly, an examination was conducted of the agreement between human and artificial intelligence ratings under differing error conditions. The objective of the research was to ascertain the statistical power levels achieved by disparate agreement metrics under varying conditions. The objective of this research was to ascertain which metric would prove to be the most effective method, and under which conditions.

**Methods:**

Data with conditions including systematic additive bias, variance inflation, midpoint compression, class imbalance, and subgroup-related offsets. Human scores served as a reference to assess agreement. Agreement levels were evaluated with ICC(A,1), Krippendorff’s α (ranked), quadratic weighted kappa (QWK), and Bland-Altman along with tolerance-based agreement metrics. Threshold-exceedance performance was defined as the proportion with which each metric surpassed conventional adequacy standards. Analyses were also conducted on real data to validate the analyses conducted in the second part of the study. In this part, written texts were scored by six different students using three teachers and two large language models.

**Results:**

ICC(A,1) shown higher threshold-exceedance performance for low and moderate variance inflation. QWK was observed to reach a moderate level of robustness. Krippendorff’s a showed consistent performance, especially in conditions where the distributions were unbalanced or variance inflated. Tolerance-based fit demonstrated numerical closeness between human and AI scores. The findings showed patterns consistent with simulated impairments.

**Discussion:**

All findings indicate that fit indices vary systematically across different structural mechanisms and sampling conditions. The results suggest that different conditions can affect the interpretability of automated scores. Accordingly, the need for multi-metric assessment frameworks when assessing human-AI score fit is highlighted.

## 1 Introduction

Open-ended items offer students opportunities to use reasoning skills, demonstrate higher-order thinking skills, and generate answers through analysis and explanation (Brookhart, 2010; Freedman, 1981; Badger and Thomas, 1991; Karakaya, 2022; Yiğiter and Boduroğlu, 2025). Despite these opportunities, open-ended items have significant constraints for large-scale assessments. The scoring process for these items requires considerable time, manpower, and raters with scoring experience. Moreover, even if these requirements are fulfillmented, the raters’ objectivity may adversely affect the reliability and validity of the outcomes and interpretations (Baykul and Turgut, 2012; Güler, 2014; Hogan and Murphy, 2007; Karadağ et al., 2020). These limitations have led to an increase in studies on the use of artificial intelligence (AI) and natural language processing (NLP) technologies in the scoring process of these items. Research on automated scoring using these tools demonstrates that these technologies can achieve human-like accuracy across a variety of item types and language domains (Gierl et al., 2014; Ramesh and Sanampudi, 2022). Such technologies and models are powerful at detecting semantic patterns and producing scores consistent with human ratings (LaVoie et al., 2019; Zhu et al., 2022). However, evidence of basic reliability and validity may be insufficient to make accurate decisions and assess fairness based on automated scoring (Ercikan and McCaffrey, 2022; Debelak and Ziegler, 2025; Yiğiter and Boduroğlu, 2025). The concept of fairness is intrinsically linked to the consistency of score interpretations among subgroups (Camilli, 2006; American Educational Research Association [AERA] et al., 2014). The ambiguity over the error mechanisms of these technologies, the ambiguities their algorithms, and the potential for systematic biases sub-groups cast doubt on automated scoring. The studies indicates that automated scoring systems can exhibit variations in error patterns, fairness, and measurement equivalence based on demographic or contextual factors (Beiting-Parrish and Whitmer, 2023; Blodgett et al., 2020; Ferrara and Qunbar, 2022; Hahn et al., 2021; Mehrabi et al., 2021). The other researches involving students from different profiles within different subgroups indicate that interpretability and fairness are key factors affecting the automated scoring process (Korteling et al., 2021; Livingston et al., 2025; Nelson et al., 2025). AI-based scoring systems can have different measurement errors and biased feedback and unfair outcomes (Johnson and McCaffrey, 2023; Matta et al., 2023; Schaller et al., 2024). Research on algorithmic fairness suggests that AI systems can replicate or amplify existing inequalities in their training data. Therefore, it is important to assess the agreement between human and AI scoring systems and to determine whether interpretations of these similar to those derived from human raters.

Different mathematically based agreement metrics are used to assess agreement between humans and AI. Correlations such as Pearson and Spearman provide descriptive findings but do not reflect directional scoring inconsistencies (Giavarina, 2015). Cohen’s kappa coefficient adjusts for chance agreement. However, it is sensitive to prevalence and marginal imbalances and can yield low values even when percent agreement is high (Bexkens et al., 2018; Cicchetti and Feinstein, 1990). Krippendorff’s α coefficient is recommended for different measurement types and missing value conditions (Hayes and Krippendorff, 2007). Intraclass correlation coefficients (ICC), especially the absolute agreement form ICC(A,1), provide a general framework for continuous scores and multiple rater settings (Koo and Li, 2016). Other indices, such as Gwet’s AC1 and square weighted kappa (QWK), are also frequently used in multi-category scoring contexts (Yiğiter and Boduroğlu, 2025).

The existing literature presents limited results comparing the performance of fit metrics across different scoring conditions. Bland and Altman (2010) reported that high linear correlation can coexist with poor fit. Bıkmaz Bilgen and Doğan (2017) reported how the number of raters and rubric structure affect fit between assessment mechanisms. Zhai et al. (2021) identified construct-irrelevant variance (CIV) as the primary source of mismatch. These findings indicate that different conditions, such as additive bias, variance inflation, compression, and prevalence effects, can have different effects on the performance of fit indices. Therefore, a multifactorial evaluation of these metrics under controlled conditions is deemed necessary.

Threshold-exceedance performance is that proportion of exceedance the traditional thresholds used as criteria for metrics. Threshold-based performances can exhibit considerable variability in their sensitivity to sample size, score distribution, and structural distortions, in addition to the conditions mentioned above. Paradoxes such as the correlation of high percentage agreement with low kappa coefficients (Feinstein and Cicchetti, 1990) further complicate the use of these metrics. Monte Carlo simulations provide a model for resolving these complexities by allowing the manipulation of various conditions (Ercikan and McCaffrey, 2022).

The varying performances of metrics under different conditions highlight the inadequacy of assessing the scoring process with descriptive statistics. For all these reasons, examining human and AI agreements under different conditions and integrating tools sensitive to different conditions, such as Bland-Altman plots, have become a central topic of current research (Williamson et al., 2012; Johnson et al., 2022). This study incorporates potentially relevant conditions into the simulation design to examine how metrics perform under different conditions.

### 1.1 Purpose of study

This study aims to assess the threshold exceedance performance of agreement metrics for human-AI score concordance under 216 manipulated conditions. Specifically, the study evaluates the performance of ICC(A,1), Krippendorff’s α, QWK, and additional agreement metrics under conditions of (a) additivity bias, (b) variance inflation, (c) midpoint compression, (d) class imbalance, and (e) group-based offsets. Using a Monte Carlo approach is designed for these conditions, it aims to provide comprehensive guidance for interpreting agreement metrics in automated scoring.

### 1.2 Research questions

1. Which metrics demonstrate superior threshold-exceedance performance in human-AI score comparisons under additive bias?
2. Which metrics demonstrate higher threshold-exceedance performance in human-AI score comparisons under variance inflation?
3. Which metrics demonstrate superior threshold-exceedance performance in human-AI score comparisons under situations of midpoint compression and class imbalance?
4. What metrics identify subgroup differences under group-based offsets (fairness) while ensuring performance exceeds the acceptability level threshold?

The remainder of the article proceeds as follows:

The methodology section delineates the data generation procedures, simulation parameters, metric and model equations, and statistical analysis methods. The results section summarizes descriptive patterns, Type I error behavior, threshold exceedance outcomes, and logistic regression findings. The real-data validation section evaluates whether observed differences between human and AI scores align with the expected variations under simulated conditions. The discussion section synthesizes simulation and empirical results, highlighting metric-specific mechanisms and fairness considerations. The article concludes with actionable recommendations and acknowledged limitations.

## 2 Materials and methods

### 2.1 Research model

This study used Monte Carlo simulation to examine the performance of commonly used human-AI agreement metrics under systematically created scoring distortions. Under simulation conditions, we controlled manipulated additive bias, variance inflation, midpoint compression, class imbalance, and subgroup-related offsets (error structures that cannot be clearly isolated in empirical scoring datasets) (Ercikan and McCaffrey, 2022; Debelak and Ziegler, 2025). Threshold-exceedance performance, defined as the proportion of iterations in which each agreement metric exceeded the conventional adequacy criterion, was evaluated.

### 2.2 Simulation factors

Six simulation factors were manipulated in a fully crossed design (3 × 3 × 3 × 2 × 2 × 2), yielding 216 distinct simulation conditions. Five of these factors corresponded to scoring distortions documented in prior empirical and methodological research. (1) *Systematic bias* was included because AI-generated scores may consistently deviate upward or downward relative to human ratings (Quah et al., 2024). In such cases, Bland–Altman and tolerance-based overlap analyses become essential complements to global coefficients, as they are more sensitive to directional shifts (Giavarina, 2015). (2) *Construct-irrelevant variance* captured situations in which the error distribution of AI scores exhibits systematically narrower or wider variance compared to human scores, a pattern reported in recent evaluations of automated scoring tools (Zhai et al., 2021). (3) *Mid-point compression* modeled the tendency of automated systems to avoid extreme score values, as discussed by Yiğiter and Boduroğlu (2025). (4) *Class imbalance (prevalence effect)* reflected the well-established finding that scoring accuracy decreases at the upper and lower ends of performance distributions (Bexkens et al., 2018; Uysal and Doğan, 2021). (5) *Group-based bias (fairness issue)* incorporated systematic distortion within a designated subgroup, a mechanism emphasized in fairness-oriented assessment research (Johnson et al., 2022; Debelak and Ziegler, 2025). In practice, subgroup residual profiles and group-specific tolerance-overlap summaries are recommended to prevent global coefficients such as ICC or QWK from masking directional disparities (Johnson and McCaffrey, 2023).

All simulation conditions were replicated 5,000 times to reduce Monte Carlo error, in line with recommendations for achieving stable estimates—particularly for ratio-based indices (Mundform et al., 2011).

### 2.3 Data-generating model latent variable and human score generation

#### 2.3.1 Latent true scores

For each replication, latent ability values:

θ_*i*_,*i* = 1, *N* were drawn from a standard normal distribution. The use of a normal latent structure aligns with established practice in rater-mediated assessment simulations (Zhai et al., 2021), as it provides a stable and well-behaved variance framework from which subsequent human and AI scoring processes can be derived. This distributional adjustment keeps the variance of the baseline ability level constant across all simulation conditions. This allows for a purer picture of the effect of a different condition added to the model, thus ensuring comparability across conditions.

#### 2.3.2 Human scoring model

Human ratings were generated using an additive noise framework of the form


$$H_{i}=\theta_{i}+\varepsilon_{i},$$

where ε_*i*_ represents human rater error. This structure reflects the variability and inconsistency of human ratings (Bıkmaz Bilgen and Doğan, 2017; Hogan and Murphy, 2007). The scores generated at this stage were left on a continuous scale. This approach ensures that all condition structures are preserved before discretization.

#### 2.3.3 AI scoring model and distortion mechanisms

AI scores were generated from the same latent ability values used in human ratings. Systematic manipulations were applied to empirically observed patterns of human-AI mismatch (Giavarina, 2015; Hayes and Krippendorff, 2007; Ferrara and Qunbar, 2022). For each individual, latent AI scores were defined as follows:


$$A_{i}^{*}=\theta_{i}+b+\varepsilon_{i}^{\left(A\right)},$$

where *b* ∈ {−0.5,0, + 0.5}is an additive bias term that shifts the AI cores up or down. $\varepsilon_{i}^{\left(A\right)}$is an AI-specific random error term with mean zero and conditional standard deviation σ_*A*_ ∈ {0.40,0.50,0.95}, corresponding to low, medium and high AI error variance levels, respectively. In the next step, before discretization, all AI scores were subjected to other error conditions (midpoint compression, variance inflation, and subgroup offsets). In this context, human scores were used as a reference to assess the validity and reliability of AI-based scores, consistent with previous research on similar performance assessment (Kersting et al., 2014).

### 2.4. Additional distortion structures

#### 2.4.1 Midpoint compression

The compression manipulation was used to reflect the tendency of automated scoring technologies to avoid extreme values. Under these conditions, latent AI scores were shrunk toward the center of the scale:


$$A_{i}^{\left(\text{comp}\right)}=\left(1-\lambda_{c}\right)\,A_{i},$$ (1)

where λ_*c*_ = 0.40 denotes the compression intensity. When compression was absent, $A_{i}^{\left(\text{comp}\right)}=A_{i}$. This transformation reduces the spread of AI scores while preserving their rank ordering, thereby mimicking empirically observed central-tendency behavior in automated scoring.

#### 2.4.2 Class imbalance

To represent prevalence effects observed in performance assessments, an imbalance mechanism was applied prior to discretization. Under imbalance conditions, the continuous AI score distribution was reshaped so that the lower portion of the distribution (approximately 40%) was shifted downward and the upper portion (approximately 10%) was shifted upward. This adjustment produces skewed category probabilities and reflects standard additive–multiplicative distortion structures used in psychometric simulation studies (Camilli, 2006; Brookhart, 2010; Kersting et al., 2014).

#### 2.4.3 Group-based bias (fairness distortion)

A binary subgroup indicator *G*_*i*_ ∈ {0,1}was randomly assigned with equal probability. To introduce systematic subgroup deviation, an offset was added to the AI score for one group:


$$A_{i}^{\left(\text{fair}\right)}=A_{i}+\delta \,G_{i},$$

where δ = 0.30 represents the magnitude of the imposed subgroup bias. This structure parallels fairness-related distortions discussed in assessment research (Ferrara and Qunbar, 2022; Johnson and McCaffrey, 2023; Schaller et al., 2024).

#### 2.4.4 Discretization to the ordinal scoring scale

Following the application of distortion mechanisms, both human and AI latent scores were mapped onto an ordered rating scale with categories *0-4* using fixed cut-points on the latent continuum. Under balanced conditions, cut-points were placed at –0.8, –0.2, 0.2, and 0.8, whereas under imbalance conditions they were shifted to –1.2, –0.4, 0.4, and 1.2. This discretization ensured that observed discrepancies between human and AI ratings arose from the specified distortion structures rather than from arbitrary rescaling procedures.

#### 2.4.5 Agreement metrics

Three primary indices were computed in each replication to summarize agreement between human and AI ratings on the discretized rubric.

#### 2.4.6 ICC(A,1)

Absolute-agreement intraclass correlation was estimated in the two-way mixed, single-measurement framework, denoted as ICC(A,1) (Shrout and Fleiss, 1979). For two raters, the coefficient was computed as:


$$\text{ICC}\,\left(A,1\right)=\frac{M\,S_{B}-M\,S_{W}}{M\,S_{B}+\left(k-1\right)\,M\,S_{W}},$$

where MS_*B*_ is the between-target mean square, *MS_W_*is the within-target (residual) mean square, and k = 2 is the number of raters (human vs. AI). This form corresponds to an absolute-agreement index, penalizing both level and rank discrepancies between the two rating sources.

#### 2.4.7 Krippendorff’s alpha (ordinal)

Ordinal Krippendorff’s alpha was used to quantify agreement while respecting the ordered nature of rubric categories (Hayes and Krippendorff, 2007). It is defined as:


$$\alpha =1-\frac{D_{o}}{D_{e}},$$ (2)

where *D_o_*denotes the observed disagreement among ratings and *D_e_*denotes the disagreement expected by chance under the assumption of random assignment of categories. Both *D_o_* and *D_e_*were computed using an ordinal distance function that assigns larger penalties to discrepancies between more distant score categories, in line with the implementation of ordinal alpha in the simulation code.

#### 2.4.8 Quadratic weighted kappa

Quadratic weighted kappa was used to summarize agreement between human and AI ratings on the ordinal rubric. It is defined as:


$$\kappa_{w}=1-\frac{\sum_{i}\sum_{j}w_{i\,j}\,O_{i\,j}}{\sum_{i}\sum_{j}w_{i\,j}\,E_{i\,j}}$$

where *O*_*ij*_ and *E*_*ij*_ denote the observed and expected proportions of ratings in category pair (*i*,*j*), respectively, and *w*_*ij*_ is a weight reflecting the squared-distance penalty between categories *i* and *j*. Larger discrepancies between score categories therefore receive disproportionately higher penalties, making QWK particularly suitable for ordered scoring scales (Bexkens et al., 2018).

#### 2.4.9 Bland–Altman indicators

Bland–Altman analysis was employed to characterize systematic and random components of disagreement between human and AI scores (Giavarina, 2015). For each examinee *i*, the difference score was defined as:


$$d_{i}=H_{i}-A_{i},$$

where *H_i_*is the human score and *A_i_*is the AI score. The mean difference (bias) was computed as:


$$\bar{d}=\frac{1}{N}\,\overset{N}{\underset{i=1}{\sum }}d_{i},$$

and the standard deviation of the differences as:


$$s_{d}=\sqrt{\frac{1}{N-1}\,\overset{N}{\underset{i=1}{\sum }}{\left(d_{i}-\bar{d}\right)}^{2}}.$$

The 95% limits of agreement (LoA) were then obtained as:


$${\text{LoA}}_{\text{lower}}=\bar{d}-1.96\,s_{d},{\text{LoA}}_{\text{upper}}=\bar{d}+1.96\,s_{d}.$$

Here, **d_i_** denotes the signed human–AI difference for examinee *i*, $\bar{d}$represents the average bias across examinees, *s_d_*indicates the variability of these differences, and the LoA provide the range within which approximately 95% of human–AI discrepancies are expected to fall under a normality assumption.

#### 2.4.10 Tolerance-based agreement

Tolerance-based agreement offers an interpretable correctness criterion by quantifying the proportion of human–AI score pairs that fall within a prespecified difference band (often ± 1 score point in educational assessment; Williamson et al., 2012). For a tolerance width *k* = 1, the tolerance-based agreement index was computed as:


$$T=\frac{1}{N}\overset{N}{\underset{i=1}{\sum }}I\left(|H_{i}-A_{i}|\leq k\right),$$

where **I**(⋅) is an indicator function that equals 1 if the condition is satisfied and 0 otherwise, *H_i_*is the human score, *A_i_*is the AI score, and |*H*_*i*_−*A*_*i*_| is the absolute human–AI difference for examinee **i**. Thus, **T**represents the proportion of examinees whose AI scores fall within one rubric point of their corresponding human ratings.

### 2.5 Threshold-exceedance framework

#### 2.5.1 Threshold-exceedance definition

Threshold-exceedance was defined as the proportion of replications in which an agreement metric surpassed its prespecified adequacy threshold. Consistent with established guidelines, the following cut-offs were applied:

- ICC(A,1) ≥ 0.70 (Koo and Li, 2016; Liljequist et al., 2019)
- Krippendorff’s α ≥ 0.67 for ordinal data (Hayes and Krippendorff, 2007)
- Quadratic weighted kappa (QWK) ≥ 0.60, reflecting moderate-to-substantial ordinal agreement (Uto, 2021; Quah et al., 2024), with an additional constraint that the AI–human QWK remained within 0.10 of the corresponding human–human QWK
- Tolerance-based agreement ≥ 70%, defined as the proportion of examinees for whom AI scores fell within one rubric point of human ratings (Williamson et al., 2012)

These thresholds were used consistently in both operating-performance analyses and Type I error evaluation.

#### 2.5.2 Type I error under the strict null condition

Type I error rates were computed under a strict null configuration in which no intentional distortion was applied to the AI scoring model. In this setting:

- bias = 0,
- ai_var = “mid”, aligning AI error variance with human rater variance,
- compression = FALSE,
- imbalance = FALSE,
- fairness = FALSE.

Under this configuration, AI functions as an additional rater differing from the human reference only through random error, consistent with the simulation code. For each metric **m**, the Type I error rate was defined as the proportion of replications in which the threshold-exceedance indicator equaled 1 under the strict null—that is, the metric falsely exceeded its adequacy threshold despite the absence of bias, variance distortion, compression, imbalance, or fairness effects.

For each metric, Type I error rates and their Wilson confidence intervals were extracted from the replications corresponding to the strict null condition.

#### 2.5.3 Threshold-sensitivity analysis

To assess the robustness of threshold-exceedance conclusions, a threshold-sensitivity analysis was conducted for ICC(A,1), Krippendorff’s α, and QWK. Beginning from baseline thresholds, a grid of alternative cut-offs was defined at ± 0.05 and ± 0.10:

- ICC(A,1): 0.60, 0.65, 0.70, 0.75, 0.80
- Krippendorff’s α: 0.70, 0.75, 0.80, 0.85, 0.90
- QWK: 0.60, 0.65, 0.70, 0.75, 0.80 (with the 0.10 gap constraint applied at all thresholds)

For each metric–threshold pair, agreement statistics were recomputed from the stored Parquet datasets, and threshold-exceedance indicators were recalculated for every candidate cut-off across all 216 simulation conditions.

Under the strict null configuration, these threshold-specific success proportions represent Type I error as a function of the cut-off.

Under distorted conditions, they represent operating-performance robustness.

Rank-order stability was assessed by comparing the ordering of metrics at each alternative threshold against the baseline ordering. The proportion of conditions in which the metric ordering changed served as an overall indicator of threshold robustness. Full threshold-sensitivity outputs are reported in Supplementary material.

### 2.6 Statistical analyses

#### 2.6.1 Logistic regression models

Two logistic modeling frameworks were applied to examine how simulation factors influenced whether each agreement metric met its adequacy criterion.

##### 2.6.1.1 Single-metric models

A separate logistic regression was fitted for each metric, modeling the probability that metric **m** exceeded its threshold in a given replication:


$$\text{Pr}\left(Y_{i}^{\left(m\right)}=1\right)={\text{logit}}^{-1},(\beta_{0}+\beta_{1}N+\beta_{2}b+\beta_{3}\text{var}+$$

$$\beta_{4}\text{comp}+\beta_{5}\text{imb}+\beta_{6}\text{fair}),$$

where ${\text{Y}}_{i}^{\left(m\right)}=1$ indicates that metric **m** achieved its predefined threshold in replication **i**; **N**, **b**, variance level, compression, imbalance, and fairness indicators correspond to the manipulated simulation factors.

##### 2.6.1.2 Composite model

A combined model was also estimated in which the three agreement metrics were included as fixed effects within the same logistic framework. This model allowed direct comparison of relative metric performance while adjusting for identical simulation conditions.

Each metric was evaluated across 216 conditions × 5,000 replications = 1,080,000 observations. Thus:

- Single-metric models included 1,080,000 rows per metric.
- The composite model included 3 × 1,080,000 = 3,240,000 metric–condition pairs.

#### 2.6.2 Holm correction

The Holm–Bonferroni procedure was applied to maintain family-wise Type I error control across multiple comparisons involving simulation factors and metric effects. The Holm method was selected because it is uniformly more powerful than the classical Bonferroni adjustment while preserving strict FWER control. Its suitability for studies comparing agreement metrics through logistic regression has been demonstrated in prior work (Kim and Oshima, 2012).

#### 2.6.3 Confidence intervals: Wilson method

Threshold-exceedance proportions are binomial quantities. Because standard Wald intervals exhibit poor performance when estimated probabilities approach 0 or 1—an expected scenario given the extreme success rates observed in several conditions—the Wilson interval (Brown et al., 2001) was used.

For an observed proportion $\hat{\text{p}}$ based on **n** replications, the Wilson point estimate is


$${\hat{p}}_{W}=\frac{\hat{p}+z^{2}/\left(2\,n\right)}{1+z^{2}/n},$$

with symmetric half-width


$$S\,E_{W}=\frac{z\,\sqrt{\hat{p}\,\left(1-\hat{p}\right)/n+z^{2}/\left(4\,n^{2}\right)}}{1+z^{2}/n}.$$

These intervals provide more accurate coverage than Wald intervals, particularly when proportions are close to the boundaries of 0 or 1.

### 2.7 Real scoring data validation

#### 2.7.1 Data source

Six B2-level student essays were obtained from a previously completed instructional task. Each essay was independently scored by three human teachers who differed in their familiarity with the students (direct instructor, indirectly familiar teacher, unfamiliar teacher). Two AI-based scoring systems (ChatGPT and Gemini) also assigned scores using the same analytic rubric (0–70 points) applied in the simulation framework. This produced

- 18 human scores (6 essays × 3 teachers),
- 12 AI-generated scores (6 essays × 2 systems).

The human reference score for each essay was defined as the arithmetic mean of the three teacher ratings, consistent with established practice in rater-mediated assessment research, where the mean of multiple raters is commonly treated as an operational benchmark.

#### 2.7.2 Analytic indicators

To ensure alignment with the simulation’s distortion mechanisms, three core discrepancy indicators were estimated for each AI system:

1. Bias (additive deviation):


$$\text{Bias}=Y_{i}^{A\,I}-Y_{i}^{r\,e\,f},$$

where ${\text{Y}}_{i}^{r\,e\,f}$

is the human reference score.

2. Variance ratio (relative variability):


$$\mathrm{Variance}\,\mathrm{Ratio}=\frac{S\,D\,\left(Y^{A\,I}-Y^{r\,e\,f}\right)}{S\,D\,\left(Y^{t\,e\,a\,c\,h\,e\,r}-Y^{r\,e\,f}\right)}.$$

3. Slope of regression (compression indicator):


$$Y_{i}^{A\,I}=\beta_{0}+\beta_{1}\,Y_{i}^{r\,e\,f}+\varepsilon_{i}.$$

A slope of β_1_ = corresponds to midpoint compression, reflecting the compression mechanism.

These indicators map directly to the additive bias, multiplicative/variance (variance ratio), and ponds to mid-point compression, mirroring the simulation’s compression mechanism.

#### 2.7.3 Bootstrap estimation

Since sample size of real data as very small, all statistics were estimated more reliably with 2000 bootstrap replication. Bootstrap resampling produced:

1. 95% confidence intervals for bias, variance ratio, and slope coefficients;
2. Bootstrap-estimated probabilities of:
3. compression *Pr*(β_1_ = 1),
4. imbalance (prevalence shifts in score distribution),
5. fairness deviations (systematic differences across teacher-familiarity groups).

This distribution-free approach is appropriate for small samples and directly parallels the model-free discrepancy estimation structure used in the simulation.

#### 2.7.4 Subgroup fairness assessment

Group-based conditions were calculated as follows for evaluating fairness models:


$$d_{g\,\left(i\right)}=Y_{i}^{A\,I}-Y_{g\,\left(i\right)}^{H},$$

where *g*(*i*) indexes the teacher-familiarity group (direct instructor, indirectly familiar teacher, unfamiliar teacher). Bootstrap resampling yielded:

1. the mean deviation for each group,
2. the probability of ordered deviations (e.g., Known < Familiar < Unknown),
3. the estimated magnitude of expected group gaps.

These procedures ensure conceptual fit between comparisons of fairness models in the simulation model and empirical subgroups.

#### 2.7.5 Alignment with simulation conditions

For each AI system, the resulting empirical conditions were matched to the closest corresponding simulation condition. This allowed evaluation of whether the real-data scoring behavior resembled the simulation patterns generated by the additive-bias, variance-distortion, and compression mechanisms. The detail metrics for the AI systems and their matchings findings with the simulation conditions are given in Table 1.

**TABLE 1:** Bootstrap-estimated metrics and correspondence with simulation conditions.

AI system	Bias (points, 95% CI)	VarRatio (95% CI)	Slope (95% CI)	*R* ^2^	*P* (Compression)	*P* (Fairness)	Simulation match
GPT	−9.00 [-14.67, −1.22]	0.84 [0.21, 1.53]	0.50 [0.16, 0.87]	0.79	0.99	0.998	*N* = 100, Bias = -0.5, Var = medium, Compression = present, Fairness = present
Gemini	−12.05 [-20.44, −0.83]	1.24 [0.34, 2.39]	0.67 [-0.12, 1.60]	0.48	0.69	0.999	*N* = 100, Bias = -0.5, Var = high, Compression = present, Fairness = present

Bias = AI score minus human reference; VarRatio = SD(AI − Ref)/SD(Teacher − Ref); probabilities from bootstrap (*B* = 2,000).

#### 2.7.6 Software

All analyses were conducted in R 4.3.2 (R Core Team, 2024) using:

1. simresearch (Goldfeld and Wujciak-Jens, 2024)
2. irr (Gamer et al., 2019)
3. psych (Revelle, 2024)
4. BlandAltmanLeh (Lehnert, 2020)
5. DescTools (Signorell, 2023)
6. tidyverse (Wickham et al., 2019)
7. Simulation code, seeds, and output files are archived in the project repository.

## 3 Results

In this section, the analysis results performed on the simulation and real data of the study are included.

### 3.1 Descriptive metric behavior under simulation conditions

#### 3.1.1 Descriptive metric behavior across simulation conditions

The descriptive statistical results for the fit indices calculated in this section provide preliminary information regarding parameter-dependent variability. It is anticipated that these results will form the basis for further analyses. Supplementary Table 1 provides all mean results related to these descriptive patterns.

Table 2 displays the mean values of the four agreement metrics for all crossed conditions. As AI variance increases, mean agreement values decrease systematically for every metric. Larger sample sizes correspond to higher mean ICC(A,1) and QWK values due to reduced sampling variability, whereas Krippendorff’s α remains low across all conditions. Conditions with additive bias (-0.5 or +0.5) yield lower mean agreement compared to the unbiased scenario. Tolerance-based agreement remains numerically higher than the three coefficient-based metrics across nearly all cells.

**TABLE 2:** Mean values of agreement metrics (ICC, Krippendorff’s α, QWK) across simulation conditions.

N	Bias	AI Var	ICC(A,1)	Kripp. α	QWK	Tol ± 1	Tol ± 2
100	−0.5	Low	0.682	0.667	0.680	0.794	0.946
100	−0.5	Mid	0.657	0.641	0.654	0.778	0.935
100	−0.5	High	0.531	0.514	0.529	0.705	0.875
100	0.0	Low	0.772	0.771	0.771	0.866	0.977
100	0.0	Mid	0.740	0.739	0.738	0.841	0.967
100	0.0	High	0.578	0.579	0.575	0.734	0.897
100	0.5	Low	0.683	0.667	0.680	0.794	0.947
100	0.5	Mid	0.657	0.640	0.654	0.778	0.935
100	0.5	High	0.533	0.515	0.530	0.707	0.876
300	−0.5	Low	0.682	0.669	0.681	0.793	0.946
300	−0.5	Mid	0.659	0.645	0.658	0.779	0.935
300	−0.5	High	0.534	0.518	0.533	0.707	0.876
300	0.0	Low	0.773	0.774	0.773	0.866	0.978
300	0.0	Mid	0.740	0.741	0.739	0.840	0.966
300	0.0	High	0.576	0.580	0.576	0.732	0.897
300	0.5	Low	0.682	0.670	0.681	0.793	0.946
300	0.5	Mid	0.659	0.644	0.658	0.779	0.935
300	0.5	High	0.534	0.518	0.533	0.707	0.876
1000	−0.5	Low	0.682	0.670	0.682	0.793	0.946
1000	−0.5	Mid	0.658	0.645	0.658	0.779	0.935
1000	−0.5	High	0.534	0.519	0.533	0.706	0.876
1000	0.0	Low	0.774	0.775	0.774	0.866	0.978
1000	0.0	Mid	0.740	0.742	0.740	0.840	0.966
1000	0.0	High	0.576	0.581	0.576	0.732	0.896
1000	0.5	Low	0.683	0.671	0.682	0.794	0.946
1000	0.5	Mid	0.658	0.645	0.658	0.779	0.935
1000	0.5	High	0.533	0.518	0.533	0.706	0.875

#### 3.1.2 Type I error (null liberality) under perfect agreement conditions

This subsection reports the false threshold-exceedance frequencies under strict null conditions: no bias, matched variances, no compression, no imbalance, and no subgroup offsets. These results are presented before threshold-exceedance analyses to ensure the interpretability of subsequent operational conclusions.

Table 3 shows that ICC(A,1) and QWK exceed their adequacy thresholds in a large proportion of replications under ideal no-distortion conditions. Supplementary Table 2 presents the summary of Type I errors, detailing false threshold-exceedance performance for strict null conditions. Krippendorff’s α rarely meets its adequacy threshold, with values near zero. These baseline patterns provide reference points for interpreting subsequent threshold-exceedance results.

**TABLE 3:** False threshold exceedance under null (liberality index; null scenarios).

N	Metric	Type I	Lower CI	Upper CI	MCSE
100	ICC(A,1)	0.792	0.780	0.803	0.006
100	Krippendorff’s α	0.099	0.091	0.108	0.004
100	QWK	0.744	0.731	0.756	0.006
300	ICC(A,1)	0.912	0.903	0.919	0.004
300	Krippendorff’s α	0.010	0.008	0.013	0.001
300	QWK	0.907	0.899	0.915	0.004
1,000	ICC(A,1)	0.993	0.991	0.995	0.001
1,000	Krippendorff’s α	0.000	0.000	0.000	0.000
1,000	QWK	0.993	0.990	0.995	0.001

#### 3.1.3 Threshold-exceedance proportions across AI variance and sample size

This subsection presents threshold-exceedance proportions under non-null conditions. These values quantify each metric’s probability of meeting its adequacy threshold under realistic error structures. Supplementary Table 3 reports the full power estimates for all crossed bias–variance–sample-size conditions.

Table 4 indicates that all metrics achieve their highest threshold-exceedance rates under low variance and progressively lower success rates under medium and high variance. Increased sample size increases threshold attainment for ICC(A,1) and QWK under low and medium variance, whereas high variance drives probabilities close to zero regardless of N. Krippendorff’s α demonstrates the lowest success rates across all conditions.

**TABLE 4:** Threshold-exceedance proportions by metric and condition.

N	AI Var	Metric	Threshold-exceedance proportion	Lower CI	Upper CI	MCSE
100	Low	ICC(A,1)	0.941	0.934	0.947	0.003
100	Low	Krippendorff’s α	0.261	0.249	0.274	0.006
100	Low	QWK	0.860	0.851	0.870	0.005
100	Mid	ICC(A,1)	0.792	0.780	0.803	0.006
100	Mid	Krippendorff’s α	0.099	0.091	0.108	0.004
100	Mid	QWK	0.744	0.731	0.756	0.006
100	High	ICC(A,1)	0.035	0.030	0.041	0.003
100	High	Krippendorff’s α	0.000	0.000	0.001	0.000
100	High	QWK	0.028	0.024	0.033	0.002
300	Low	ICC(A,1)	0.998	0.996	0.999	0.001
300	Low	Krippendorff’s α	0.147	0.138	0.157	0.005
300	Low	QWK	0.988	0.985	0.991	0.002
300	Mid	ICC(A,1)	0.912	0.903	0.919	0.004
300	Mid	Krippendorff’s α	0.010	0.008	0.013	0.001
300	Mid	QWK	0.907	0.899	0.915	0.004
300	High	ICC(A,1)	0.001	0.000	0.002	0.000
300	High	Krippendorff’s α	0.000	0.000	0.001	0.000
300	High	QWK	0.001	0.000	0.002	0.000
1,000	Low	ICC(A,1)	1.000	0.999	1.000	0.000
1,000	Low	Krippendorff’s α	0.032	0.027	0.037	0.002
1,000	Low	QWK	1.000	0.999	1.000	0.000
1,000	Mid	ICC(A,1)	0.993	0.991	0.995	0.001
1,000	Mid	Krippendorff’s α	0.000	0.000	0.001	0.000
1,000	Mid	QWK	0.993	0.990	0.995	0.001
1,000	High	ICC(A,1)	0.000	0.000	0.001	0.000
1,000	High	Krippendorff’s α	0.000	0.000	0.001	0.000
1,000	High	QWK	0.000	0.000	0.001	0.000

All three agreement metrics showed a consistent decrease in threshold-exceedance as the proficiency threshold increased. The results showed that ICC(A,1) rarely met the more stringent criteria under simulated conditions. Krippendorff’s α (ordinal) showed an even sharper decrease. QWK showed a similar monotonic decrease. All these results indicate moderate robustness for flexible thresholds but limited exceedance at more stringent thresholds. The decreases in standard deviation with increasing thresholds suggest that exceedance performance is limited to a small subset of stringent thresholds.

### 3.2 Logistic regression models for single metrics

This section presents the results of logistic regressions that estimate the probability of exceeding the adequacy threshold for each metric. Supplementary Table 4, involves the coefficient estimates for whole single-metric models. These models reveal the contribution of each condition to threshold-exceedance performance.

According to Table 5, since the obtained coefficients are negative, the probability of threshold-exceedance performance decreases as the sample size increases (100 → 300 → 1,000). The bias conditions, on the other hand, show a strong negative relationship for all metrics. In other words, they reflect a decrease in threshold-exceedance performance when systematic distortion is present. Conditions with medium and large AI error variance levels produced negative effects for ICC(A,1) and Krippendorff’s α. Similarly, compression and imbalance exhibited negative coefficients for all metrics, indicating decreased success rates. Manipulations in the fairness conditions produced small positive coefficients, indicating a small increase in the probability of exceedance. All effects were statistically significant under Holm’s adjustment.

**TABLE 5:** Logistic regression coefficients predicting threshold-exceedance success by agreement metric.

Metric	Value	β	SE	Lower CI	Upper CI	p (Holm)
ICC(A,1)	Factor(N)300	−0.232	0.007	−0.245	−0.219	0.000
ICC(A,1)	Factor(N)1000	−0.352	0.007	−0.365	−0.339	0.000
ICC(A,1)	Bias	−0.918	0.007	−0.931	−0.904	0.000
ICC(A,1)	factor(ai_var)mid	−0.831	0.006	−0.842	−0.820	0.000
ICC(A,1)	Factor(ai_var)high	−5.758	0.032	−5.821	−5.694	0.000
ICC(A,1)	Compression = TRUE	−0.955	0.006	−0.966	−0.944	0.000
ICC(A,1)	Imbalance = TRUE	−1.251	0.006	−1.262	−1.239	0.000
ICC(A,1)	Fairness = TRUE	0.094	0.006	0.083	0.104	0.000
Krippendorff’s α	Factor(N)300	−1.541	0.034	−1.607	−1.476	0.000
Krippendorff’s α	Factor(N)1000	−3.473	0.080	−3.629	−3.317	0.000
Krippendorff’s α	Bias	−0.202	0.032	−0.265	−0.140	0.000
Krippendorff’s α	Factor(ai_var)mid	−1.466	0.032	−1.529	−1.402	0.000
Krippendorff’s α	Factor(ai_var)high	−7.893	0.707	−9.279	−6.507	0.000
Krippendorff’s α	Compression = TRUE	−1.729	0.035	−1.798	−1.660	0.000
Krippendorff’s α	Imbalance = TRUE	−1.657	0.034	−1.724	−1.589	0.000
Krippendorff’s α	Fairness = TRUE	−0.606	0.027	−0.659	−0.553	0.000
QWK	Factor(N)300	−0.108	0.007	−0.122	−0.095	0.000
QWK	Factor(N)1000	−0.209	0.007	−0.222	−0.196	0.000
QWK	Bias	−0.906	0.007	−0.919	−0.892	0.000
QWK	Factor(ai_var)mid	−0.831	0.006	−0.842	−0.820	0.000
QWK	Factor(ai_var)high	−5.938	0.036	−6.009	−5.866	0.000
QWK	Compression = TRUE	−0.957	0.006	−0.968	−0.946	0.000
QWK	Imbalance = TRUE	−1.247	0.006	−1.259	−1.236	0.000
QWK	Fairness = TRUE	0.107	0.006	0.096	0.118	0.000

### 3.3 Composite logistic model: cross-metric predictors of threshold crossing

This section discusses the behavior of ICC(A,1), Krippendorff α, and QWK within a combined multivariate framework. The composite model estimates threshold crossing probabilities by controlling for shared variance among metrics and the full set of simulation conditions. All details results are reported in Supplementary Table 5.

The results presented in Table 6 indicate that the negative coefficients for Krippendorff’s α and QWK indicate that these metrics are associated with lower threshold-exceedance performance compared to ICC(A,1). The sample size indicators are similar to those in the individual model studies. Furthermore, the negative coefficients found for Systematic bias, AI variance–mid, and especially AI variance–high indicate that threshold-exceedance performance decreases under these conditions. Compression and imbalance conditions also exert similar negative effects. The positive coefficient for the Fairness variable can be interpreted as being associated with a smaller increase compared to the other conditions.

**TABLE 6:** Logistic regression coefficients predicting threshold-exceedance proportions across all metrics.

Value	β	SE	Lower CI	Upper CI	p (Holm)
(Intercept)	0.732	0.007	0.718	0.746	0.000
Krippendorff’s α (ordinal)	−2.757	0.022	−2.801	−2.714	0.000
Quadratic weighted kappa (QWK)	−0.155	0.010	−0.175	−0.136	0.000
*N* = 300	−0.232	0.007	−0.245	−0.219	0.000
*N* = 1,000	−0.352	0.007	−0.365	−0.339	0.000
Systematic bias	−0.918	0.007	−0.931	−0.904	0.000
AI variance = mid	−0.831	0.006	−0.842	−0.820	0.000
AI variance = high	−5.758	0.032	−5.821	−5.694	0.000
Compression = true	−0.955	0.006	−0.966	−0.944	0.000
Imbalance = true	−1.251	0.006	−1.262	−1.239	0.000
Fairness = true	0.094	0.006	0.083	0.104	0.000
Krippendorff’s α × sample size (300)	−1.309	0.034	−1.376	−1.242	0.000
QWK × sample size (300)	0.123	0.009	0.105	0.142	0.000
Krippendorff’s α × sample size (1,000)	−3.121	0.080	−3.278	−2.965	0.000
QWK × sample size (1,000)	0.143	0.010	0.124	0.162	0.000
Krippendorff’s α × bias	0.715	0.033	0.651	0.779	0.000
QWK × bias	0.012	0.010	−0.007	0.031	0.000
Krippendorff’s α × AI variance (mid)	−0.635	0.033	−0.700	−0.571	0.000
QWK × AI variance (mid)	−0.000	0.008	−0.016	0.015	0.000
Krippendorff’s α × AI variance (high)	−2.135	0.696	−3.500	−0.770	0.000
QWK × AI variance (high)	−0.180	0.049	−0.276	−0.085	0.000
Krippendorff’s α × compression	−0.774	0.036	−0.844	−0.704	0.000
QWK × compression	−0.002	0.008	−0.017	0.014	0.000
Krippendorff’s α × imbalance	−0.406	0.035	−0.474	−0.338	0.000
QWK × imbalance	0.003	0.008	−0.013	0.019	0.000
Krippendorff’s α × fairness	−0.700	0.028	−0.754	−0.646	0.000
QWK × fairness	0.013	0.008	−0.002	0.028	0.000

According to the interaction results, the negative values of the Krippendorff’s α × Sample Size (300/1,000) and Krippendorff’s α × AI Variance (High) coefficients indicate that the threshold-exceedance performance of α decreases further under these conditions. The coefficients calculated for QWK range from positive to negative. This is evidence that the effects for this metric change direction depending on the condition. All coefficients in the table are significant under the Holm correction.

#### 3.3.1 Wilson coverage verification under null conditions (*N* = 300)

The Wilson method was used because it is more reliable than the Wald interval for binomial odds, especially when success rates are very close to 0 or 1.

Wilson 95% coverage rates were calculated for *N* = 300 under null conditions (bias = 0; no variance shift; no compression, imbalance, or fairness). All details results are reported in Supplementary Table 6. Obtaining coverage close to the nominal 95% level for all metrics demonstrates that the interval estimation procedures are statistically appropriate and do not inflate or suppress threshold exceedance rates.

#### Heatmap diagnostics across all design conditions

Heat maps used to visualize threshold exceedance patterns reveal high-dimensional patterns of metric sensitivity. This section provides a collective interpretation of the heat maps.

#### 3.3.2 ICC(A,1) heat maps

**FIGURE 1:**
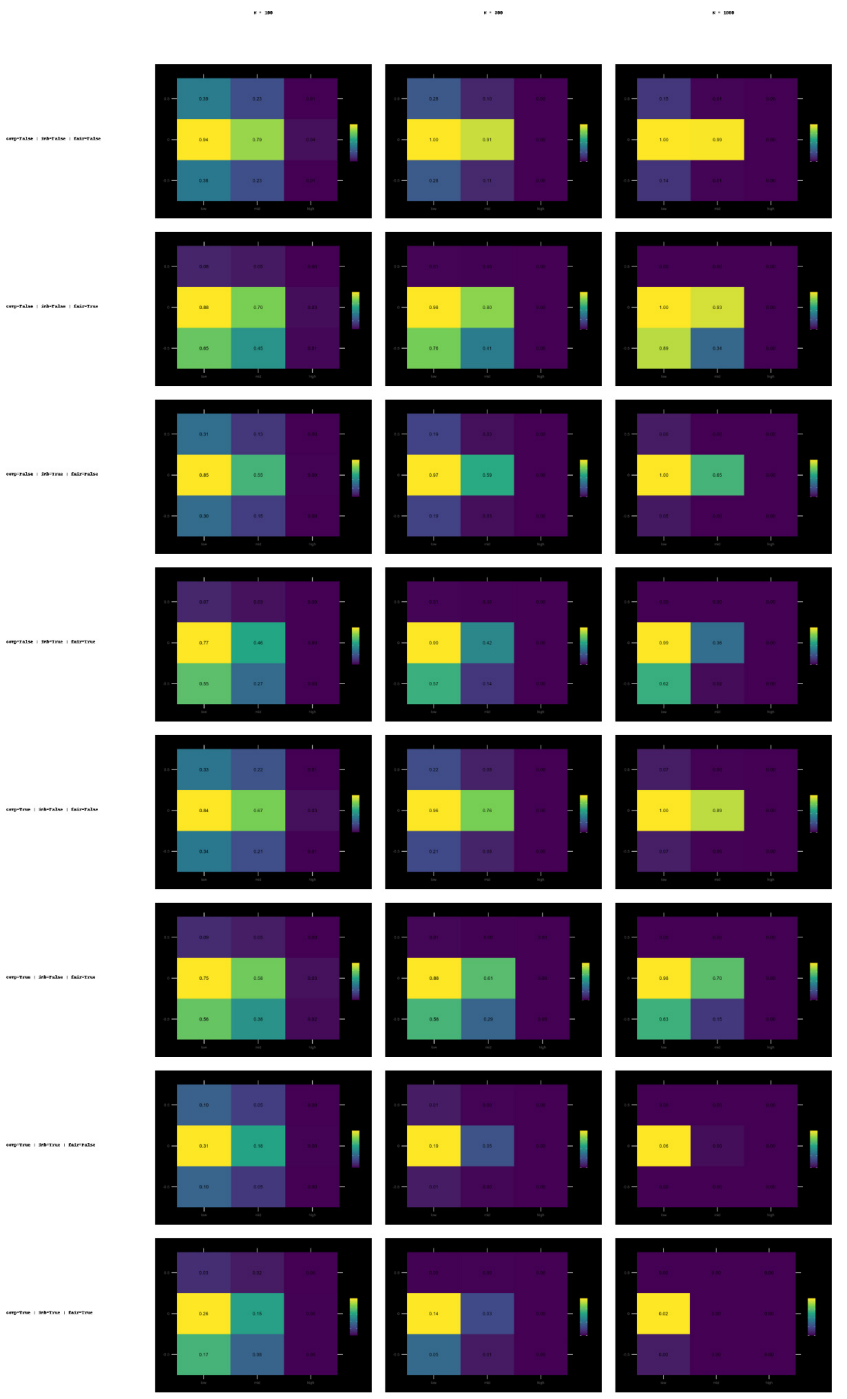
Heat maps of success rate values for ICC(A,1) with different sample sizes (*N* = 100, 300, 1,000) and comp × imbalance × fairness conditions. Heatmaps of ICC(A,1) success rate values across sample sizes (100, 300, 1,000) and error scenarios (compression, imbalance, fairness). Darker tones indicate higher success rate.

The ICC(A,1) heatmaps (Figure 1) exhibit a clear gradient across sample sizes. Supplementary Figure 1 consist of all ICC(A,1) heatmaps for all simulation conditions.

1. For *N* = 100, the results are statistically more scattered. Because variability is high for this condition, the results are more unstable. With compression, imbalance effects are more pronounced.
2. For *N* = 300 and *N* = 1,000, the threshold-exceedance ratios are both higher and concentrated within a narrower range. This suggests that ICC(A,1) is more robust to moderate disturbances.

#### 3.3.3 Krippendorff’s α heat maps

**FIGURE 2:**
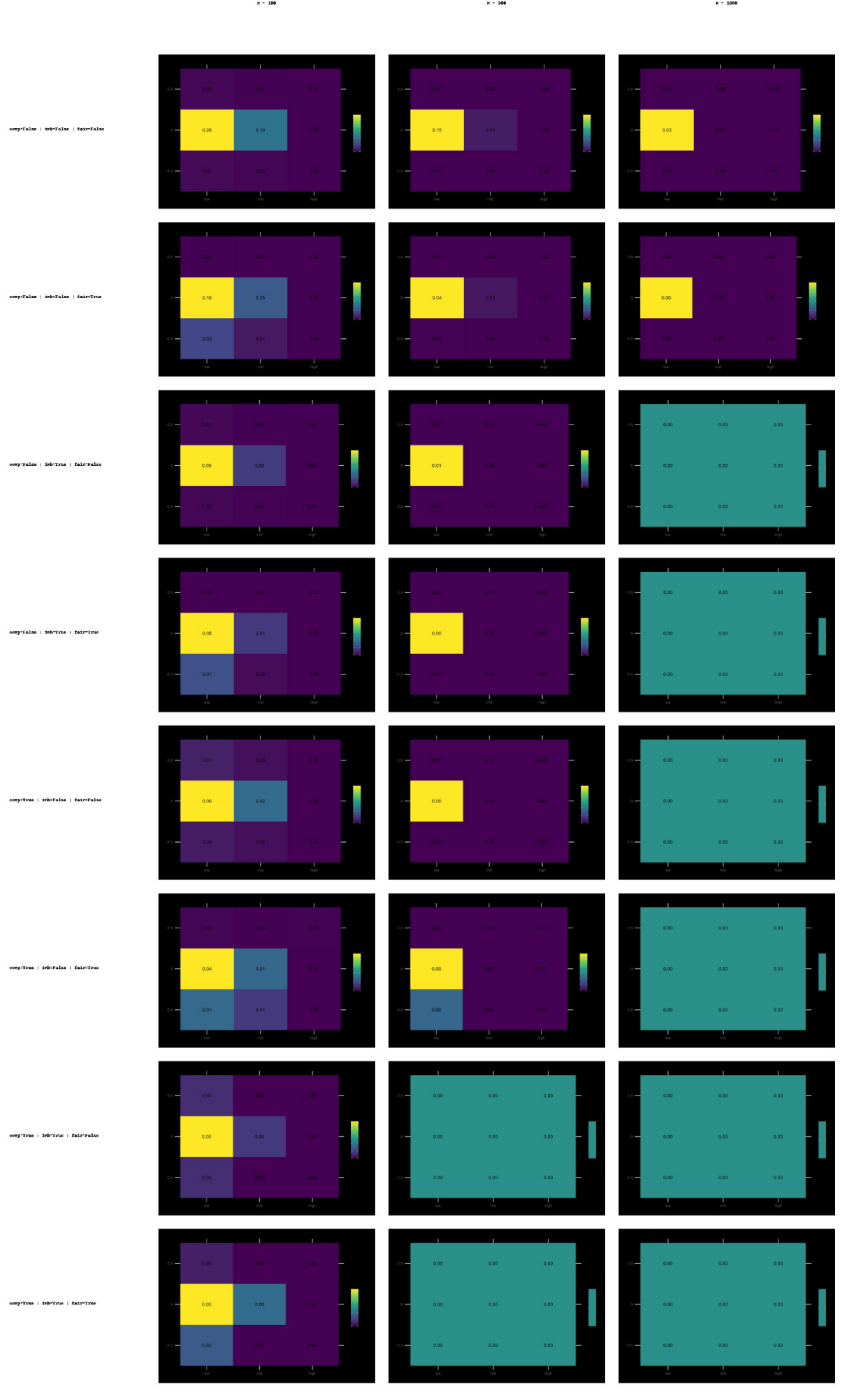
Heat maps of Krippendorff’s α (ordinal) success rate values for different sample sizes (*N* = 100, 300, 1,000) and the conditions comp × imbalance × fairness. Heatmaps of Krippendorff’s α success rate values for sample sizes (100, 300, 1,000) under compression, imbalance, and fairness conditions. Lighter tones show consistently low success rate.

Examining the heatmaps for α, the shading remains predominantly light. Supplementary Figure 2 displays all the Krippendorff’s α heatmaps, which are summarized in Figure 2. This indicates that α rarely reaches the adequacy threshold under any sample size or combination of conditions. This is consistent across all sampling conditions. No panel shows any concentrations of high threshold exceedances.

#### 3.3.4 QWK heat maps

**FIGURE 3:**
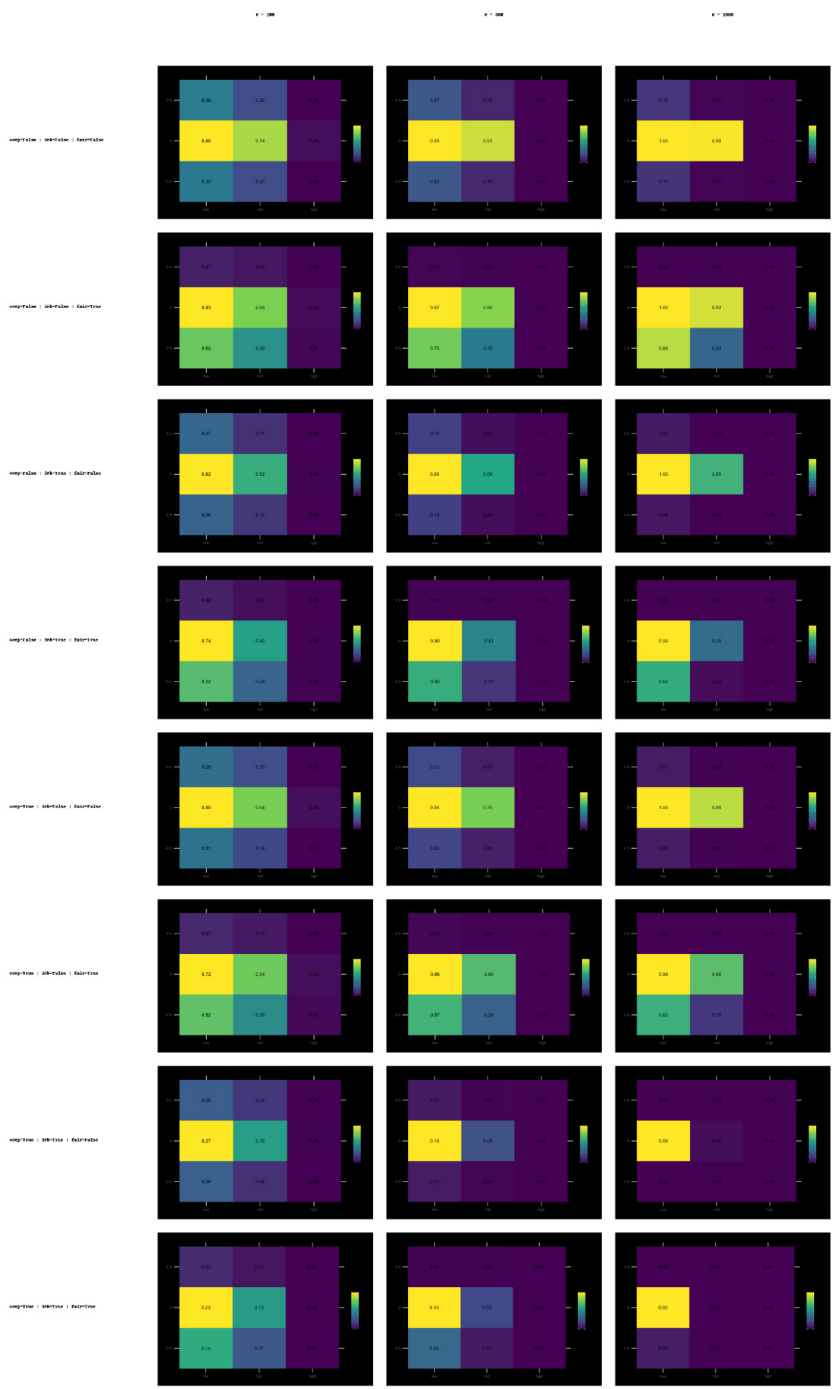
Heat maps of success rate values for quadratic weighted kappa (QWK) for different sample sizes (*N* = 100, 300, 1,000) and the conditions of comp × imbalance × fairness. Heatmaps of QWK success rate values across sample sizes and error conditions. Colors illustrate intermediate sensitivity compared to ICC and Krippendorff’s α.

Heatmaps for QWK values reflect a mix of lighter and darker regions for combinations of conditions. Supplementary Figure 3 includes all QWK heatmaps, with the primary patterns illustrated in Figure 3. For larger sample sizes, some panels contain darker areas indicating higher threshold exceedance success. Area colors become lighter for conditions associated with increased variance or structural changes. The observed changes for the compression, imbalance, and fairness conditions suggest that success rates vary across conditions.

#### 3.3.5 Threshold-exceedance curves across bias and variance conditions

**FIGURE 4:**
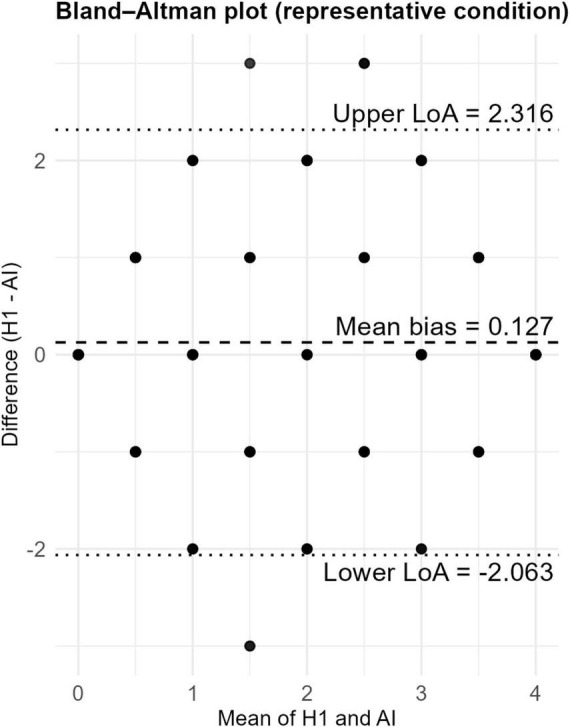
Success rate curves for ICC(A,1), Krippendorff’s α, and QWK metrics, based on bias (–0.5, 0, +0.5) and AI error variance (low, medium, high). Success Rate curves of ICC(A,1), Krippendorff’s α, and QWK across sample sizes (100–1,000), bias levels, and AI error variances. ICC and QWK increase with N, while α remains low.

Figure 4 presents threshold-exceedance curves across sample sizes, bias levels, and variance levels. This subsection summarizes cross-panel patterns rather than repeating panel contents.

#### 3.3.6 Sample size patterns

ICC(A,1) and QWK curves increase monotonically with sample size, reflecting improved stability with larger N. Krippendorff’s α curves remain low regardless of sample size, illustrating persistent conservatism.

#### 3.3.7 Bias effects

Negative and positive bias reduced threshold-exceedance uniformly by shifting human–AI covariance structures. ICC(A,1) and QWK were marginally affected; α showed pronounced deterioration due to additive distortions amplifying disagreement counts.

#### 3.3.8 Variance effects

High variance exerted the strongest negative effect. All metrics approached zero threshold-exceedance under high variance for every bias condition, confirming that multiplicative error dominates all other distortions.

#### 3.3.9 Tolerance-based agreement and Bland–Altman diagnostics

##### 3.3.9.1 Tolerance-based agreement ( ± 1 point)

**FIGURE 5:**
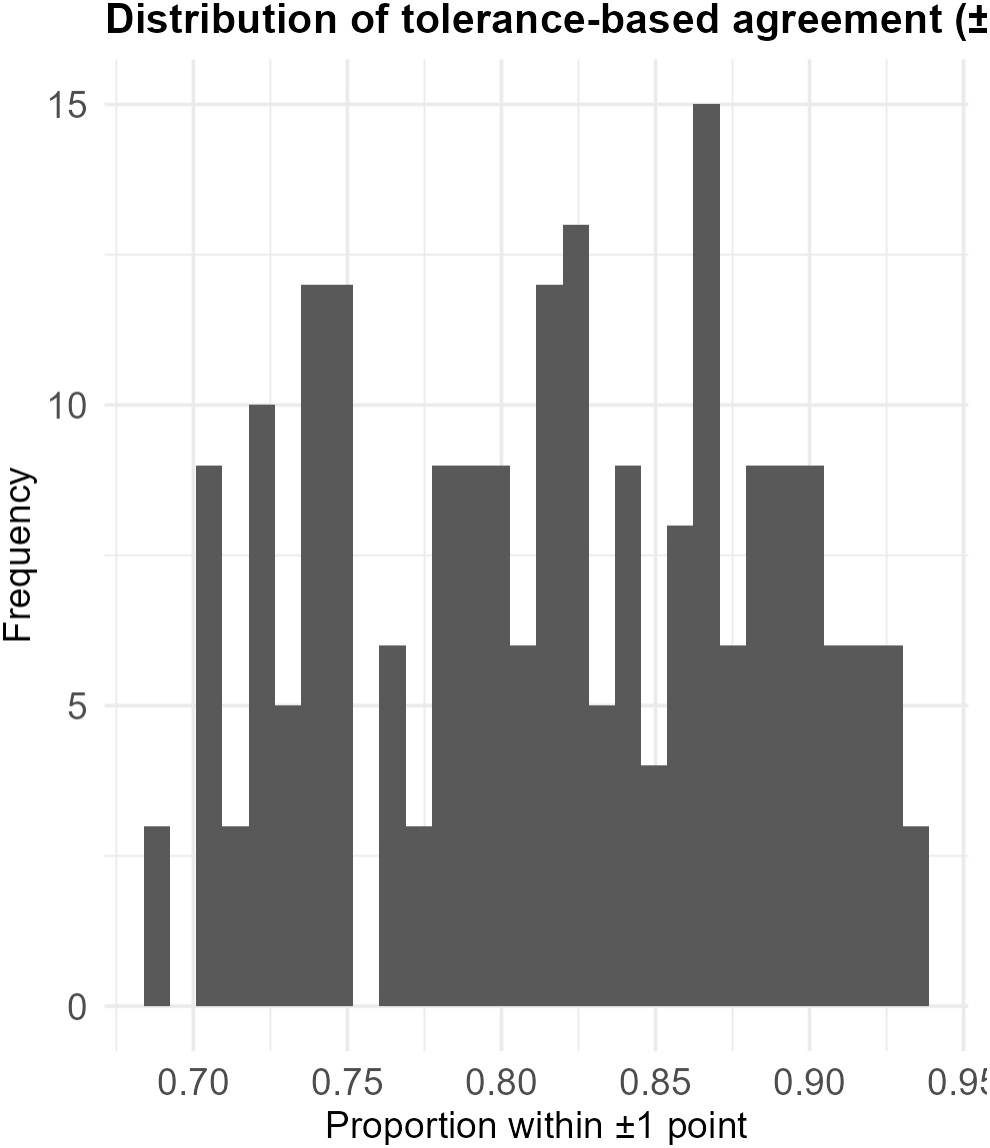
Distribution of success rates falling within ± 1 point of the tolerance-based agreement metric. Histogram showing tolerance-based agreement (±1 point) success rates across simulation conditions. Most values cluster between 0.70 and 0.95.

Tolerance-based agreement exhibited a right-skewed distribution, with most threshold-exceedance proportions between 0.70 and 0.95. This indicates that proximity-based measures remain relatively stable even under substantial variance distortions, providing complementary insight beyond covariance- or distance-based indices. The distribution of these success rates is visualized in Figure 5.

##### 3.3.9.2 Bland–Altman analyses

Bland-Altman plots reveal additional dimensions of fit indices to global fit indices (Supplementary Figure 4 presents all Bland–Altman plots for all sample size condition.):

Sample size effects: The limits of fit narrow as the sample size increases, indicating that random error decreases at higher sample sizes.

Bias effects: Under the –0.5 and +0.5 bias conditions, the mean difference lines were consistently shifted upwards and downwards.

Variance effects: When a higher AI variance was applied, the differences in Bland-Altman plots were observed to spread over a wider range. This indicates a higher frequency of outliers.

Compression effects: Under these conditions, the differences were observed to be concentrated within a narrower range at the extremes.

Imbalance effects: Under these conditions, the difference values were concentrated in certain regions. The bias was observed to be more pronounced in certain ranges.

**FIGURE 6:**
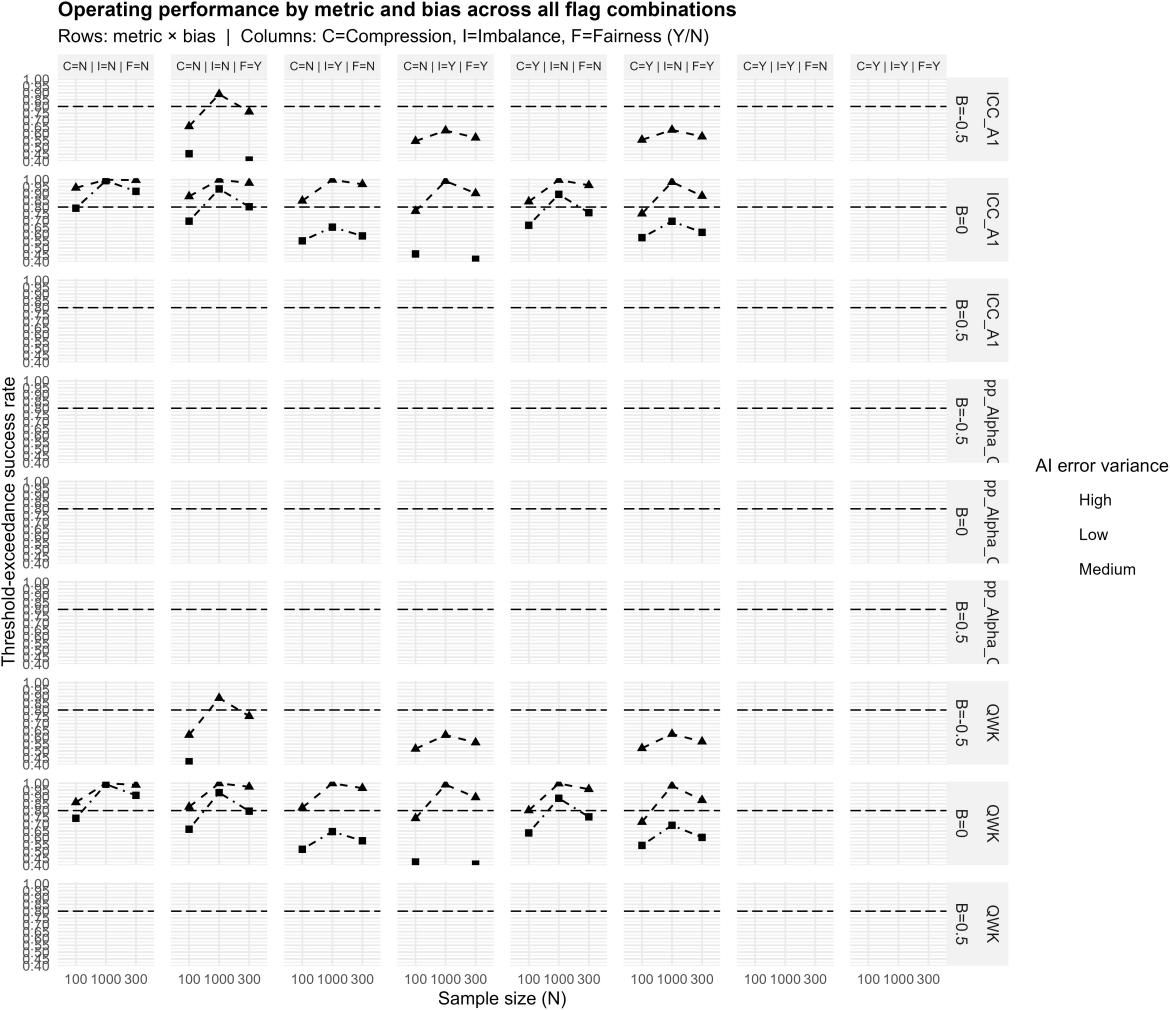
Bland–Altman plot visualizing the differences between human and AI scores for the N = 300, bias = 0, and medium variance conditions. Bland–Altman plot for N = 300, bias = 0, medium variance. X-axis shows mean scores; Y-axis shows human–AI differences; limits of agreement are marked at ± 1.96 SD.

Fairness effects: Under the fairness condition, regular shifts in the mean difference lines occurred between groups, and differences were observed to differ by group. A comprehensive overview of the operating performance across all flag combinations is provided in Figure 6.

##### 3.3.9.3 Real scoring data validation

To provide additional evidence for the results obtained under simulated conditions, a validation analysis of real-score data was conducted using the writing texts of six B2-level students. For each trial, all ratings were performed by three human raters, each with varying degrees of familiarity with the students, and two major language model-based automated scoring systems (ChatGPT and Gemini). A new variable (HumanRef) was calculated as the arithmetic mean of the human raters’ ratings. To provide a single human reference point for comparison, a reference score (HumanRef) was calculated as the average of three teacher ratings for each observation. Human-rating variability was quantified by calculating the residuals (Teacher—HumanRef) for all teachers and estimating the pooled standard deviation of these residuals.

For each AI system, point estimates of the bias and variance distortion (AI—HumanRef) were derived from the distribution. Mean differences were expressed both as raw score units and as a percentage of the full 0–70 scale. AI error variability was characterized by the standard deviation (AI—HumanRef) and compared to the human error standard deviation to obtain the AI-to-human variance ratio. This ratio was then divided into low, medium, or high variance levels according to predetermined thresholds. AI variance values were matched to the nearest Monte Carlo condition (-0.5, 0, +0.5).

Bootstrapping estimates indicate that both AI systems exhibit negative mean deviations relative to the human reference. GPT exhibits a deviation of0 −9.00 points, while Gemini exhibits a larger negative deviation of −12.05 points. In both cases, the corresponding 95% confidence intervals remain below zero. The two systems differ in their variance ratios. A lower relative variance (0.84) is calculated for GPT, while Gemini’s ratio (1.24) exceeds the human variance estimate. The slope values obtained from the AI-HumanRef regressions fall within the subunit range; GPT yields an estimate of 0.50, while Gemini yields an estimate of 0.67.

Compression probabilities are high for both AI systems. GPT is higher for both systems. Fairness probabilities are close to 1.00 for both systems. In the matches made with the simulated conditions, the GPT’s medium variance condition was found to be consistent with the Gemini’s high variance condition. Both systems had a bias condition of −0.5. The compression and fairness conditions were marked as “true.”

The results from the analyses indicate significant convergence between empirical AI-human discrepancies and the simulated variance conditions. The negative bias, compression, and high fairness violation probabilities observed in real scoring are consistent with the simulated conditions. This similarity provides strong external validation for the simulation model. The variance manipulations generated in the simulation confirm that they reflect realistic models of AI-based scoring.

## 4 Discussion

This section examines the results obtained under simulated conditions and real data.

### 4.1 Effects on performance under null conditions and threshold-exceedance performances

A key aspect of this study is that it compares both null-condition performance and threshold-exceeding performance under simulated conditions with results obtained from real data. ICC(A,1) and QWK showed high threshold exceedances in the null scenario. This result demonstrates that the conventional adequacy limits of these metrics can be easily exceeded even in the absence of a true discrepancy. In contrast, Krippendorff’s α produced almost no false threshold-exceedances. This finding indicates that α is a significantly more consistent metric under perfect agreement and is consistent with previous evaluations in the literature (Hayes and Krippendorff, 2007; Debelak and Ziegler, 2025).

These distinctions are fundamental to the proper interpretation of subsequent threshold-exceedance results. The findings clearly demonstrate that the three metrics do not measure the same construct. While ICC(A,1) and QWK are sensitive to the shared variance or weighted covariance structure among the metrics, Krippendorff’s α produces a measure of disagreement adjusted for the expected disagreement under marginal distributions (Hayes and Krippendorff, 2007). Therefore, the threshold-exceedance performances of the metrics cannot be directly compared without considering the null behavior. Considering all the results in Tables 3, 4, 7 together provides a more holistic and theoretically consistent interpretation than any single metric could provide.

**TABLE 7:** Threshold-exceedance performance.

Metric	Threshold	Mean_Success	Sd_success
ICC_A1	0.600	0.627	0.408
ICC_A1	0.650	0.432	0.401
ICC_A1	0.700	0.211	0.316
ICC_A1	0.750	0.061	0.152
ICC_A1	0.800	0.006	0.028
Kripp_Alpha_Ord	0.700	0.209	0.310
Kripp_Alpha_Ord	0.750	0.059	0.149
Kripp_Alpha_Ord	0.800	0.006	0.026
Kripp_Alpha_Ord	0.850	0.000	0.001
Kripp_Alpha_Ord	0.900	0.000	0.000
QWK	0.600	0.443	0.404
QWK	0.650	0.387	0.395
QWK	0.700	0.203	0.311
QWK	0.750	0.058	0.146
QWK	0.800	0.005	0.024

### 4.2 Mechanistic interpretation of metric behavior

#### 4.2.1 Additive mechanism (bias)

Additive distortions across conditions consistently decreased threshold-exceedance performance. Bias produced a monotonic change in AI scores, weakening the covariance structure even when some rank similarity was maintained. For ICC(A,1) and QWK, this weakening led to proportional decreases, reflecting their dependence on shared variance and weighted covariance. However, Krippendorff’s α showed a more pronounced decrease under additive distortion conditions, indicating that there is a difference between the discrepancy-based formulation of α and the covariance-based structure of ICC and QWK.

#### 4.2.2 Variance inflation

Variance inflation has the strongest effect across all conditions. When the AI score distribution exceeded the variability of human scores, threshold-exceedance performance dropped to nearly zero for all metrics. This result is consistent with classical psychometric principles: inflation weakens covariance-based indices (ICC, QWK), while distance-based discrepancy metrics like α inflate, reducing reliability. This additional distortion effect is more pronounced for α. This can be attributed to the metric’s dependence on marginal category distributions and squared distances.

#### 4.2.3 Distributional distortions (compression and imbalance)

Compression, where AI systems avoid extreme scores, degraded the performance of all metrics. For ICC(A,1) and QWK, compression reduced the variance in AI scores compared to the reference score distribution and therefore weakened the covariance. For α, compression created regions of high disagreement in marginal cells. These findings are consistent with previous studies on artificial agreement in score distributions exhibiting centrality biases (Yiğiter and Boduroğlu, 2025). The imbalance (skewed prevalence) condition was observed to have similarly negative effects, particularly for α. This is thought to be due to the unequal marginal distributions distorting the expected disagreement matrices. This effect was less pronounced for ICC(A,1) and QWK, but significant decreases in threshold-exceedance performance were still observed. This suggests that these two metrics are sensitive to distributional imbalances.

### 4.3 Metric-specific interpretations and practical implications

#### 4.3.1 ICC(A,1): stable but liberal

ICC(A,1) exhibited strong stability across sample sizes and moderate distortions, confirming its established role as a reliable measure of interrater consistency (Koo and Li, 2016). However, its high null liberality suggests that while ICC(A,1) is effective at detecting correspondence between rating patterns, it may overstate adequacy in contexts where thresholds (≥0.70) are applied without accounting for null behavior. ICC(A,1) is therefore best used when stability and variance homogeneity can be reasonably assumed.

#### 4.3.2 Krippendorff’s α (ordinal): conservative and distribution-sensitive

Krippendorff’s α displayed the lowest operational adequacy across all conditions, consistent with findings that α is highly sensitive to small sample sizes, marginal asymmetries, and score imbalances (Hayes and Krippendorff, 2007). Its near-zero threshold-exceedance values under even moderate variance distortions indicate that α should not be interpreted as a direct analog to ICC(A,1) or QWK in operational adequacy contexts. Instead, α should be regarded as a strict diagnostic tool capable of identifying even minor deviations from expected agreement.

#### 4.3.3 Quadratic weighted kappa: intermediate robustness

QWK demonstrated moderate sensitivity to both variance and bias, situating it between ICC(A,1) and α. QWK’s squared-distance weighting provides advantages when rating scales are ordinal and misclassifications near category boundaries should be penalized (Quah et al., 2024). However, this metric is moderately susceptible to variance inflation. This suggests that AI scores should be interpreted with caution, particularly for conditions with high error variance or unbalanced distribution.

### 4.4 Fairness, subgroup bias, and measurement equivalence

Fairness is a fundamental dimension in automated scoring. The findings from this study are consistent with studies on fairness (American Educational Research Association [AERA] et al., 2014; Dorans and Liu, 2009; Ercikan and McCaffrey, 2022). ICC(A,1) and QWK were found to have high threshold exceedance performance even when systematic subgroup biases were introduced. This revealed that these metrics have serious limitations in detecting group-level inequalities.

In the real dataset, both GPT and Gemini tended to emphasize certain subgroups. In other words, they produced high probabilities of a fairness violation. The results obtained from the simulation data are consistent with those obtained from the real data. This consistency suggests that the subgroup distortions seen in real AI scoring can be explained by the variance inflations used in the simulation. All these findings suggest the need to include additional Bland-Altman-based methods in validity studies for automated scoring (Williamson et al., 2012; Johnson and McCaffrey, 2023; Schaller et al., 2024).

### 4.5 Integration of simulation findings with empirical data

Results from real data indicate that negative bias, compression, and subgroup offsets occur in the real situation. This corresponds to the simulation conditions of N = 100, bias = -0.5, medium to high variance, compression = true, imbalance = false, and fairness = true. The empirical observation that Gemini and GPT produce both narrower variance cases and subgroup-specific imbalances further demonstrates the importance of combining metrics with different diagnostic tools (regression slopes, variance ratios, Bland-Altman plots, etc.). The presence of a consistent negative bias and observable variance distortions is consistent with previous studies showing that automated scoring systems tend to deviate from human raters due to the possibility of systematic underestimation and reduced score distribution (Dadi and Sanampudi, 2021). Similarly, the observed group-based fairness effects (particularly those associated with rater familiarity) are consistent with other results suggesting that contextual or demographic factors can influence both human and automated scoring outcomes (Johnson and McCaffrey, 2023). Although some simulated parameters (e.g., variance magnitude) were not captured at the same levels, the overall agreement between the bias, compression, and fairness dimensions provided strong external validity for the simulation framework. This empirical evidence reinforces the convergent validity between simulated and real data models.

### 4.6 Threshold-exceedance limits and decision framework for practitioners

Based on all the results obtained regarding concordance metrics, the following recommendations are made for researchers:

1. In cases where variance distortions are likely: ICC(A,1) and Bland-Altman analysis should be preferred together.
2. In cases where distributional irregularities or spread are likely: QWK may be preferred. However, weighted patterns of discordance should definitely be examined.
3. Krippendorff’s α is the most sensitive metric for cases with small disaggregations.
4. For fairness conditions: No single metric is sufficient. Additional subgroup-level analyses should be conducted.
5. All of these are consistent with studies emphasizing multi-metric approaches to AI scoring validity (Schaller et al., 2024).

### 4.7 Limitations and directions for future research

This study’s simulation design used the assumption that human rater variance is constant. While this approach is necessary to isolate the simulation conditions, it does not fully reflect the true scoring variability of human raters (Eckes, 2015; Myford and Wolfe, 2003, 2004). Future simulations could also incorporate variation in human rater variance and its properties, such as tolerance, centrality, and halo effects. Furthermore, the fairness offsets used in this study are more limited than the approximately 15-point variance observed in empirical analyses. This suggests that real-world fairness problems may be more severe than those addressed in simulations.

### 4.8 Practical implications and recommendations

The simulation results demonstrate that the operational performance of agreement metrics is highly contingent upon the underlying error structure and sampling conditions. Accordingly, the present study supports a multi-metric, condition-specific evaluation framework for AI-based scoring systems rather than relying on a single coefficient. Based on the observed performance patterns across 216 simulation conditions, the following practical recommendations are proposed:

#### 4.8.1 Systematic bias

Across bias levels of ± 0.5, Bland–Altman plots and tolerance-based overlap indices revealed directional mean shifts that correlation-based indices often masked. ICC(A,1) and QWK tended to maintain high overall threshold-exceedance proportions (≈0.80–0.90) even when the mean offset exceeded one score point, whereas Bland–Altman analyses effectively visualized the bias direction and 95% limits of agreement.

In applications where consistent over- or under-scoring patterns may emerge between human and AI raters, Bland–Altman analysis and tolerance-based indices should be prioritized (Giavarina, 2015; Quah et al., 2024).

#### 4.8.2 Construct-irrelevant variance (error variance manipulations)

When AI error variance increased beyond the human reference level (σ^2^ × 1.5–3), ICC(A,1) exhibited the lowest decline in threshold-exceedance proportion (-8%), indicating high robustness to random noise. In contrast, QWK dropped by up to 25 %.

For conditions with elevated random error, ICC(A,1) remains the most stable indicator of scoring consistency (Koo and Li, 2016; Liljequist et al., 2019). For conditions where random error was high, ICC(A,1) showed relatively modest decreases in threshold-exceedance performance. This is consistent with previous descriptions of its behavior under increased score variability (Koo and Li, 2016; Liljequist et al., 2019).

#### 4.8.3 Mid-score compression

Under compression conditions where AI scores were around the midpoint, ICC(A,1) and α showed overestimated fit values (more than 10% false positives). On the other hand, QWK maintained threshold-exceedance performance by distinguishing partial agreement from full agreement. When scores are concentrated within a narrow range or values are compressed toward the midpoint, QWK reveals this more readily than other metrics because its weighting structure is particularly sensitive to such compression (Uto, 2021).

#### 4.8.4 Class imbalance

Krippendorff’s α decreased significantly in unbalanced score distributions, while QWK showed a more limited change. Therefore, α should not be interpreted alone under skewed distributions; it should be used in conjunction with QWK or tolerance-based measures (Hayes and Krippendorff, 2007).

#### 4.8.5 Group-based bias (fairness condition)

In fairness simulations, the ICC and QWK metrics can mask small but systematic deviations across subgroups. In contrast, Bland–Altman and tolerance-based analyses have shown such shifts more clearly. Therefore, fairness assessment requires the use of these metrics in conjunction with subgroup residual analyses, in accordance with principles of measurement fairness (Ercikan and McCaffrey, 2022; Johnson and McCaffrey, 2023).

#### 4.8.6 Sample size effects

Simulations have shown that small samples exhibit higher variability across all metrics, but that larger samples yield more consistent results. The results confirm that ICC(A,1), in particular, provides more consistent inferences in small samples compared to tolerance-based approaches (Shrout and Fleiss, 1979; Koo and Li, 2016). In general, each metric captures a different aspect of fit:

- ICC(A,1) for overall consistency,
- QWK for ordinal threshold-exceedance performance,
- Krippendorff’s α for conservative lower-bound estimation, and
- Bland–Altman/tolerance-based methods for bias and fairness diagnostics.

Therefore, in human-AI scoring comparisons, interpreting the metrics together and in a complementary manner would be psychometrically stronger than relying on a single measure.

## 5 Conclusion

This study demonstrates how AI-based scoring varies under different error structures, demonstrating that agreement metrics are both context-sensitive and complementary. The findings suggest that assessing patterns solely with a single index may be insufficient and that validity and fairness analyses should be considered together. In this context, we believe that a multi-metric approach (e.g., ICC, QWK, Krippendorff’s α, as well as Bland–Altman and tolerance-based methods) will provide a more reliable and fair interpretation of human–AI scoring comparisons.

## Data Availability

The datasets presented in this study can be found in online repositories. The names of the repository/repositories and accession number(s) can be found at: 10.5281/zenodo.17119842.
