# Supplementary figures and images for: Operating characteristics of agreement metrics in AI-based scoring: a Monte Carlo simulation (part 3 of 3)
Source: Front Psychol. 2026 Feb 19;17:1705653. doi: 10.3389/fpsyg.2026.1705653 (PMC12960511; doi:10.3389/fpsyg.2026.1705653)

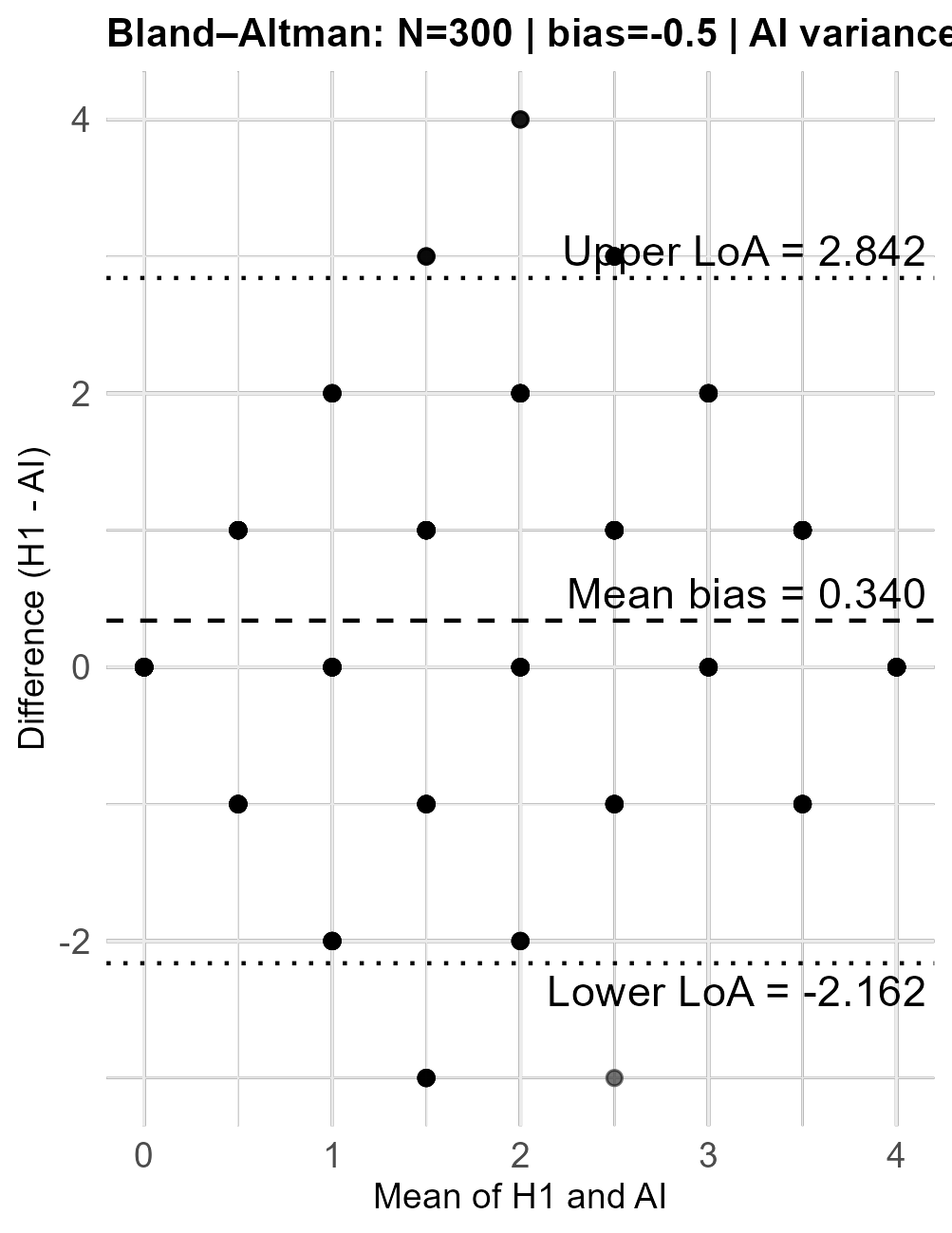

Supplement: Supplementary file 4 [file Data_Sheet_4.zip › BA_N300_bias-0.5_aihigh_compTRUE_imbFALSE_fairTRUE_rep1.tif]

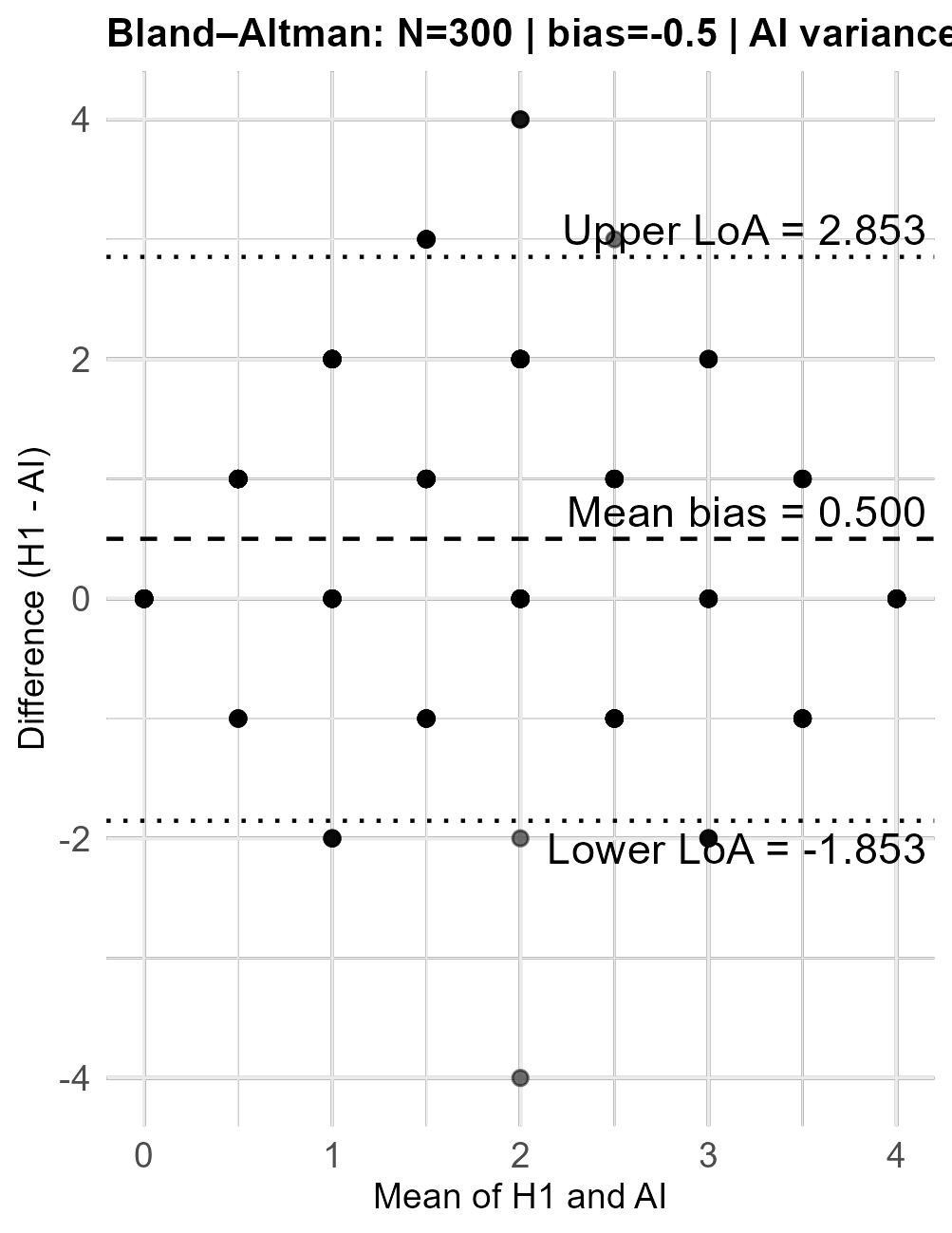

Supplement: Supplementary file 4 [file Data_Sheet_4.zip › BA_N300_bias-0.5_aihigh_compFALSE_imbTRUE_fairFALSE_rep1.tif]

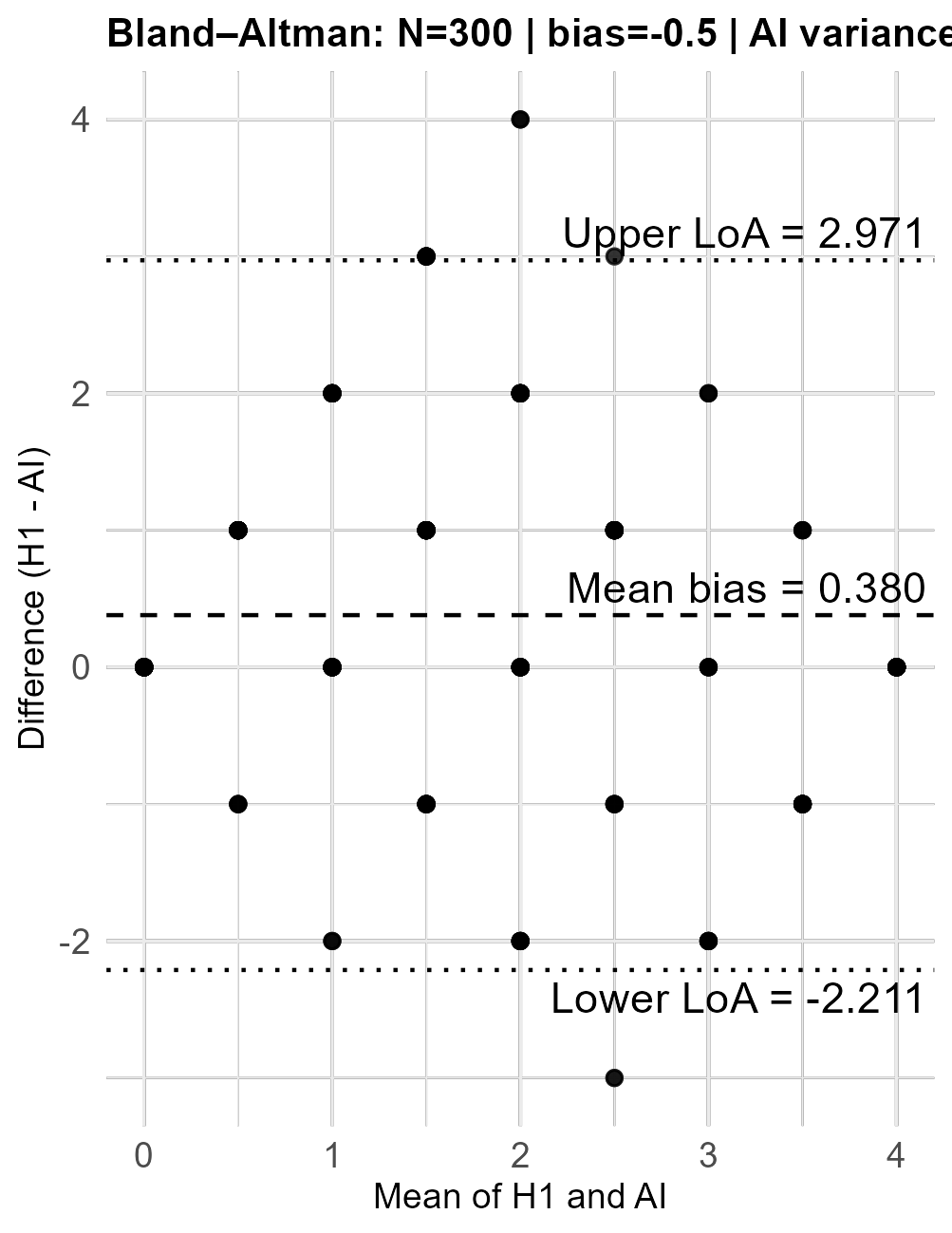

Supplement: Supplementary file 4 [file Data_Sheet_4.zip › BA_N300_bias-0.5_aihigh_compFALSE_imbTRUE_fairTRUE_rep1.tif]

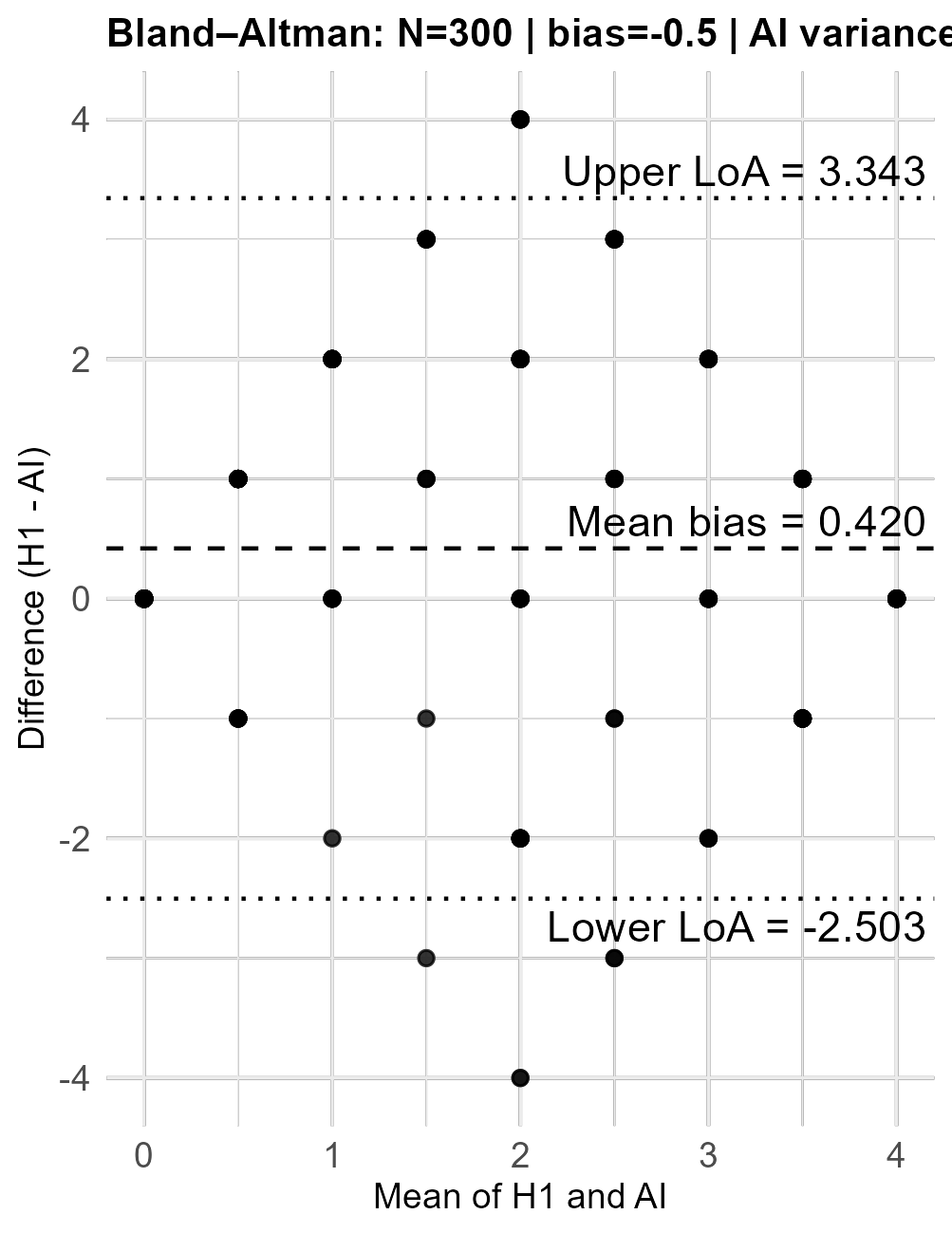

Supplement: Supplementary file 4 [file Data_Sheet_4.zip › BA_N300_bias-0.5_aihigh_compFALSE_imbFALSE_fairFALSE_rep1.tif]

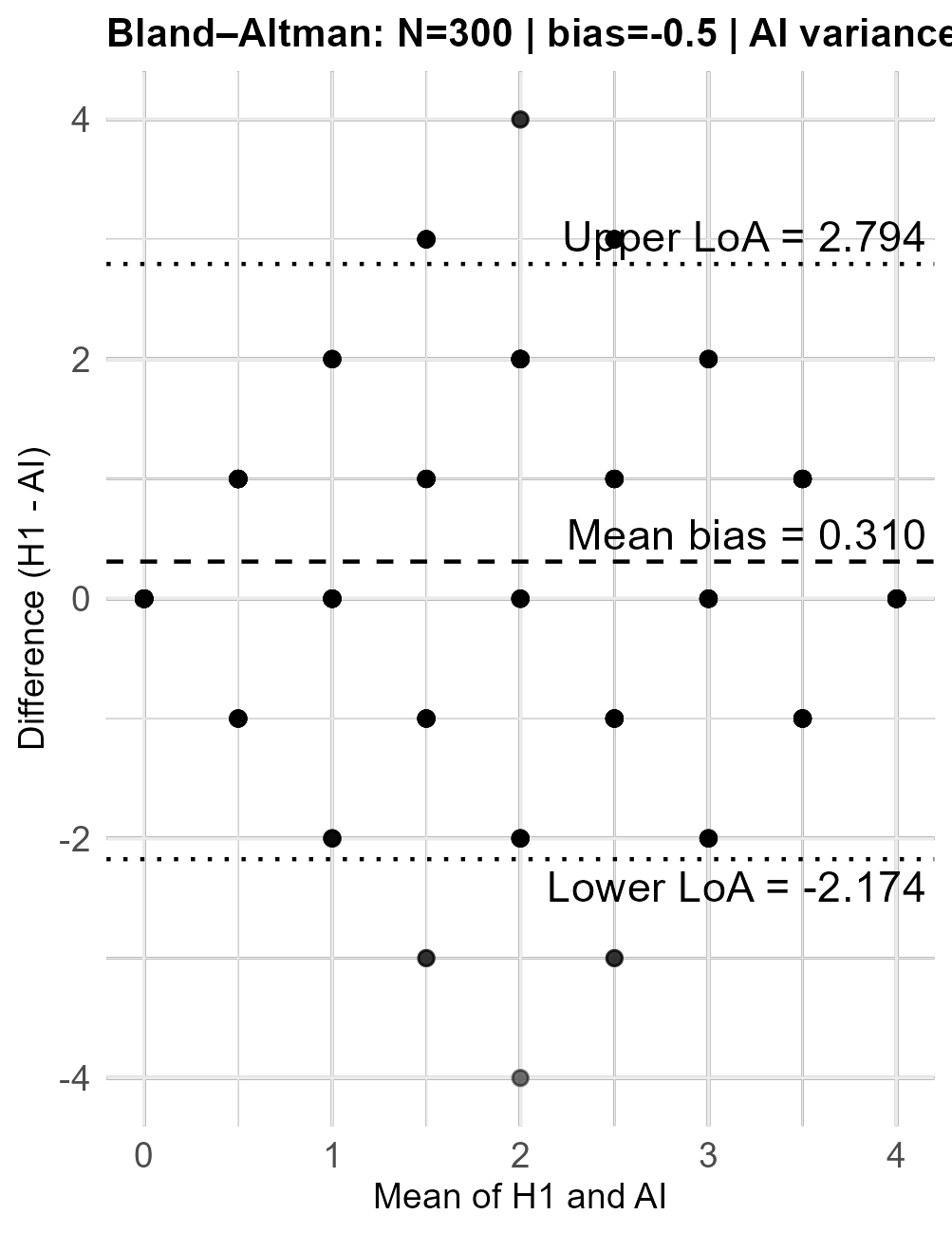

Supplement: Supplementary file 4 [file Data_Sheet_4.zip › BA_N300_bias-0.5_aihigh_compFALSE_imbFALSE_fairTRUE_rep1.tif]

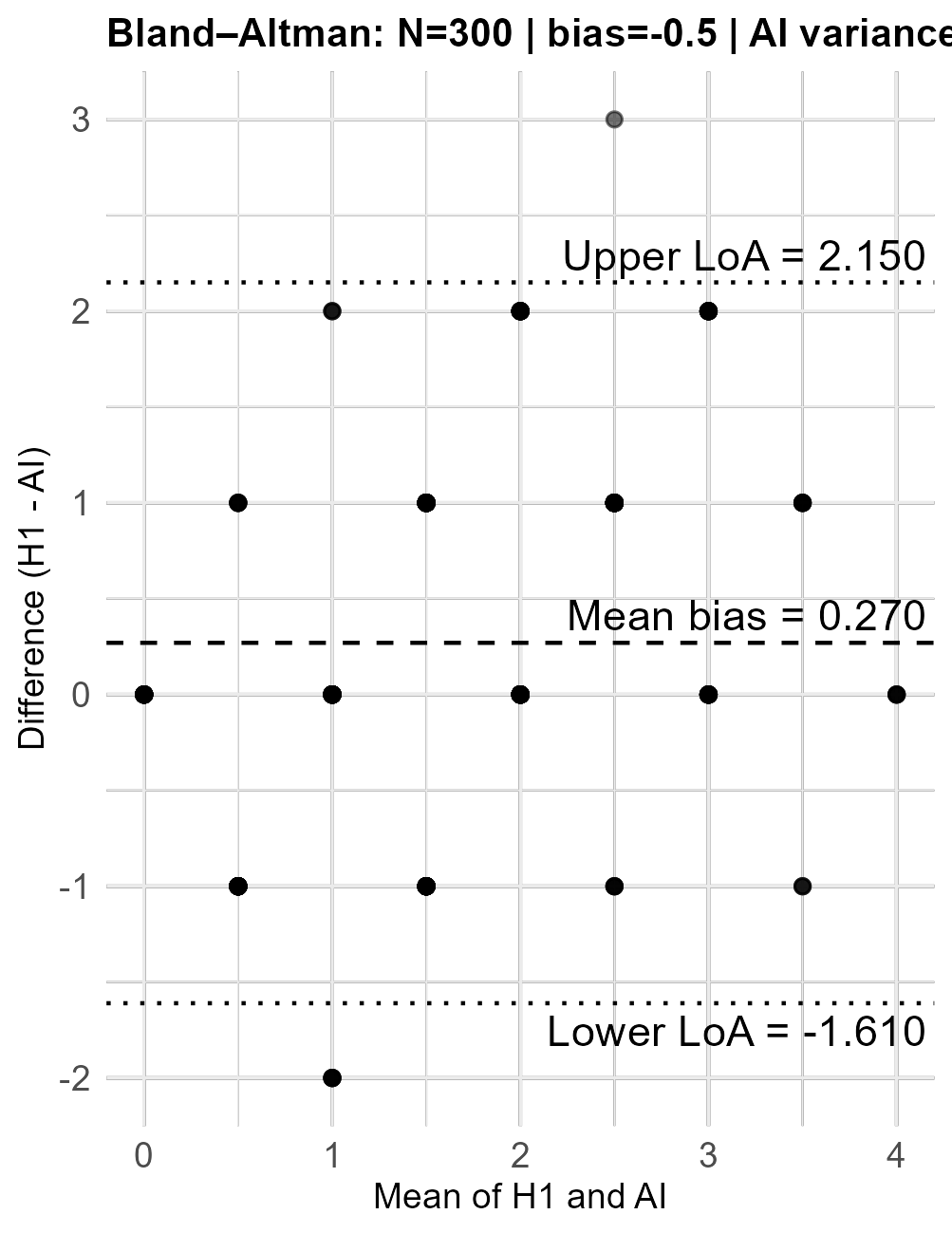

Supplement: Supplementary file 4 [file Data_Sheet_4.zip › BA_N300_bias-0.5_aimid_compTRUE_imbTRUE_fairFALSE_rep1.tif]

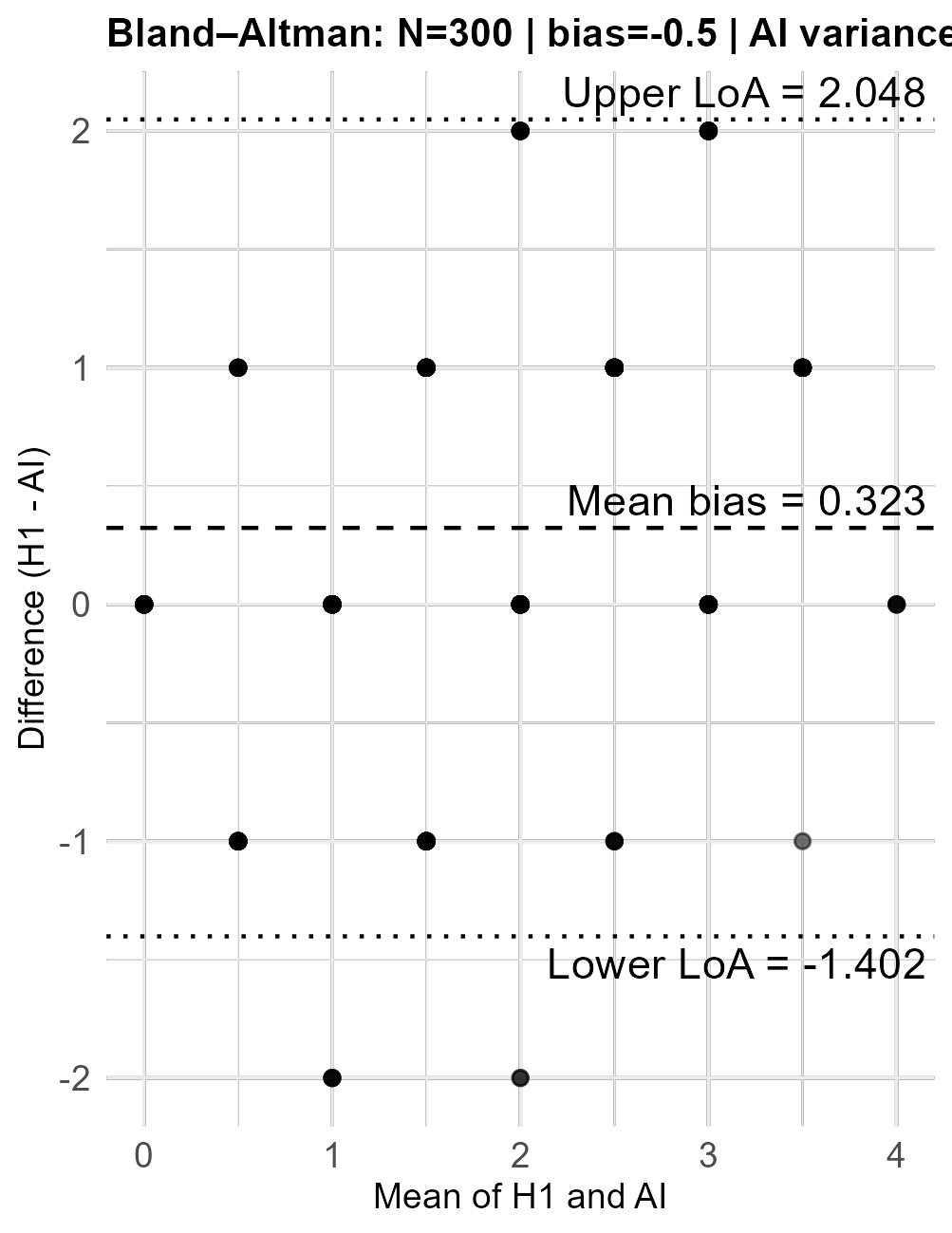

Supplement: Supplementary file 4 [file Data_Sheet_4.zip › BA_N300_bias-0.5_aimid_compTRUE_imbTRUE_fairTRUE_rep1.tif]

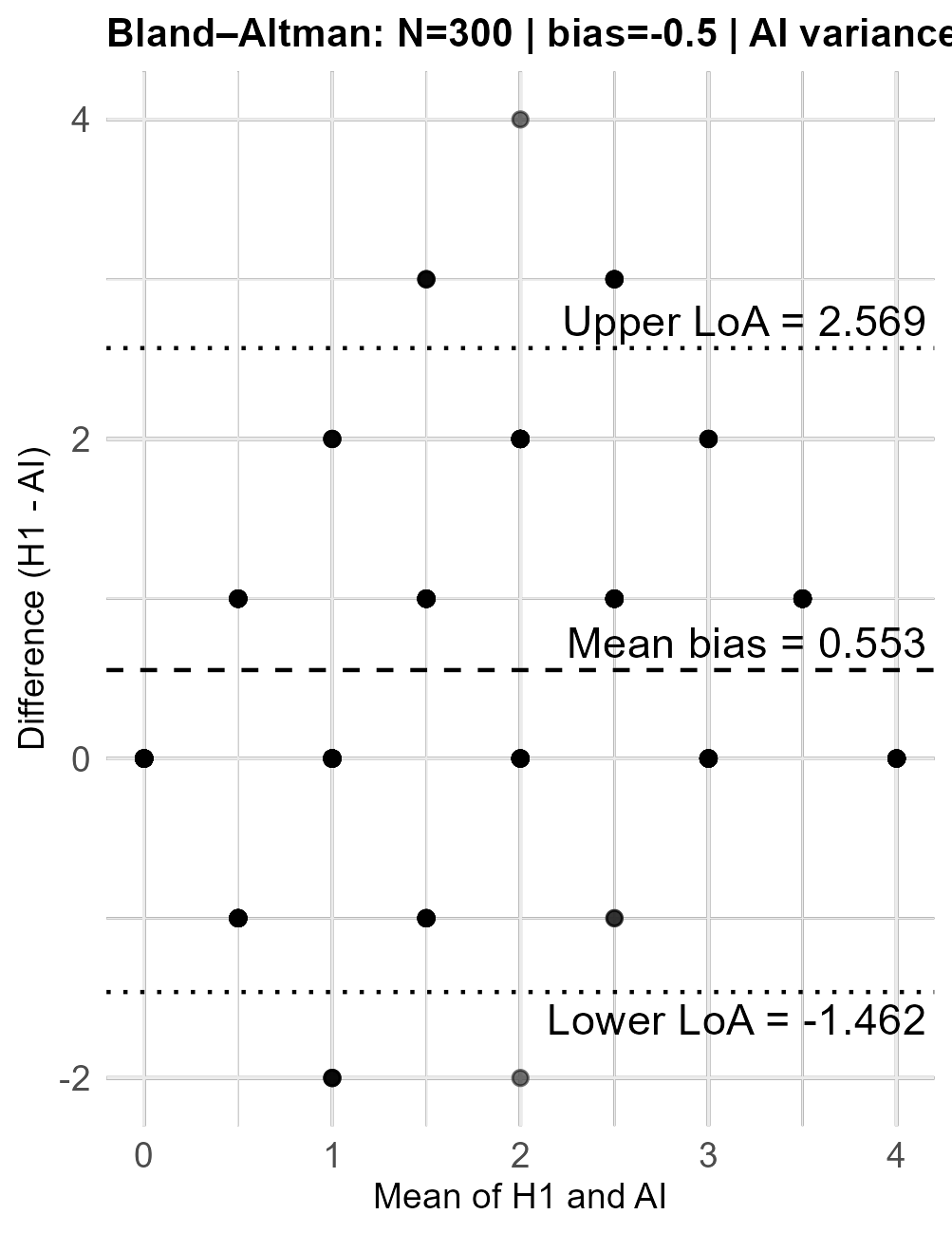

Supplement: Supplementary file 4 [file Data_Sheet_4.zip › BA_N300_bias-0.5_aimid_compTRUE_imbFALSE_fairFALSE_rep1.tif]

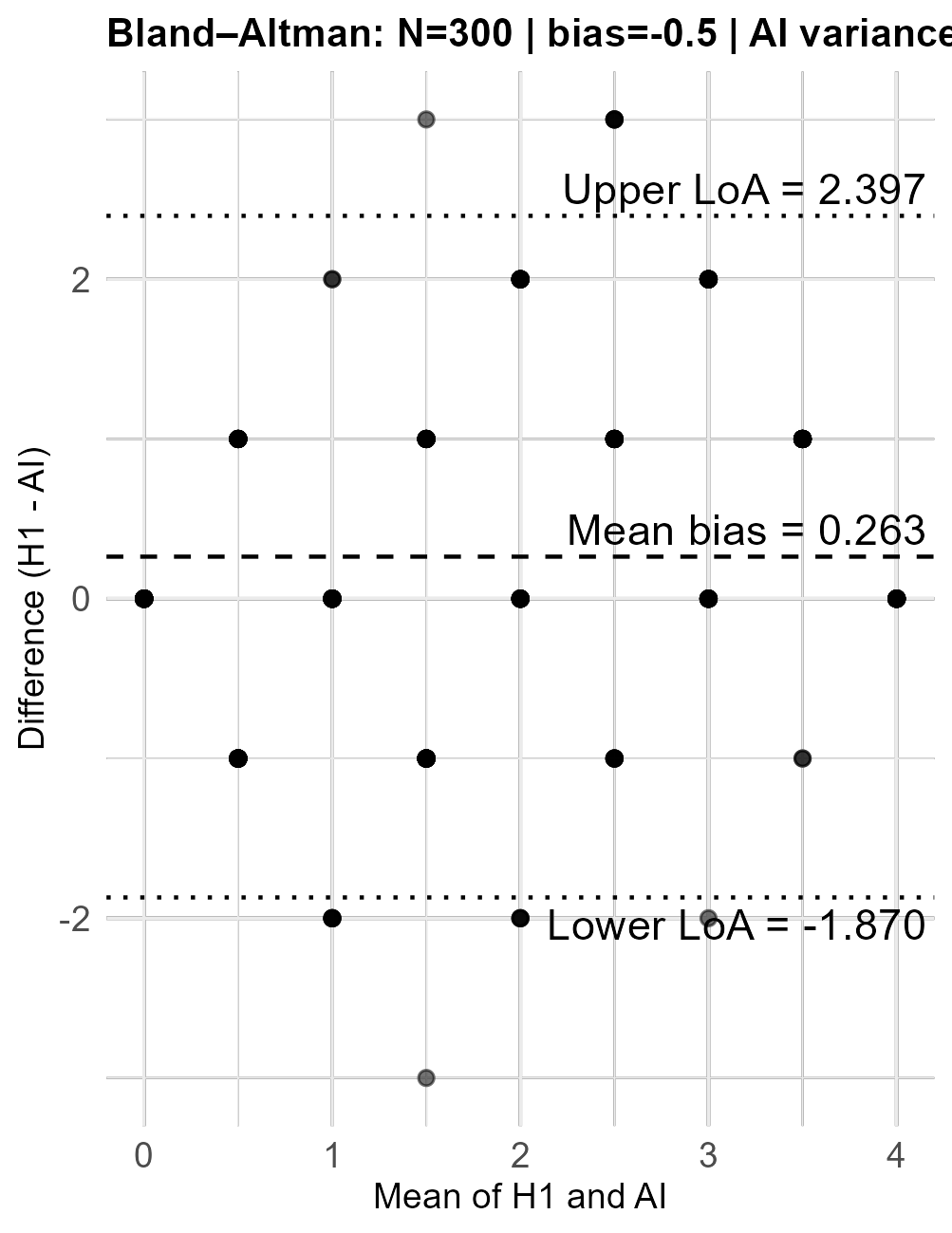

Supplement: Supplementary file 4 [file Data_Sheet_4.zip › BA_N300_bias-0.5_aimid_compTRUE_imbFALSE_fairTRUE_rep1.tif]

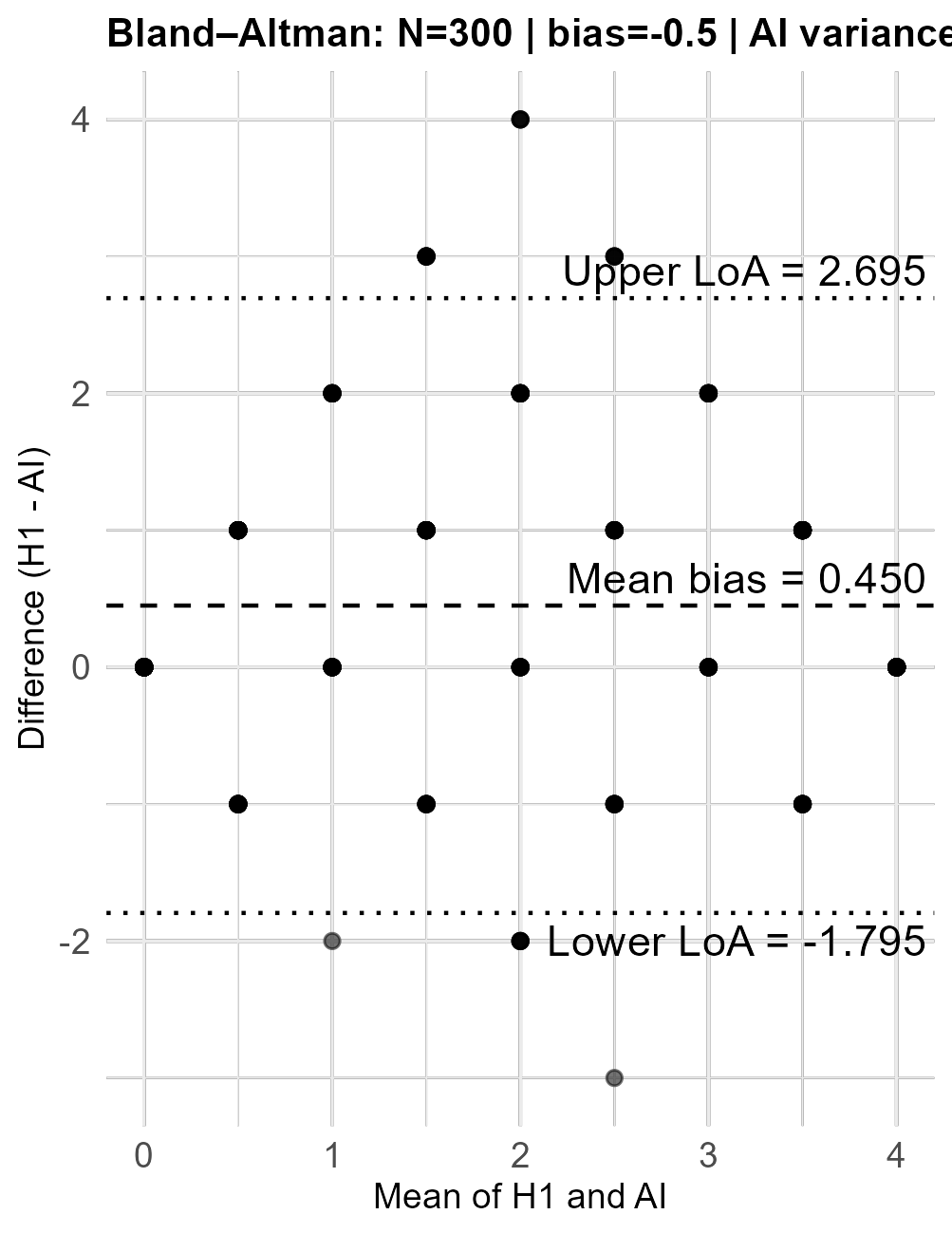

Supplement: Supplementary file 4 [file Data_Sheet_4.zip › BA_N300_bias-0.5_aimid_compFALSE_imbFALSE_fairTRUE_rep1.tif]

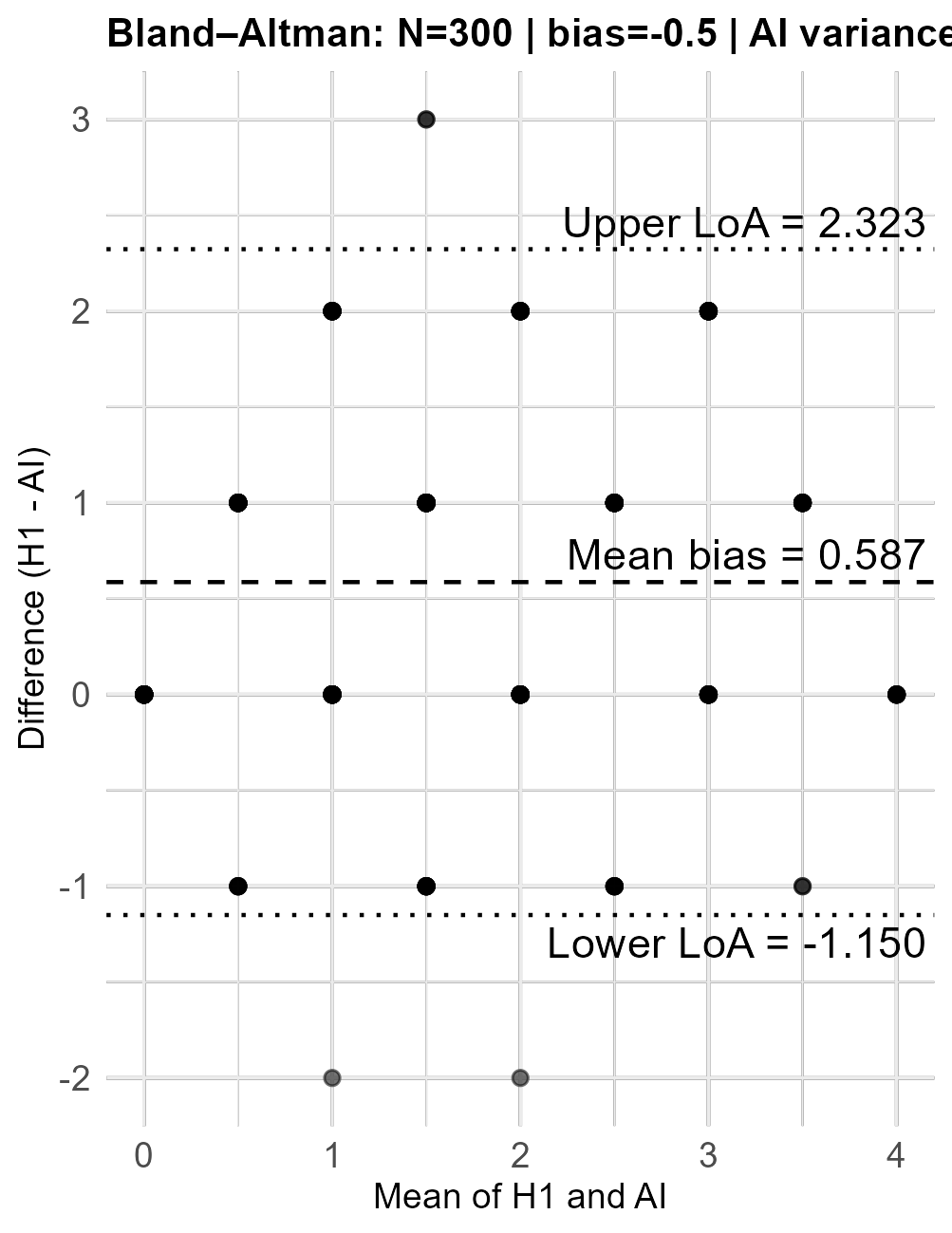

Supplement: Supplementary file 4 [file Data_Sheet_4.zip › BA_N300_bias-0.5_aimid_compFALSE_imbTRUE_fairFALSE_rep1.tif]

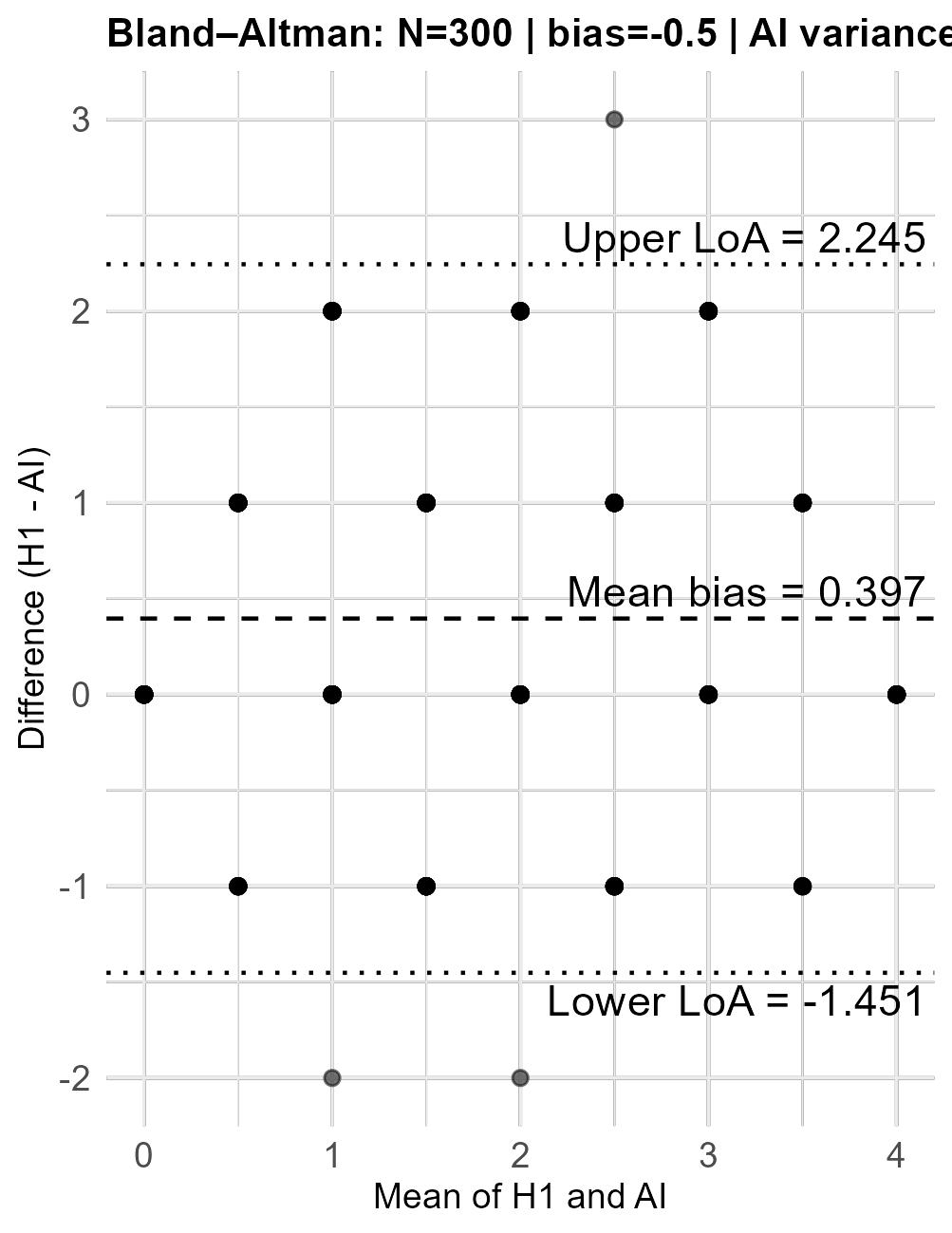

Supplement: Supplementary file 4 [file Data_Sheet_4.zip › BA_N300_bias-0.5_aimid_compFALSE_imbTRUE_fairTRUE_rep1.tif]

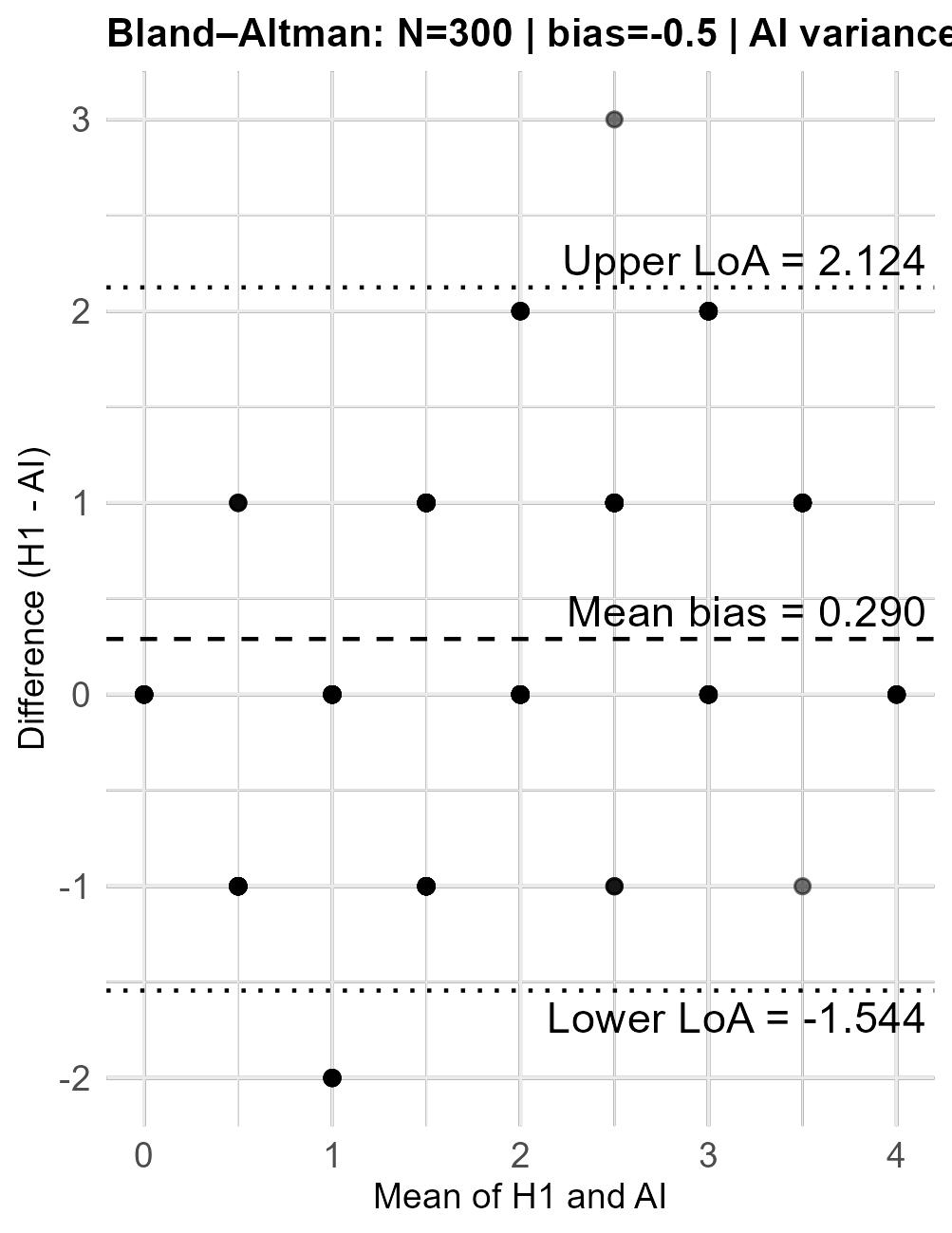

Supplement: Supplementary file 4 [file Data_Sheet_4.zip › BA_N300_bias-0.5_ailow_compTRUE_imbTRUE_fairTRUE_rep1.tif]

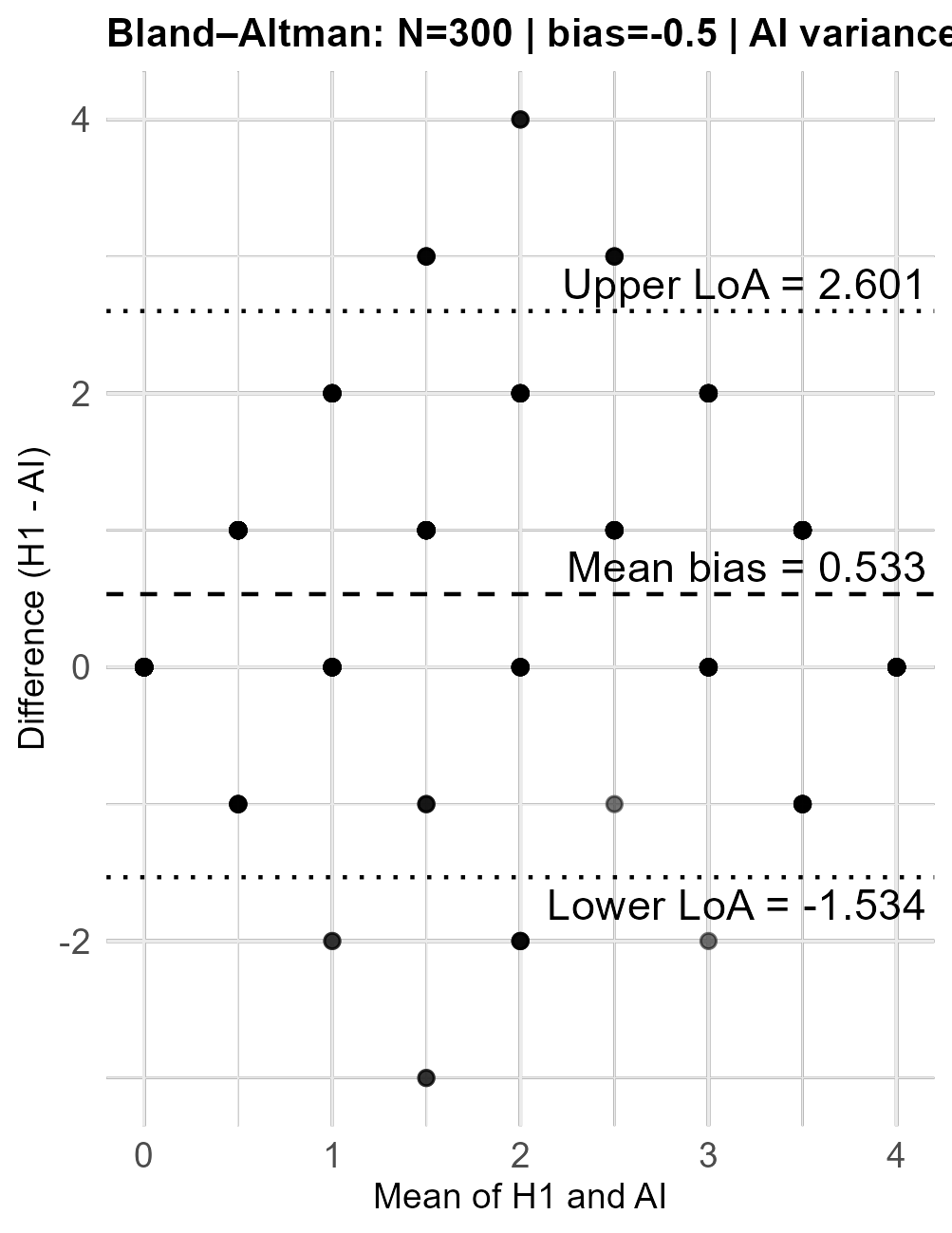

Supplement: Supplementary file 4 [file Data_Sheet_4.zip › BA_N300_bias-0.5_aimid_compFALSE_imbFALSE_fairFALSE_rep1.tif]

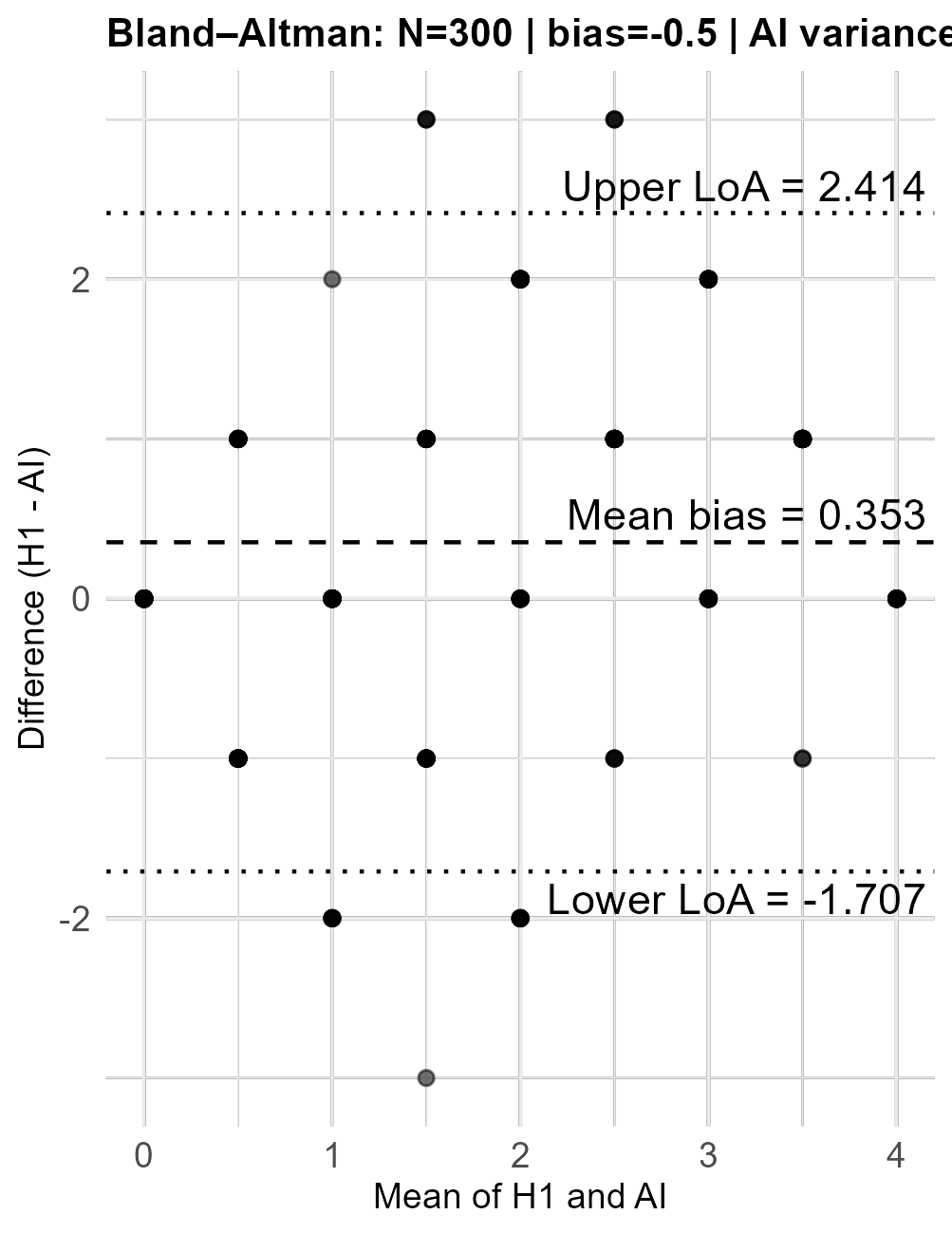

Supplement: Supplementary file 4 [file Data_Sheet_4.zip › BA_N300_bias-0.5_ailow_compTRUE_imbFALSE_fairTRUE_rep1.tif]

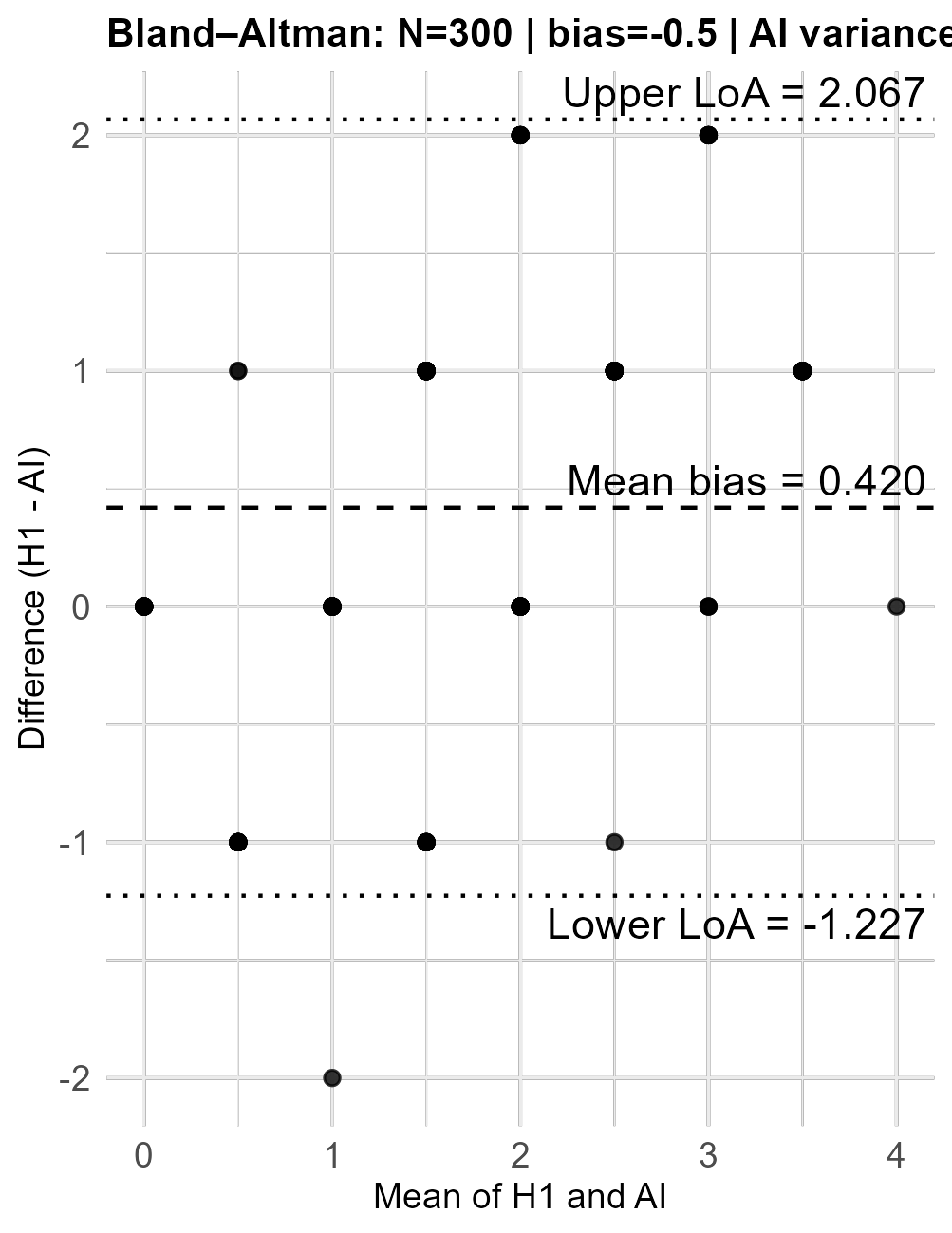

Supplement: Supplementary file 4 [file Data_Sheet_4.zip › BA_N300_bias-0.5_ailow_compTRUE_imbTRUE_fairFALSE_rep1.tif]

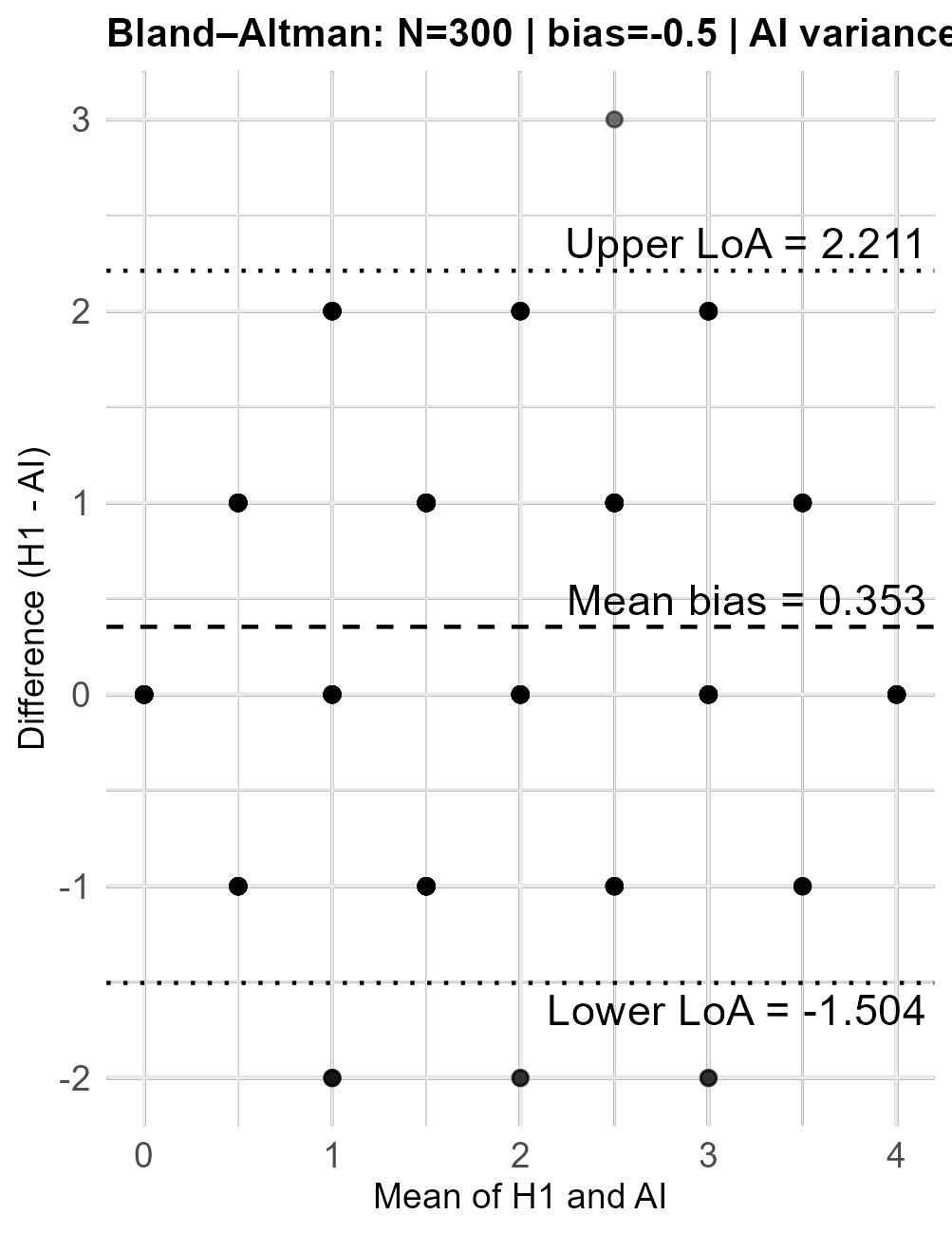

Supplement: Supplementary file 4 [file Data_Sheet_4.zip › BA_N300_bias-0.5_ailow_compFALSE_imbTRUE_fairTRUE_rep1.tif]

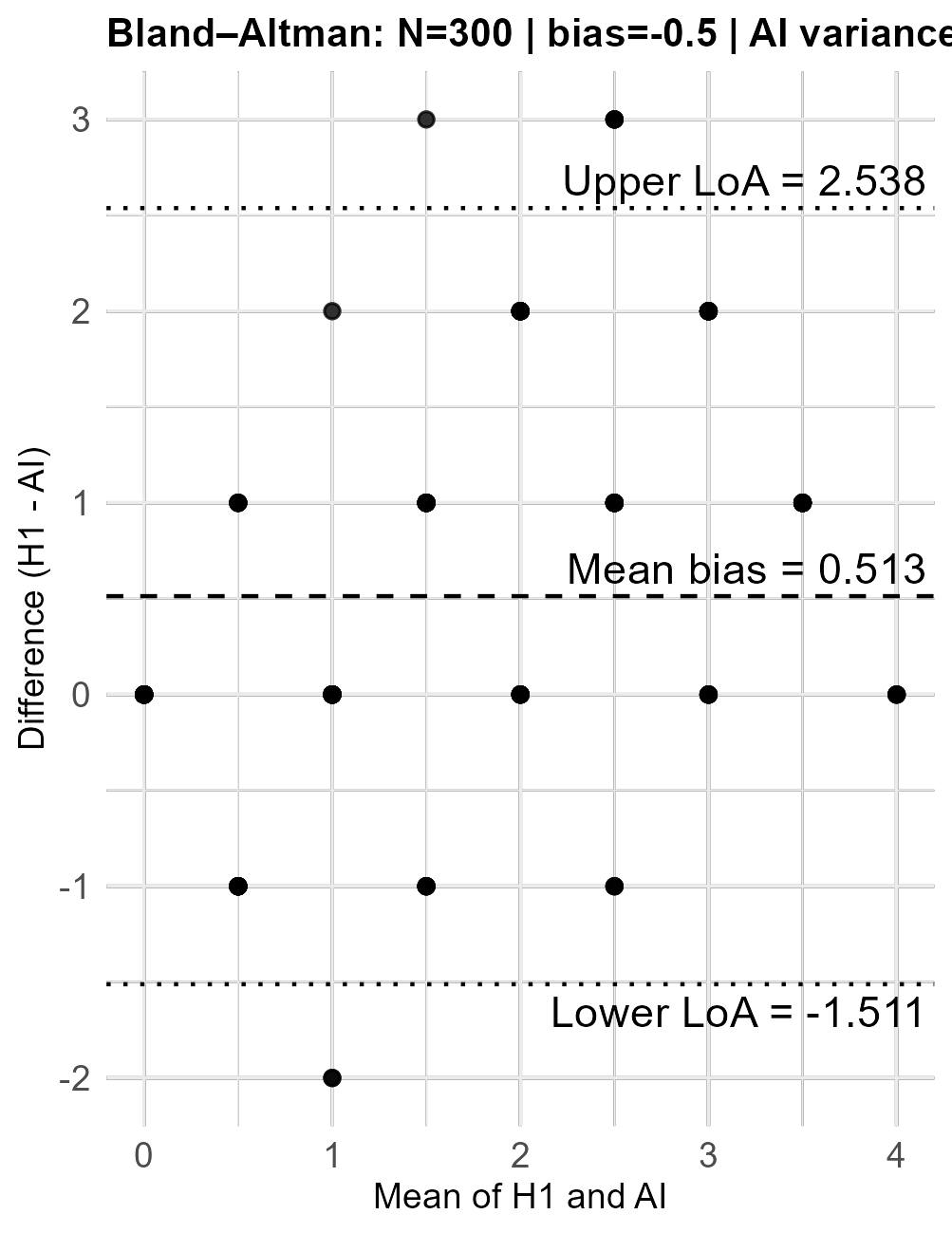

Supplement: Supplementary file 4 [file Data_Sheet_4.zip › BA_N300_bias-0.5_ailow_compTRUE_imbFALSE_fairFALSE_rep1.tif]

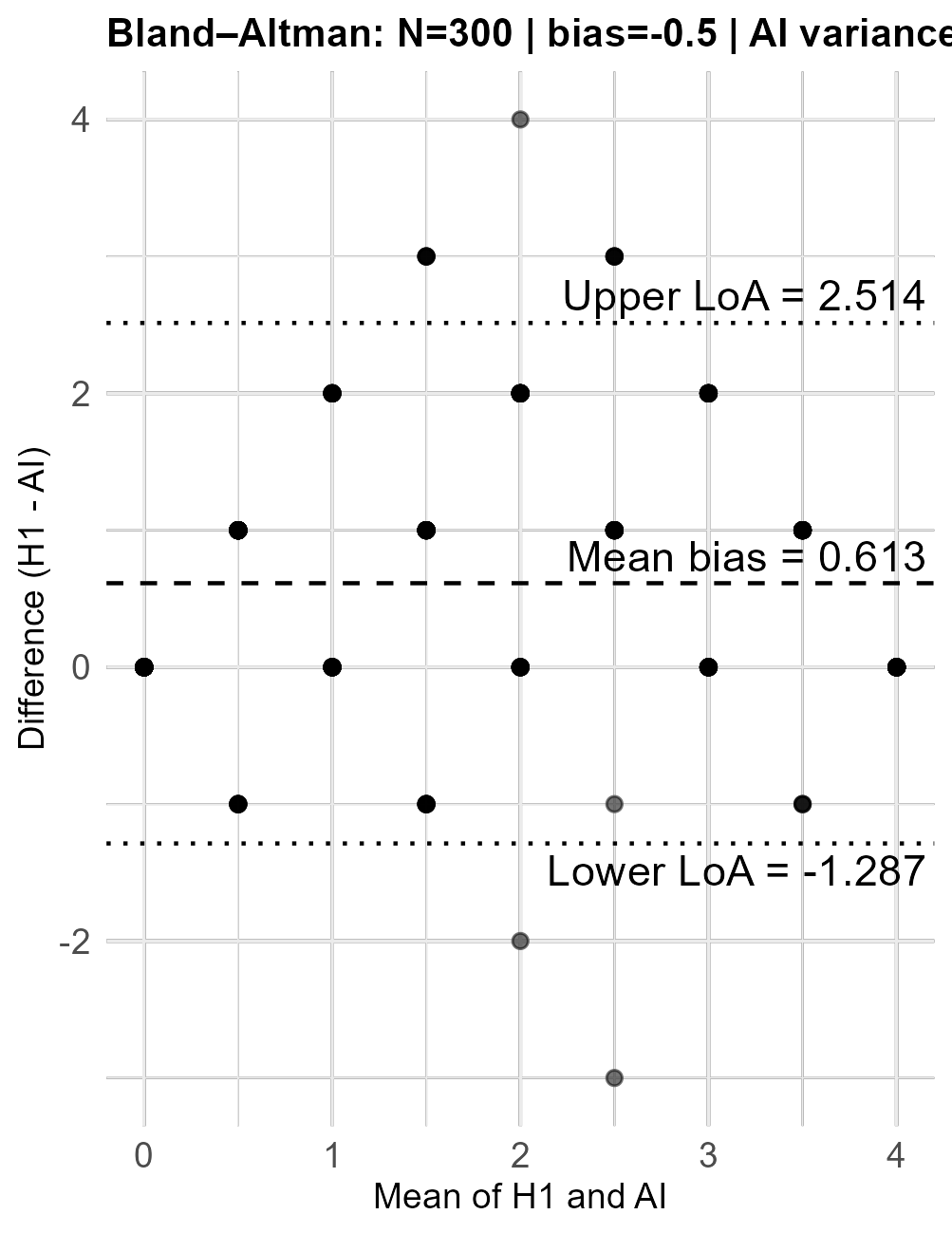

Supplement: Supplementary file 4 [file Data_Sheet_4.zip › BA_N300_bias-0.5_ailow_compFALSE_imbFALSE_fairFALSE_rep1.tif]

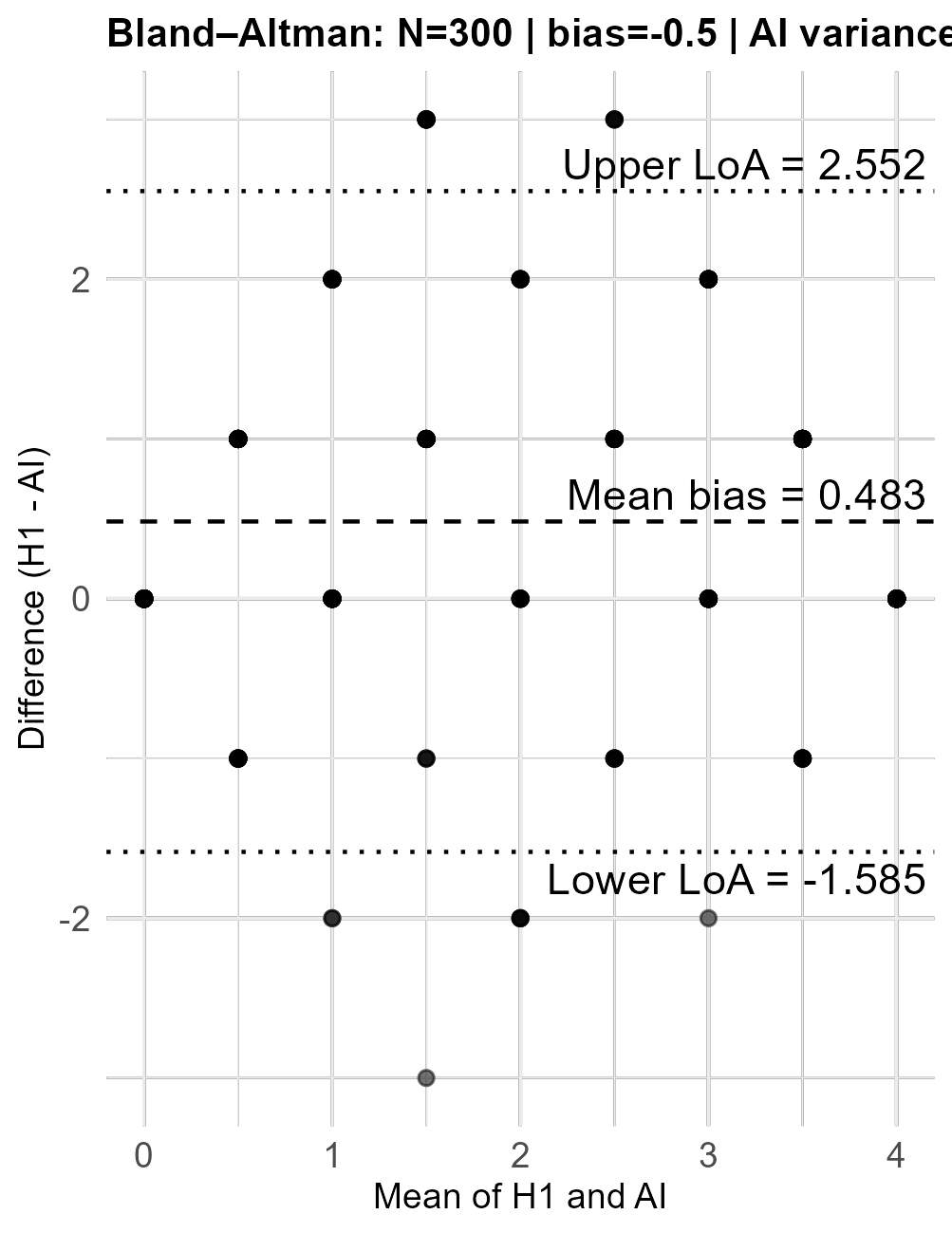

Supplement: Supplementary file 4 [file Data_Sheet_4.zip › BA_N300_bias-0.5_ailow_compFALSE_imbFALSE_fairTRUE_rep1.tif]

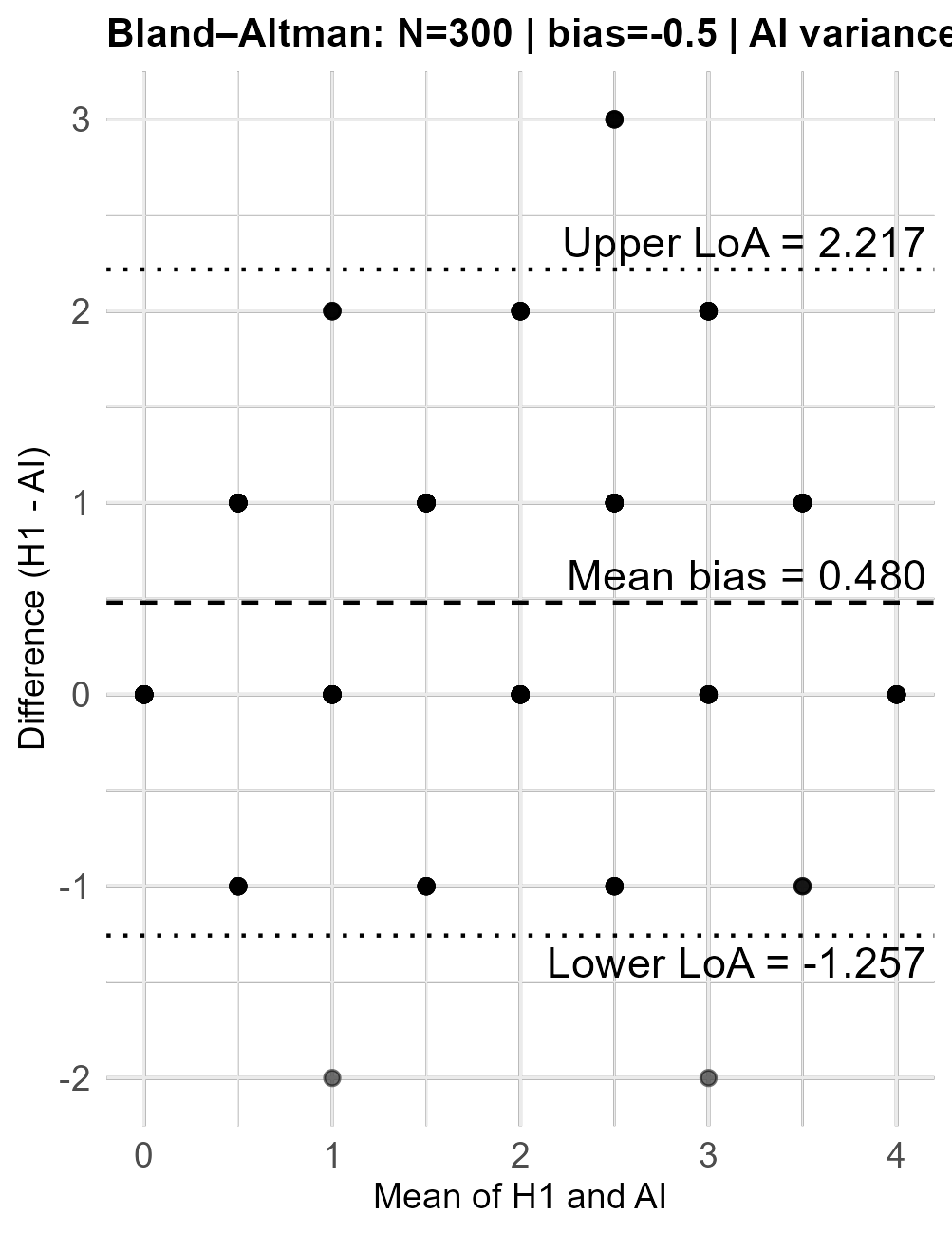

Supplement: Supplementary file 4 [file Data_Sheet_4.zip › BA_N300_bias-0.5_ailow_compFALSE_imbTRUE_fairFALSE_rep1.tif]

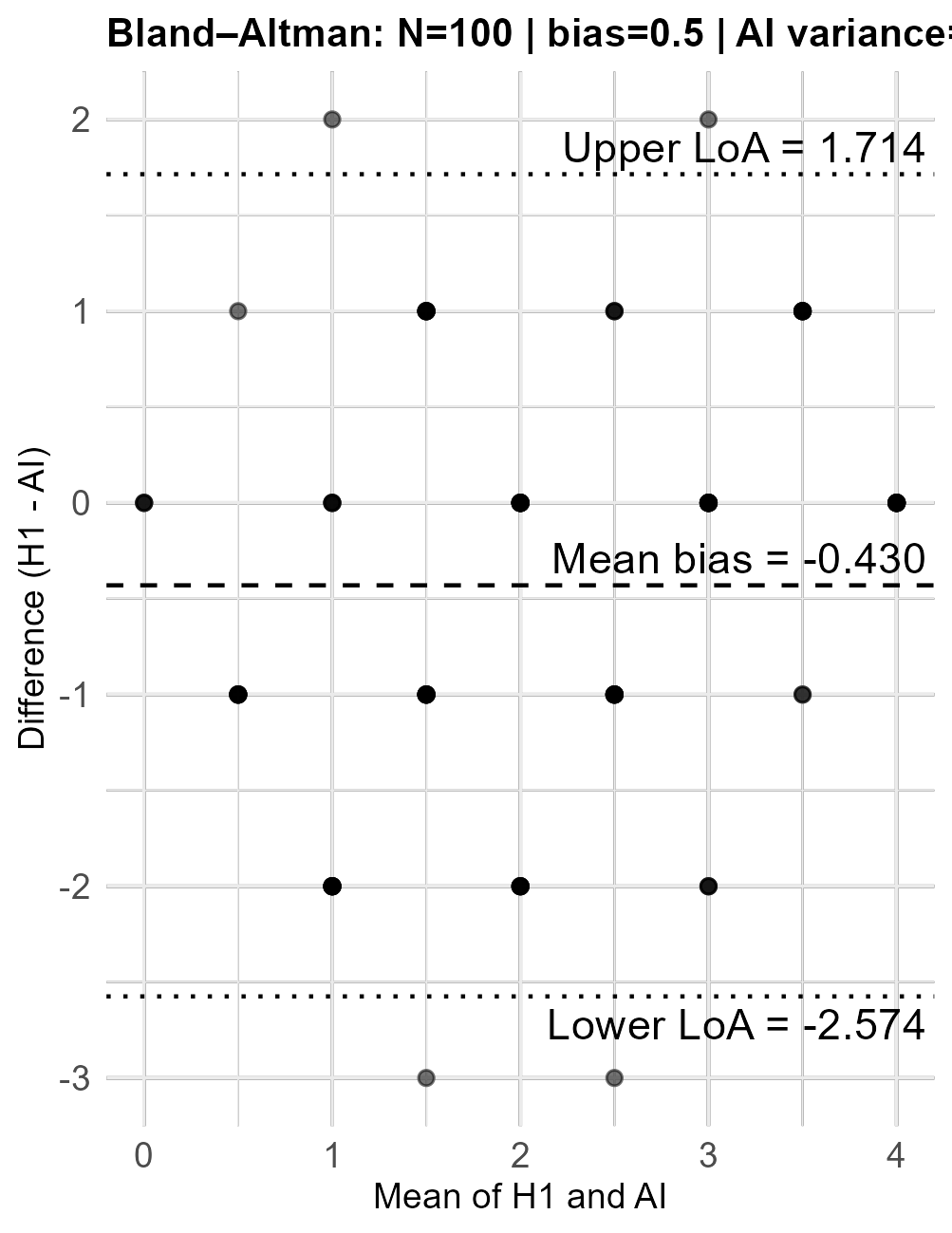

Supplement: Supplementary file 4 [file Data_Sheet_4.zip › BA_N100_bias0.5_aihigh_compTRUE_imbTRUE_fairFALSE_rep1.tif]

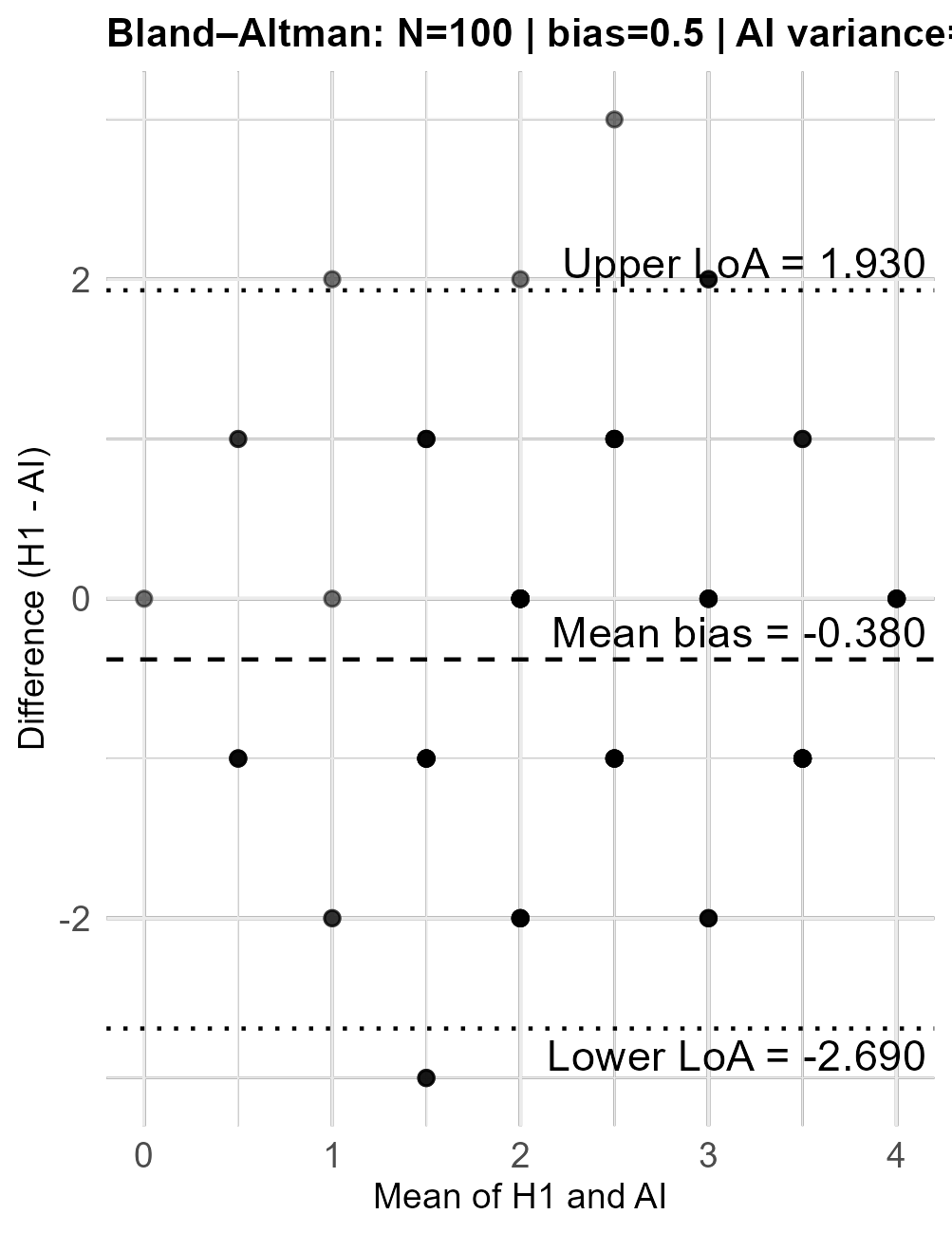

Supplement: Supplementary file 4 [file Data_Sheet_4.zip › BA_N100_bias0.5_aihigh_compTRUE_imbTRUE_fairTRUE_rep1.tif]

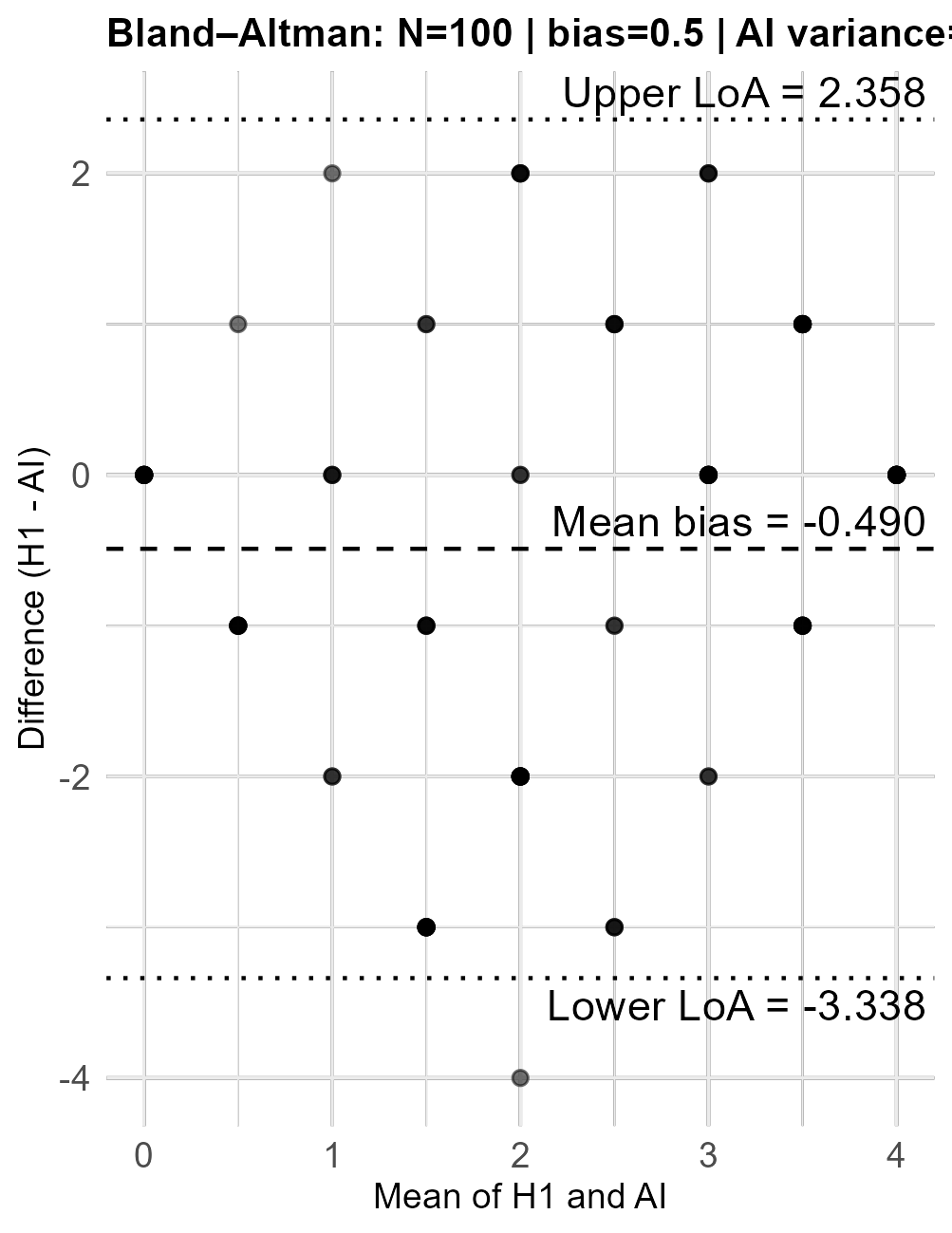

Supplement: Supplementary file 4 [file Data_Sheet_4.zip › BA_N100_bias0.5_aihigh_compTRUE_imbFALSE_fairFALSE_rep1.tif]

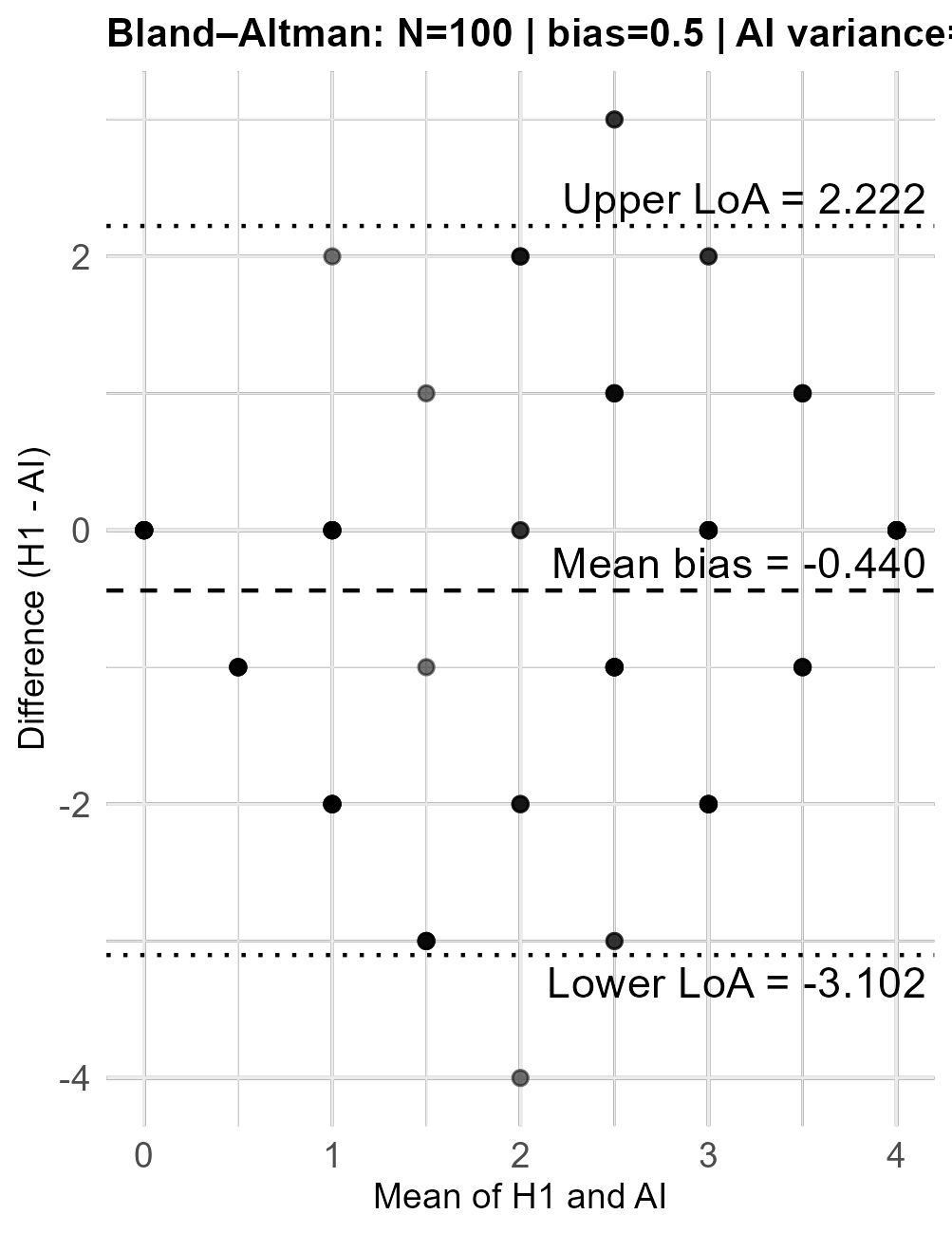

Supplement: Supplementary file 4 [file Data_Sheet_4.zip › BA_N100_bias0.5_aihigh_compTRUE_imbFALSE_fairTRUE_rep1.tif]

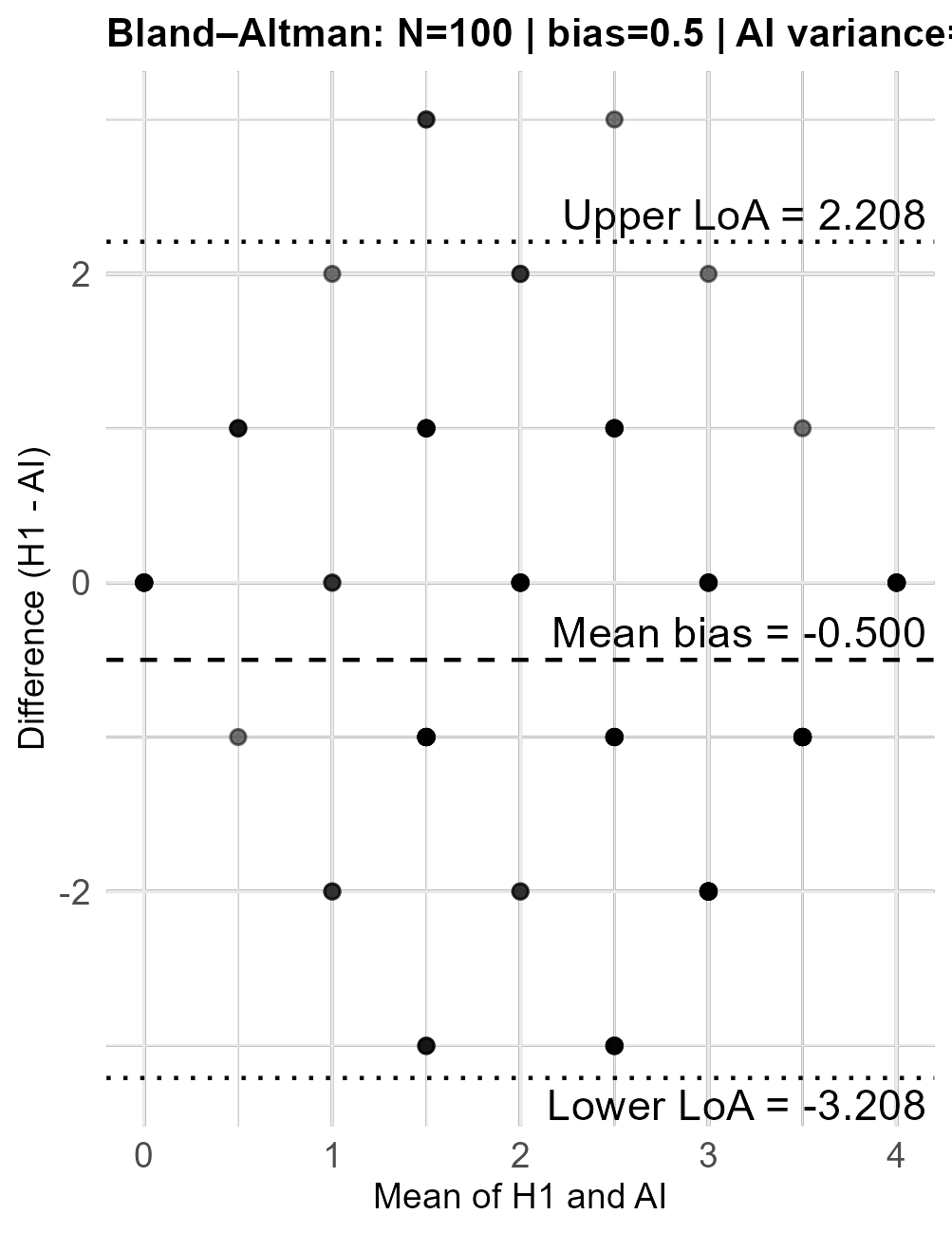

Supplement: Supplementary file 4 [file Data_Sheet_4.zip › BA_N100_bias0.5_aihigh_compFALSE_imbTRUE_fairFALSE_rep1.tif]

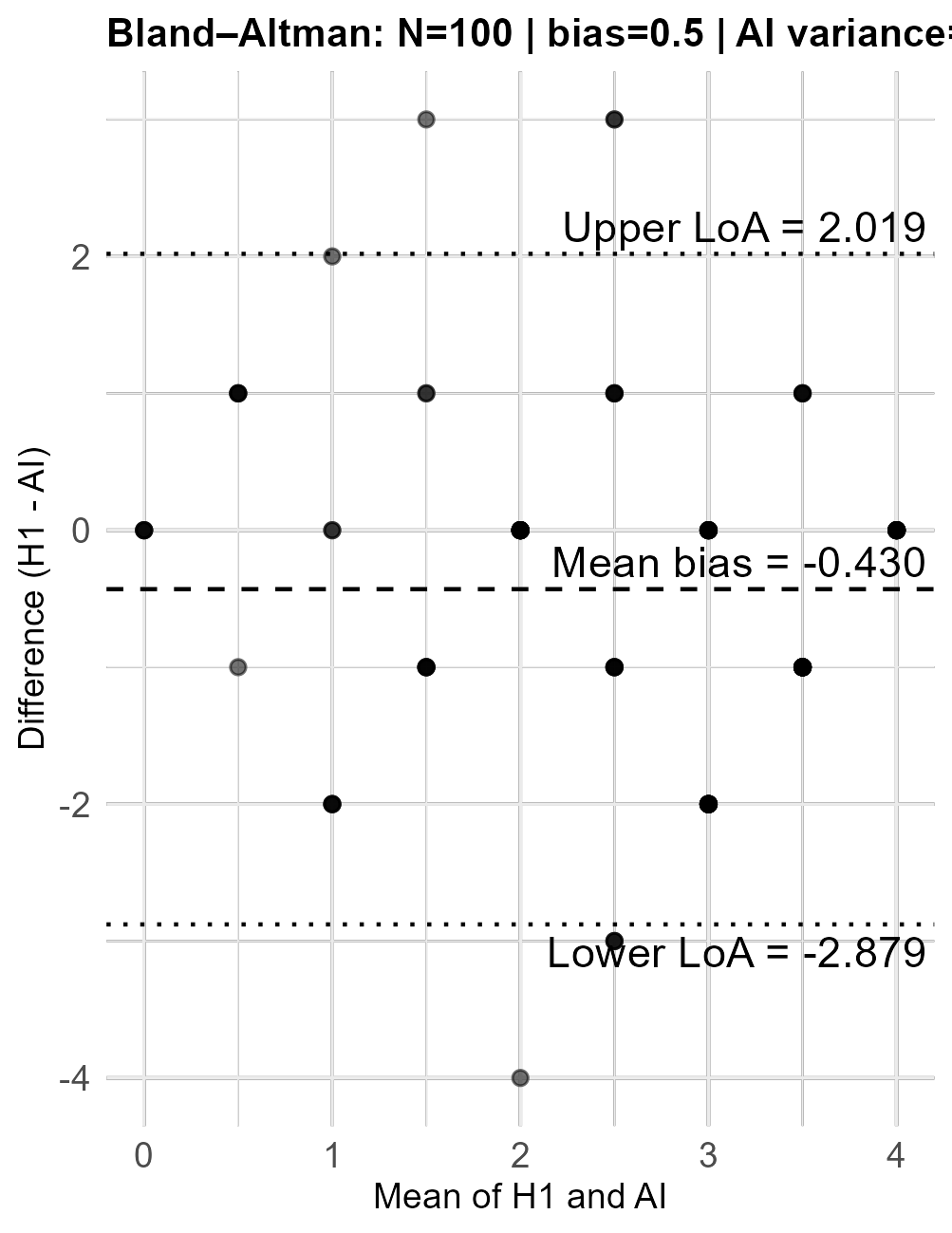

Supplement: Supplementary file 4 [file Data_Sheet_4.zip › BA_N100_bias0.5_aihigh_compFALSE_imbTRUE_fairTRUE_rep1.tif]

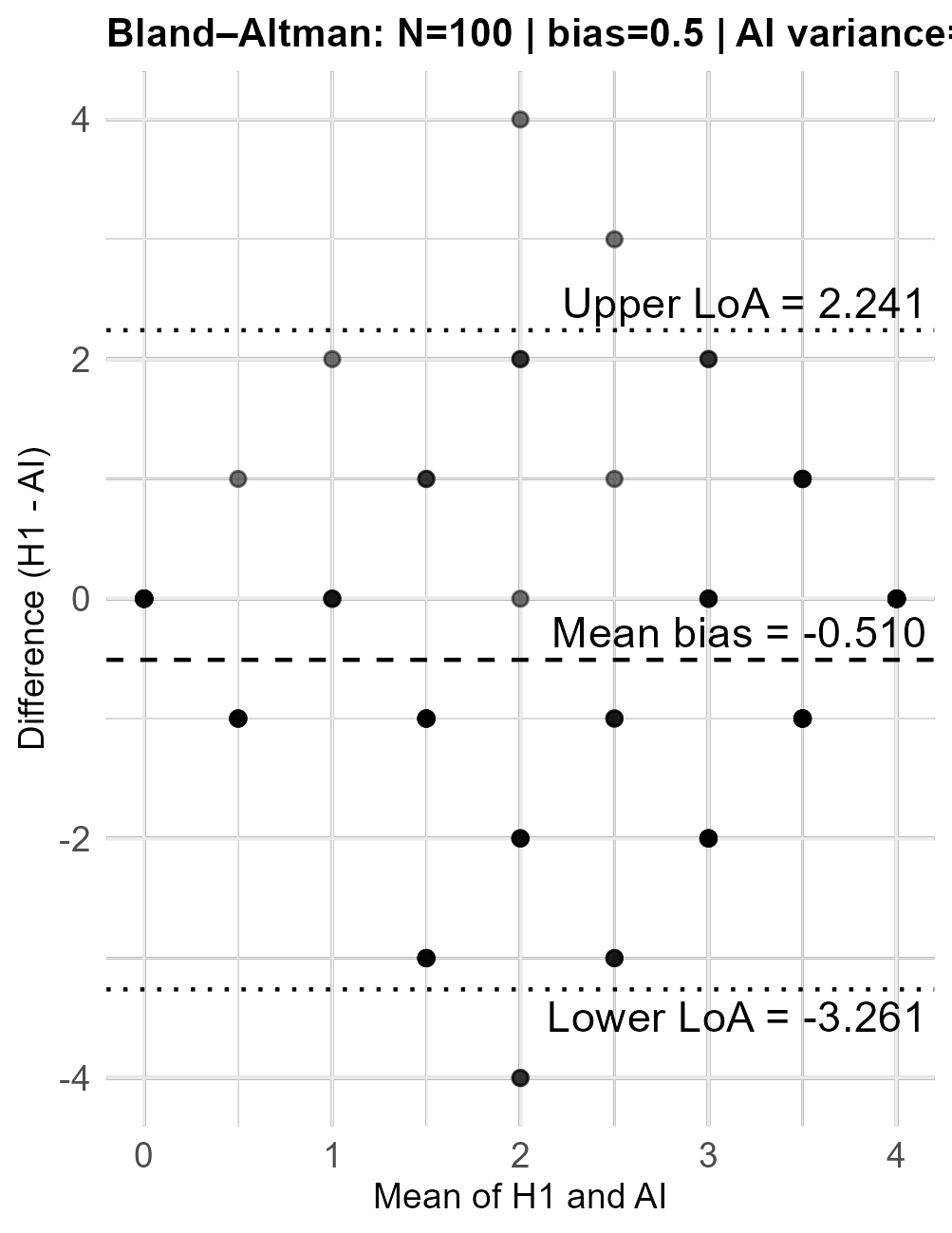

Supplement: Supplementary file 4 [file Data_Sheet_4.zip › BA_N100_bias0.5_aihigh_compFALSE_imbFALSE_fairFALSE_rep1.tif]

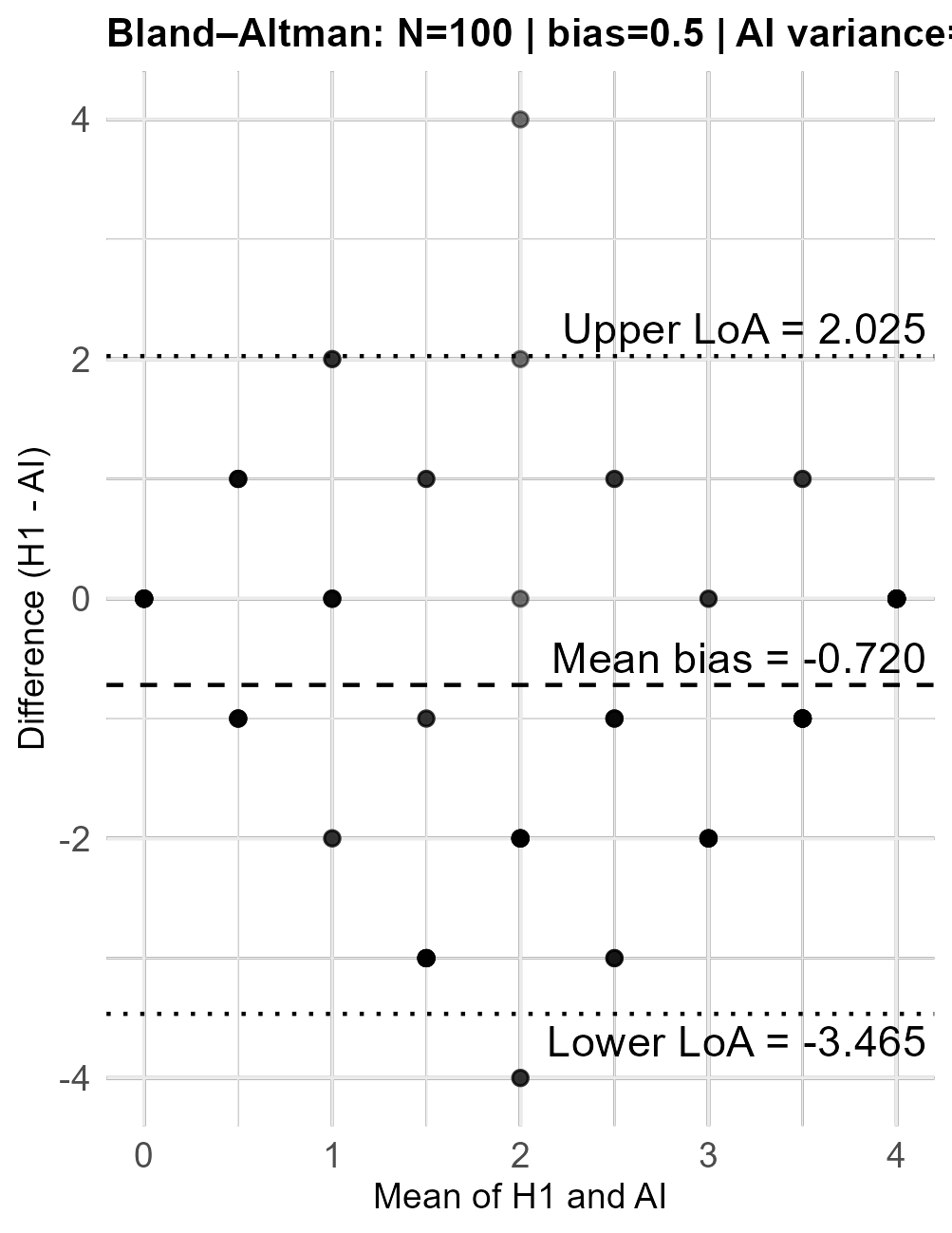

Supplement: Supplementary file 4 [file Data_Sheet_4.zip › BA_N100_bias0.5_aihigh_compFALSE_imbFALSE_fairTRUE_rep1.tif]

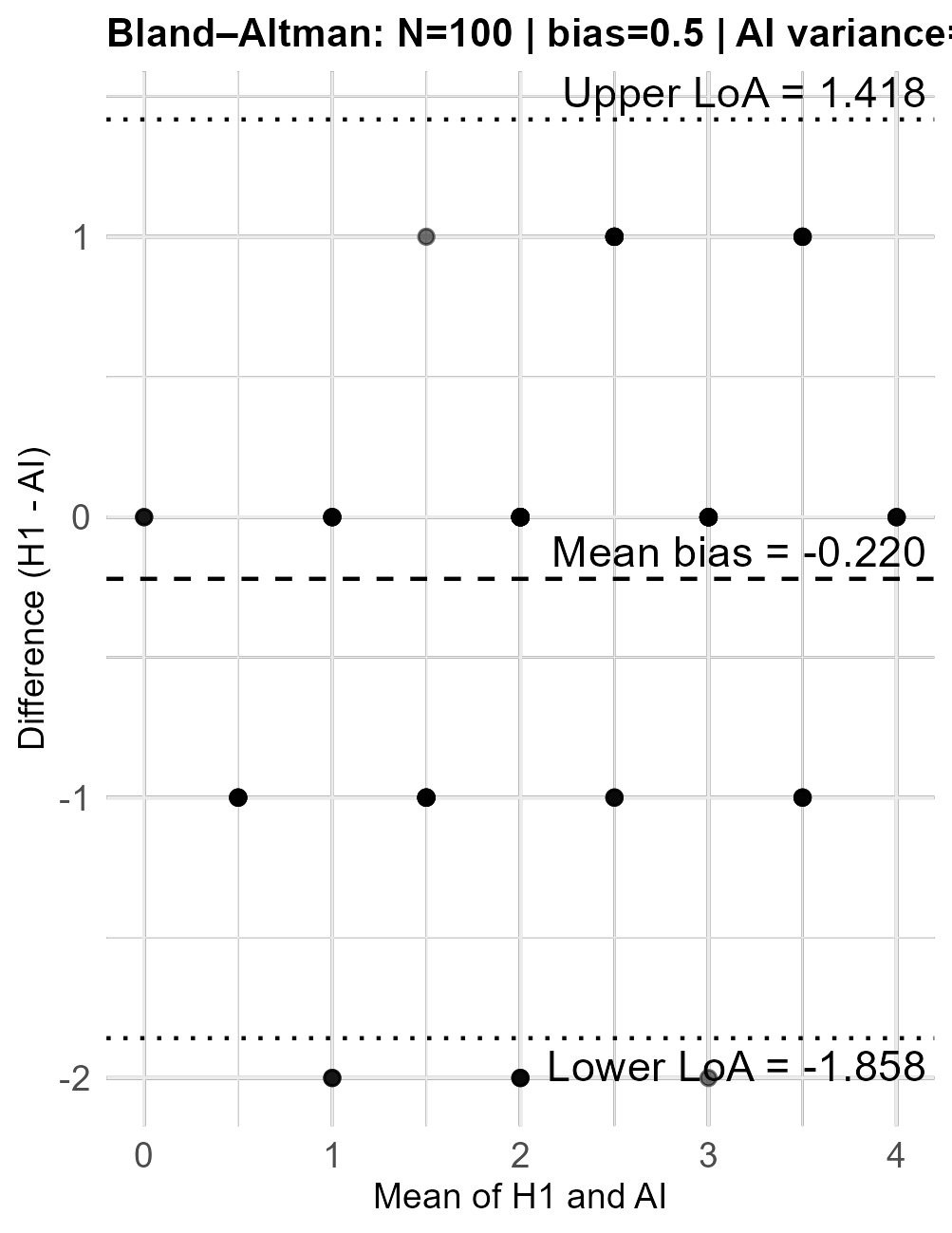

Supplement: Supplementary file 4 [file Data_Sheet_4.zip › BA_N100_bias0.5_aimid_compTRUE_imbTRUE_fairTRUE_rep1.tif]

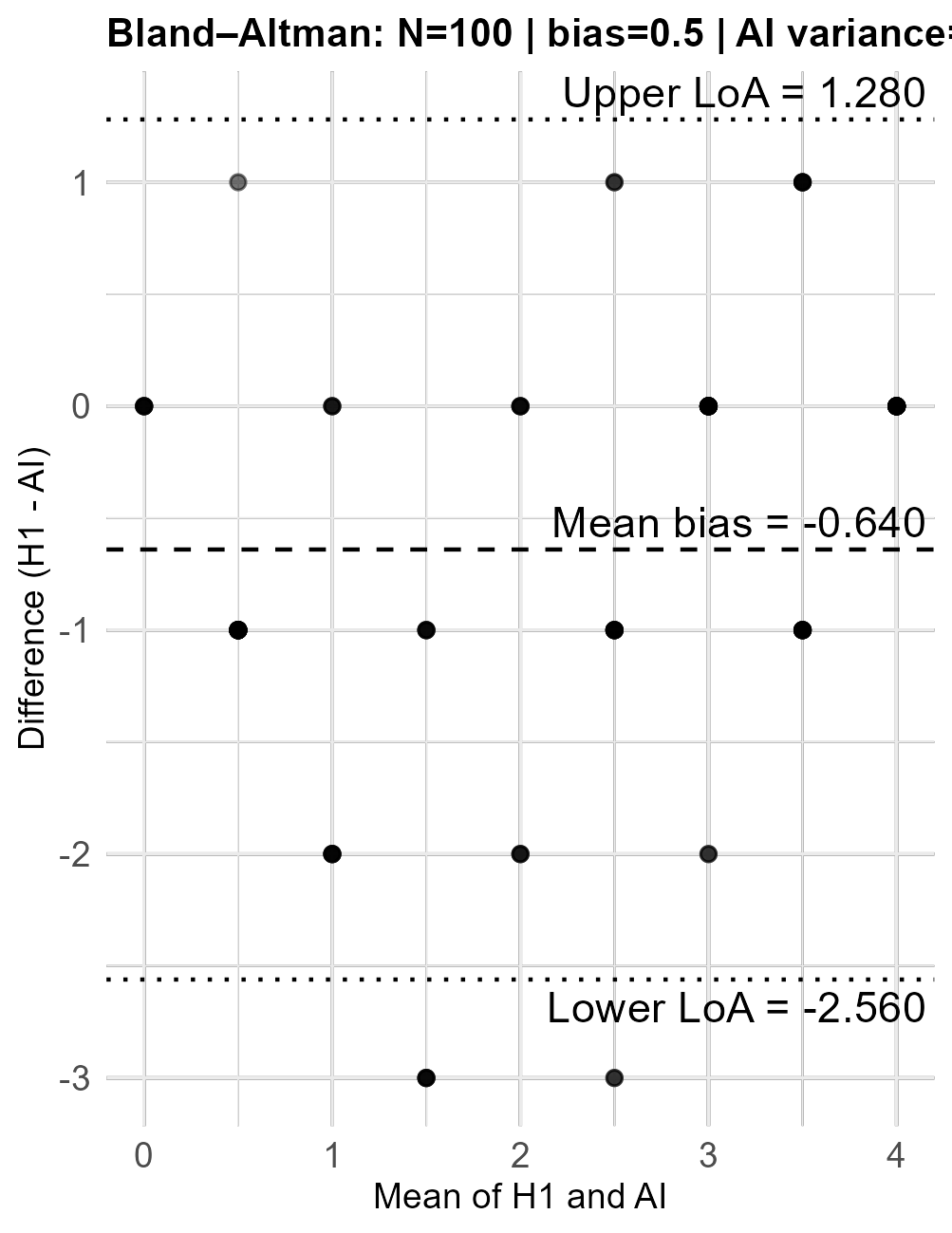

Supplement: Supplementary file 4 [file Data_Sheet_4.zip › BA_N100_bias0.5_aimid_compTRUE_imbFALSE_fairTRUE_rep1.tif]

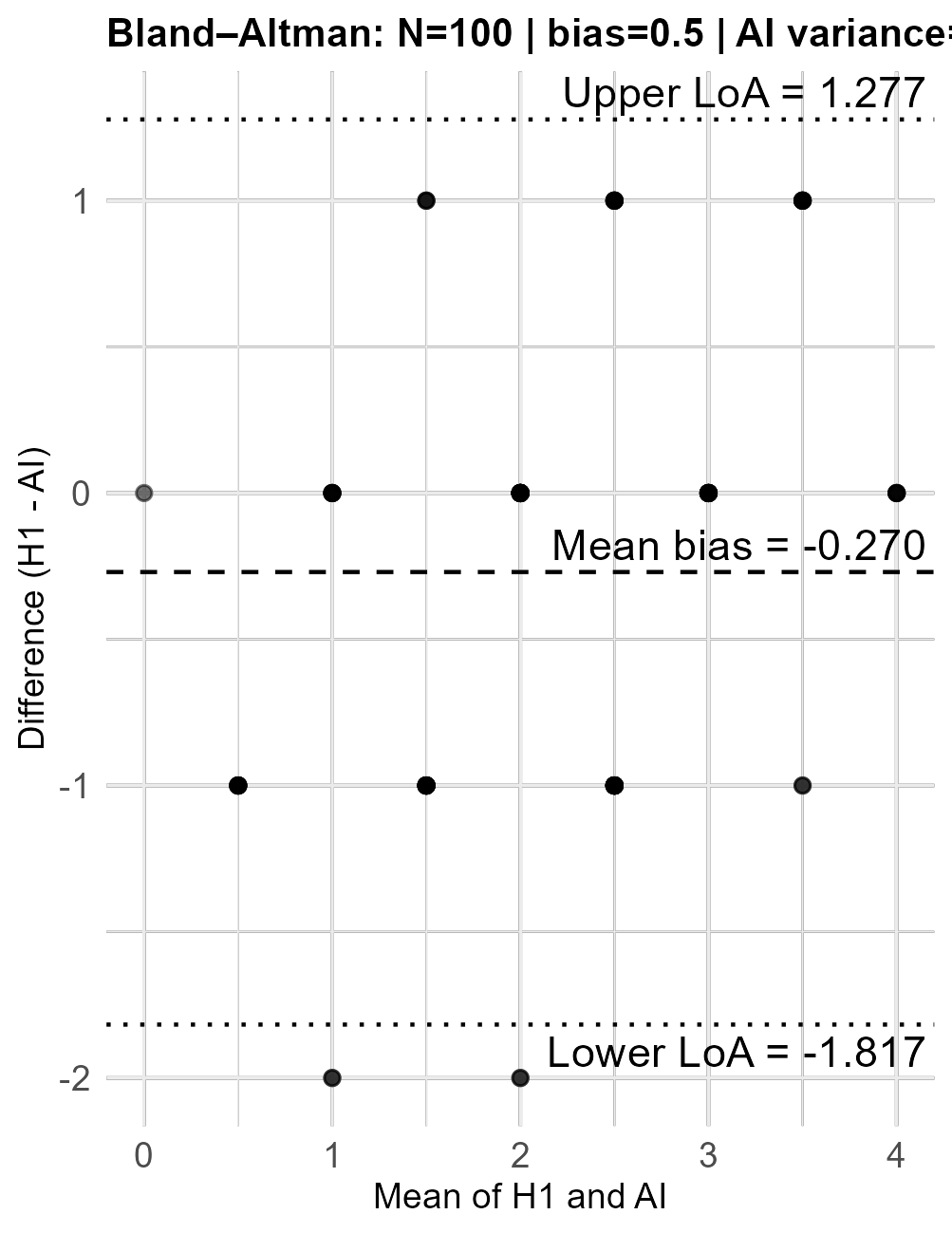

Supplement: Supplementary file 4 [file Data_Sheet_4.zip › BA_N100_bias0.5_aimid_compTRUE_imbTRUE_fairFALSE_rep1.tif]

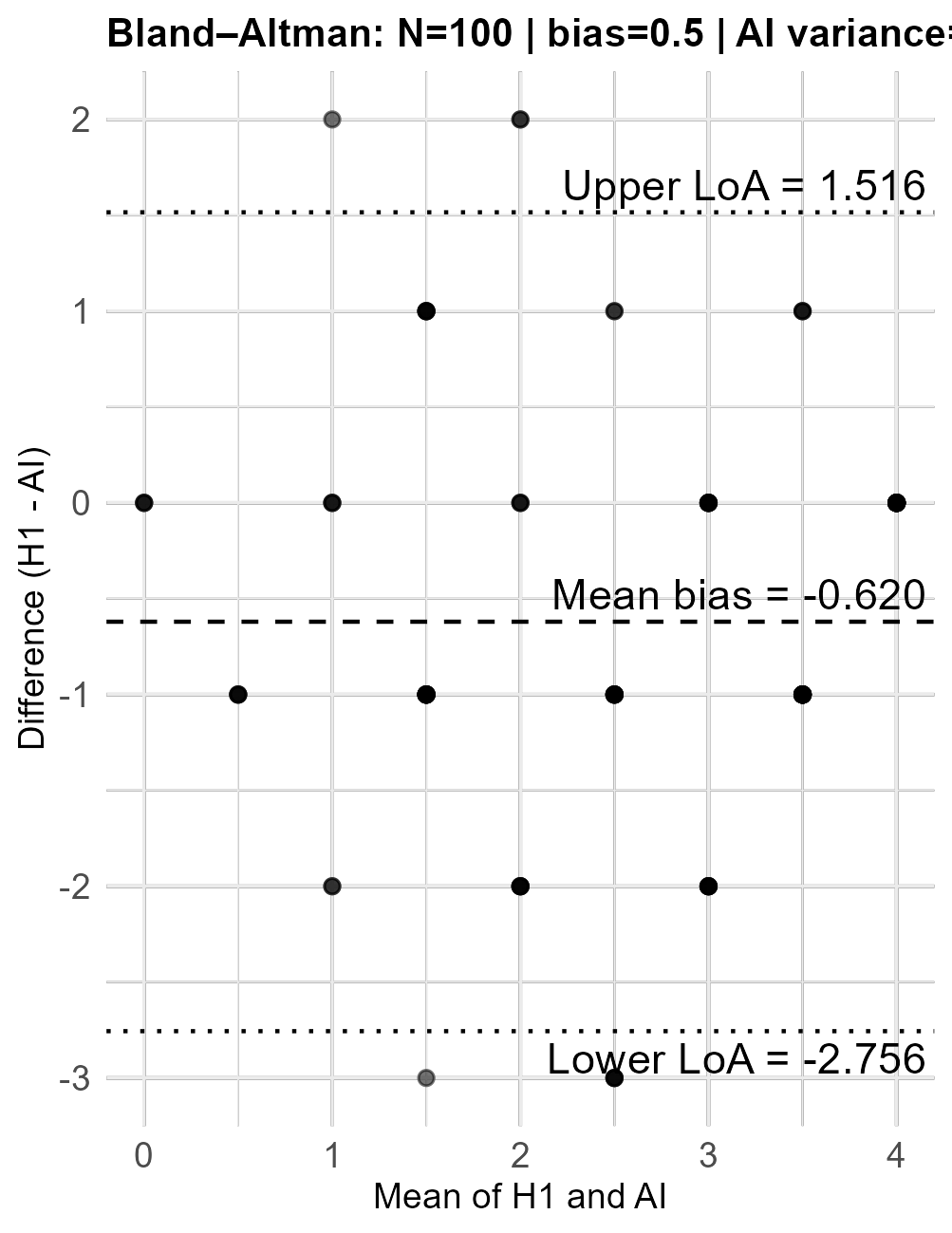

Supplement: Supplementary file 4 [file Data_Sheet_4.zip › BA_N100_bias0.5_aimid_compFALSE_imbTRUE_fairTRUE_rep1.tif]

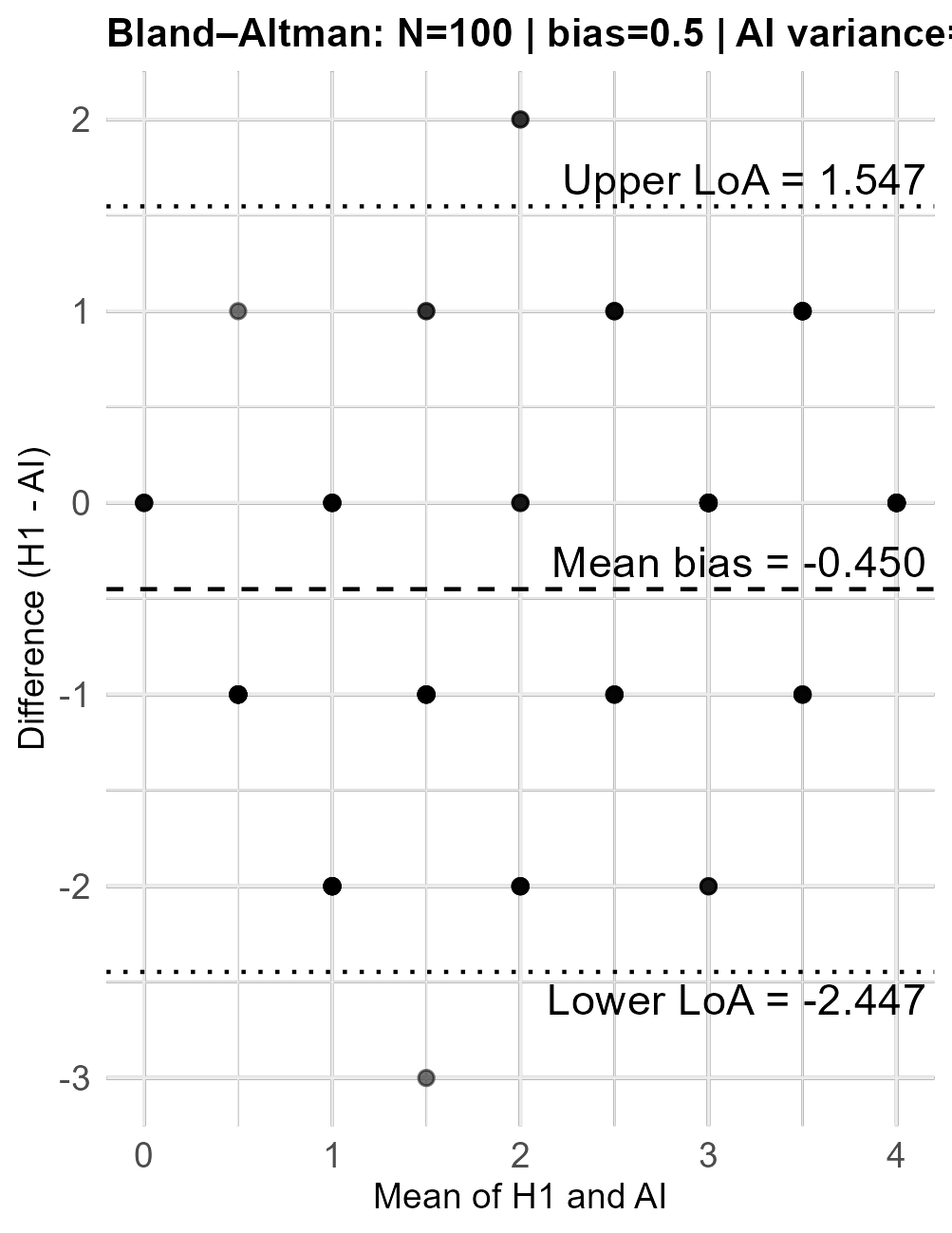

Supplement: Supplementary file 4 [file Data_Sheet_4.zip › BA_N100_bias0.5_aimid_compTRUE_imbFALSE_fairFALSE_rep1.tif]

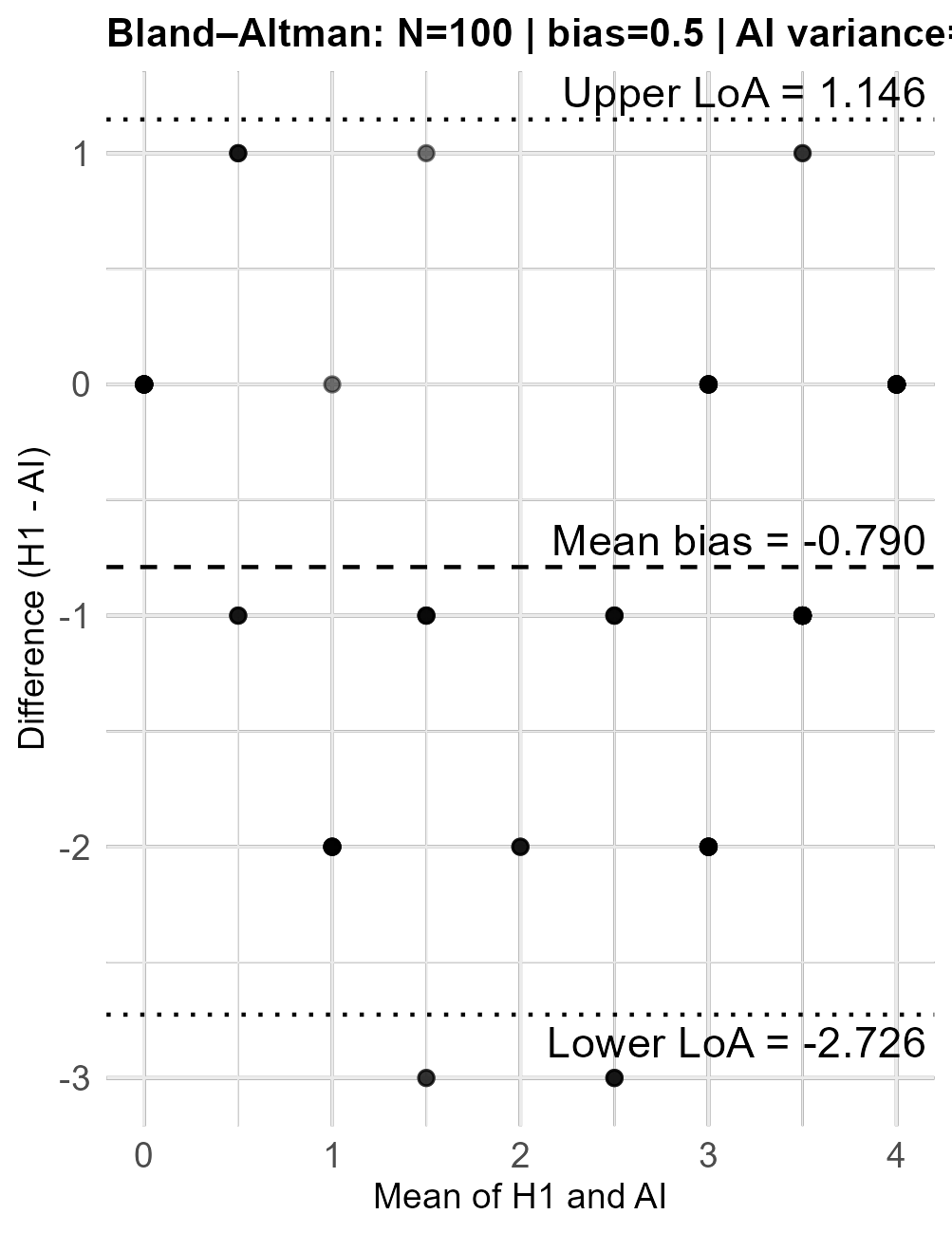

Supplement: Supplementary file 4 [file Data_Sheet_4.zip › BA_N100_bias0.5_aimid_compFALSE_imbFALSE_fairTRUE_rep1.tif]

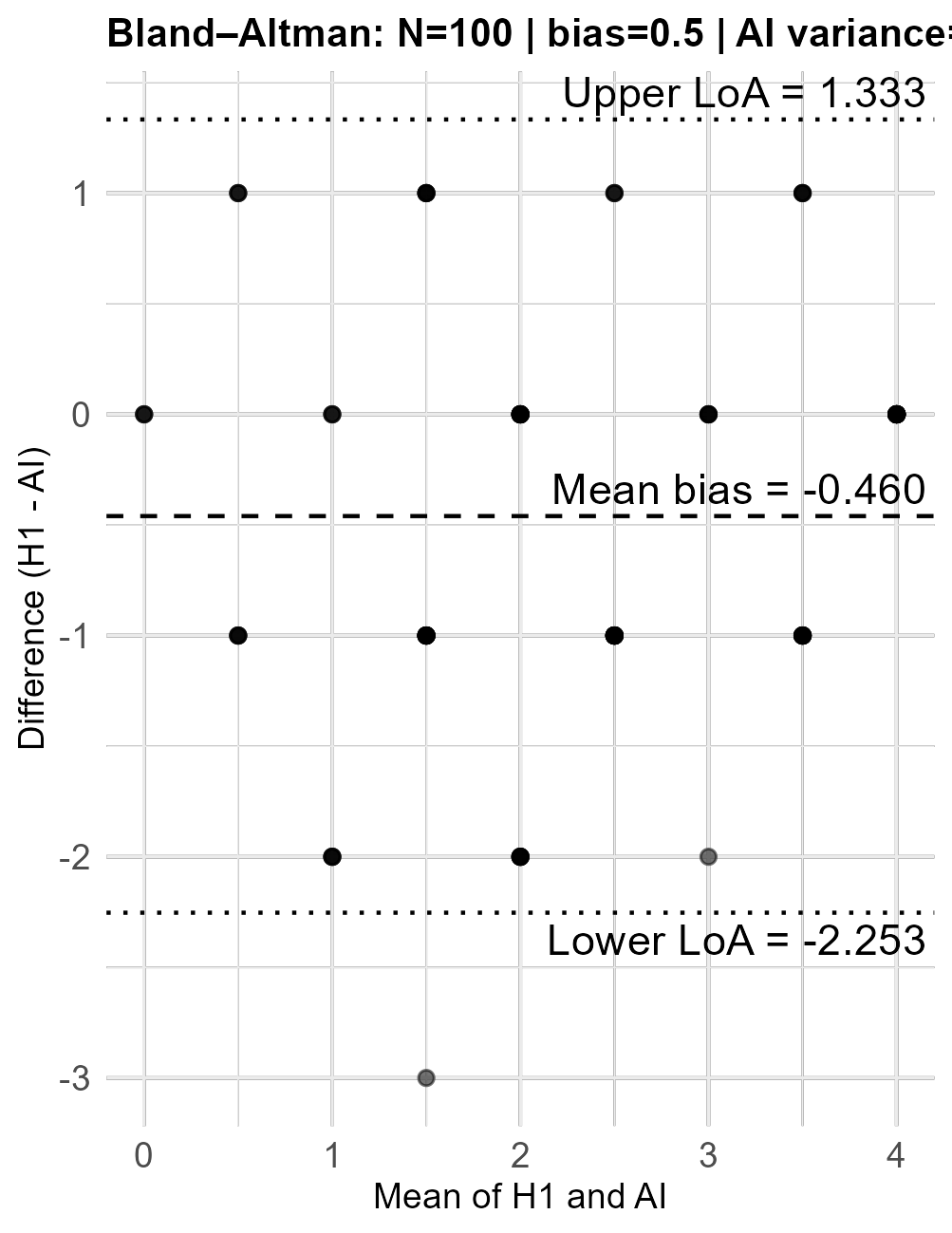

Supplement: Supplementary file 4 [file Data_Sheet_4.zip › BA_N100_bias0.5_aimid_compFALSE_imbTRUE_fairFALSE_rep1.tif]

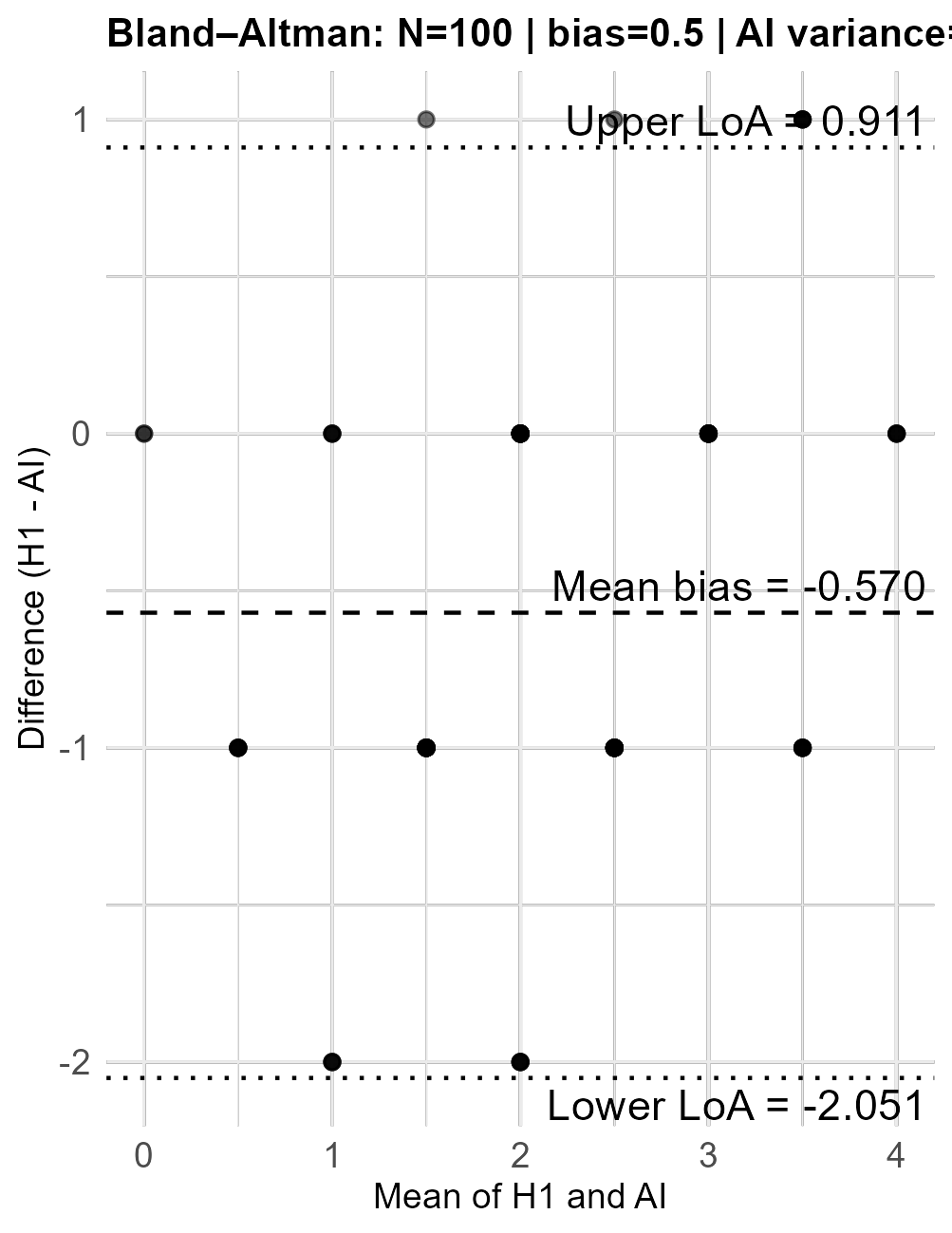

Supplement: Supplementary file 4 [file Data_Sheet_4.zip › BA_N100_bias0.5_ailow_compTRUE_imbTRUE_fairTRUE_rep1.tif]

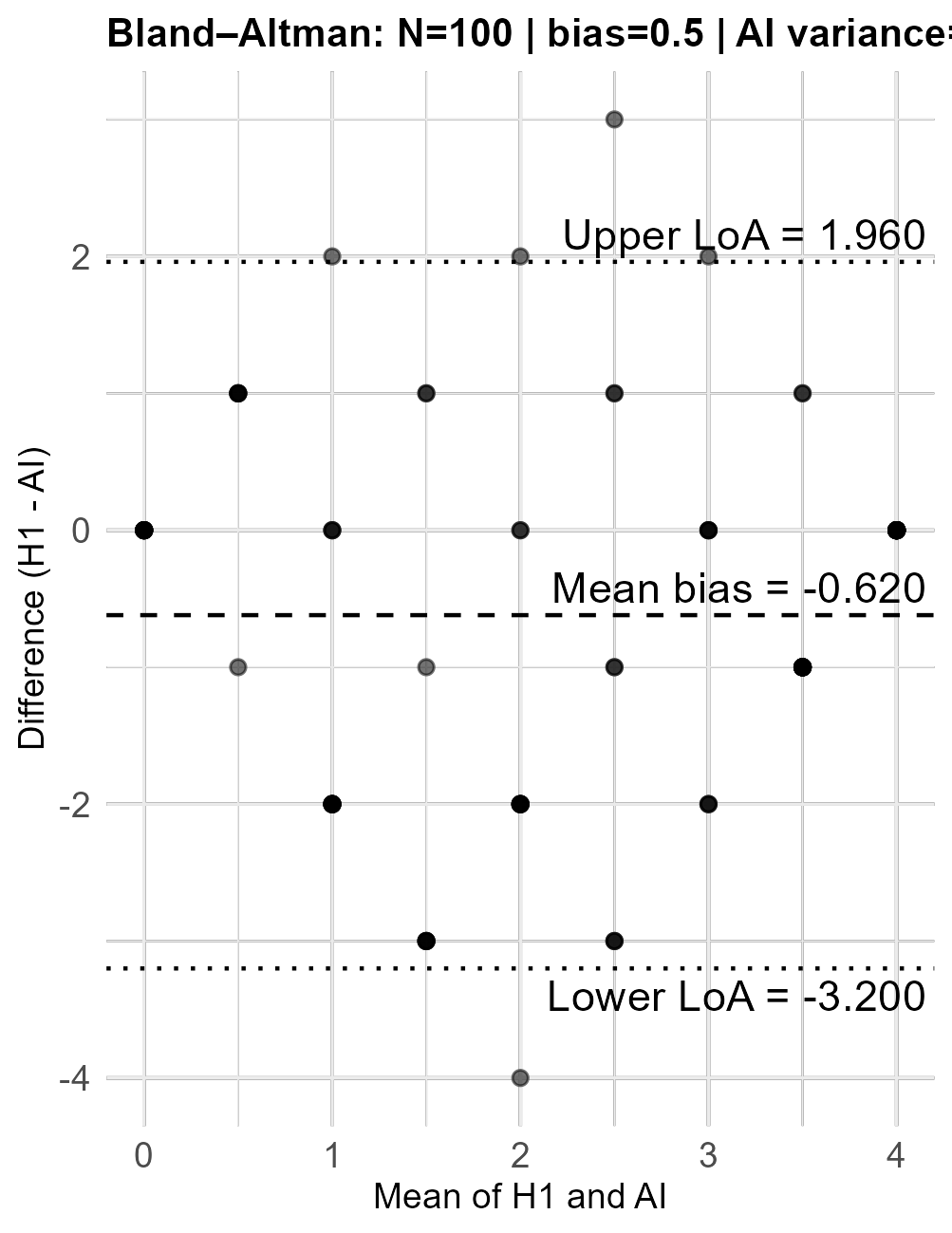

Supplement: Supplementary file 4 [file Data_Sheet_4.zip › BA_N100_bias0.5_aimid_compFALSE_imbFALSE_fairFALSE_rep1.tif]

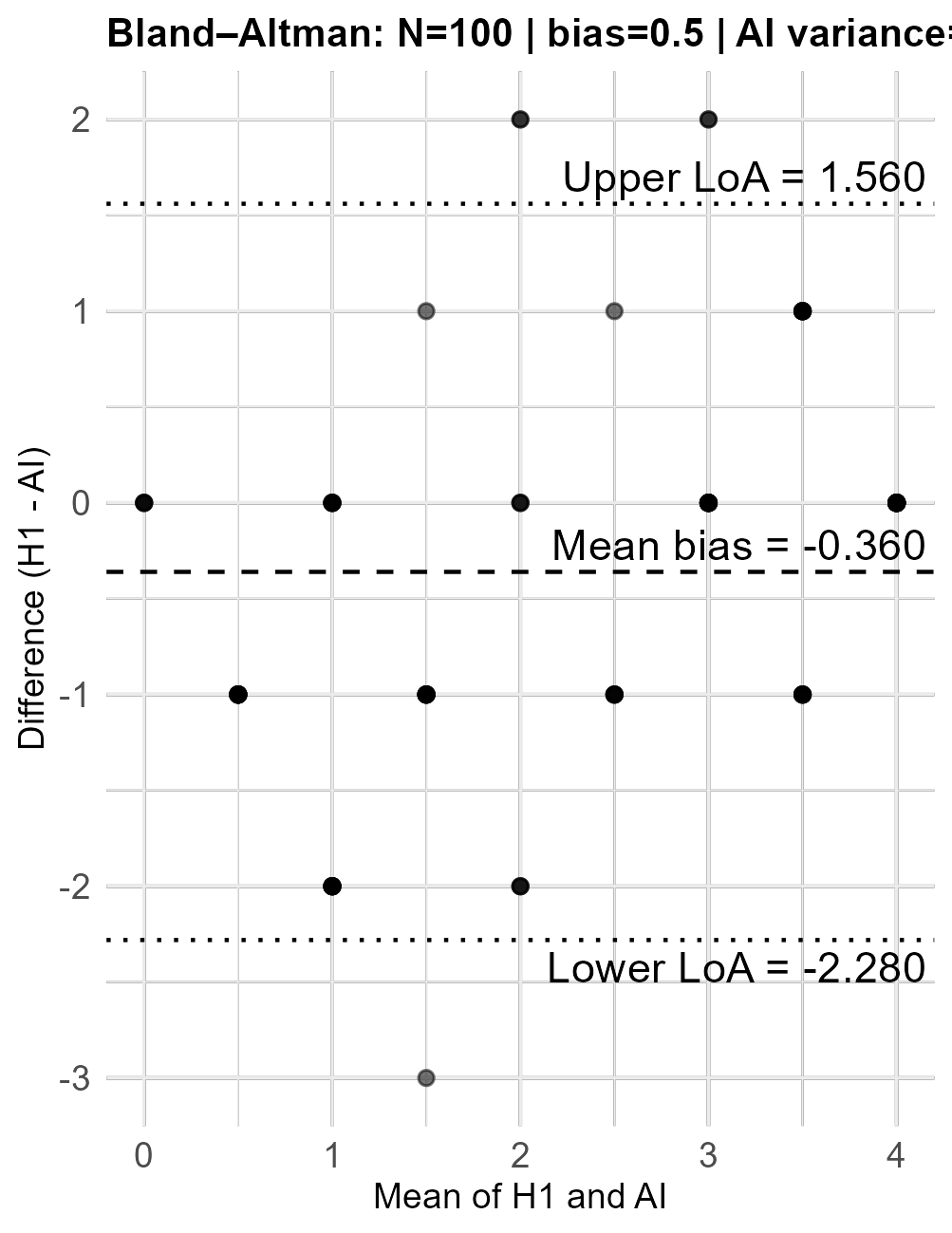

Supplement: Supplementary file 4 [file Data_Sheet_4.zip › BA_N100_bias0.5_ailow_compTRUE_imbFALSE_fairFALSE_rep1.tif]

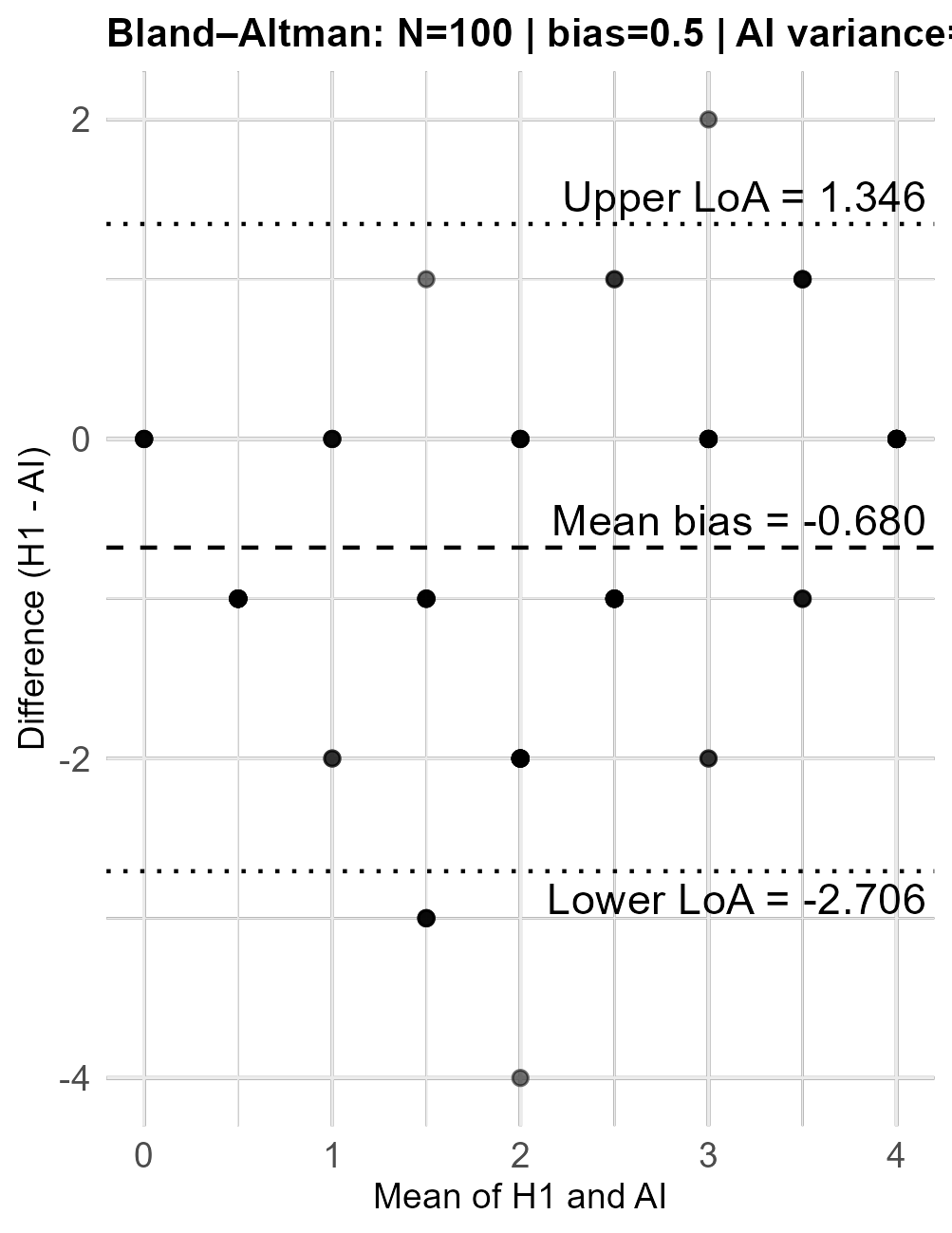

Supplement: Supplementary file 4 [file Data_Sheet_4.zip › BA_N100_bias0.5_ailow_compTRUE_imbFALSE_fairTRUE_rep1.tif]

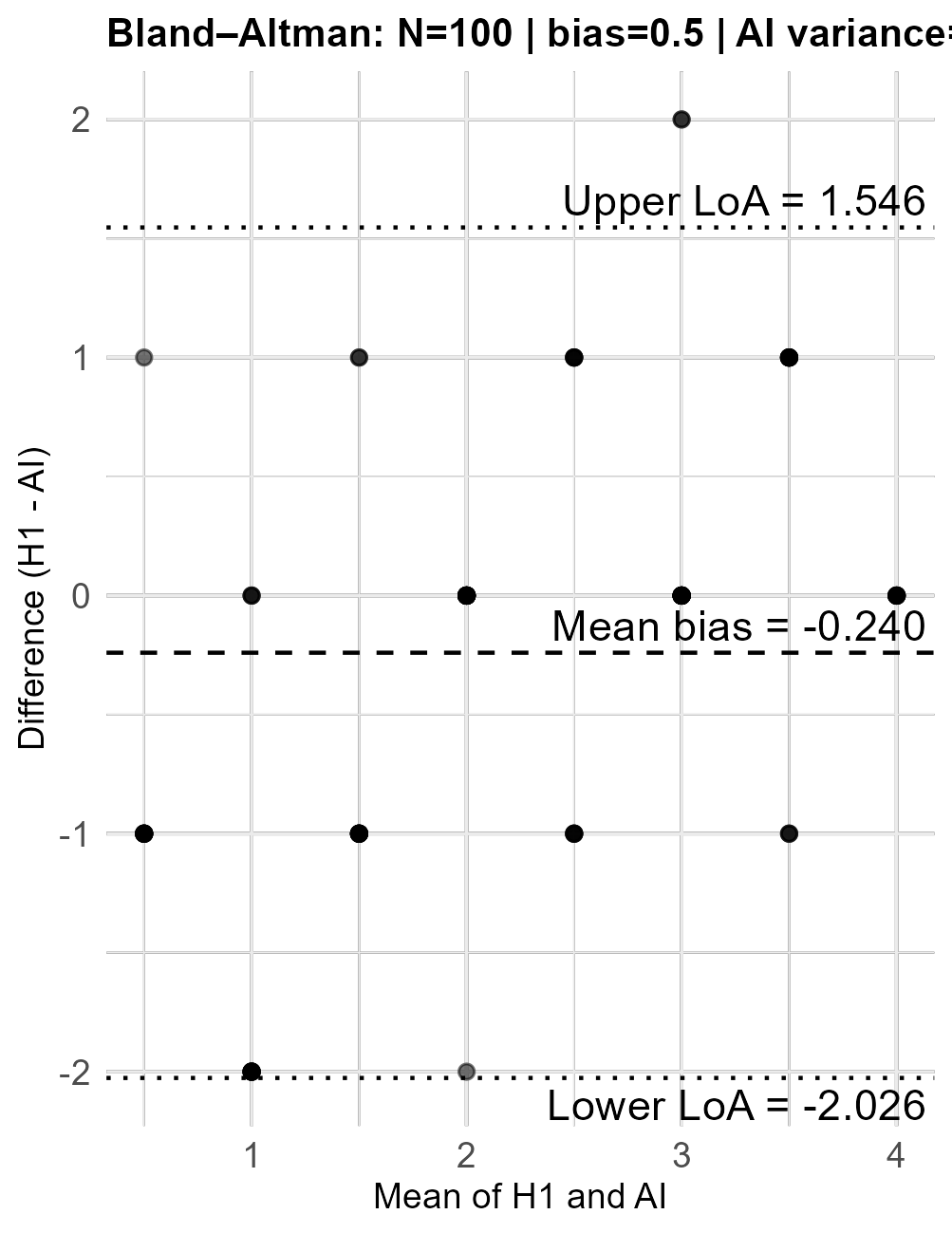

Supplement: Supplementary file 4 [file Data_Sheet_4.zip › BA_N100_bias0.5_ailow_compTRUE_imbTRUE_fairFALSE_rep1.tif]

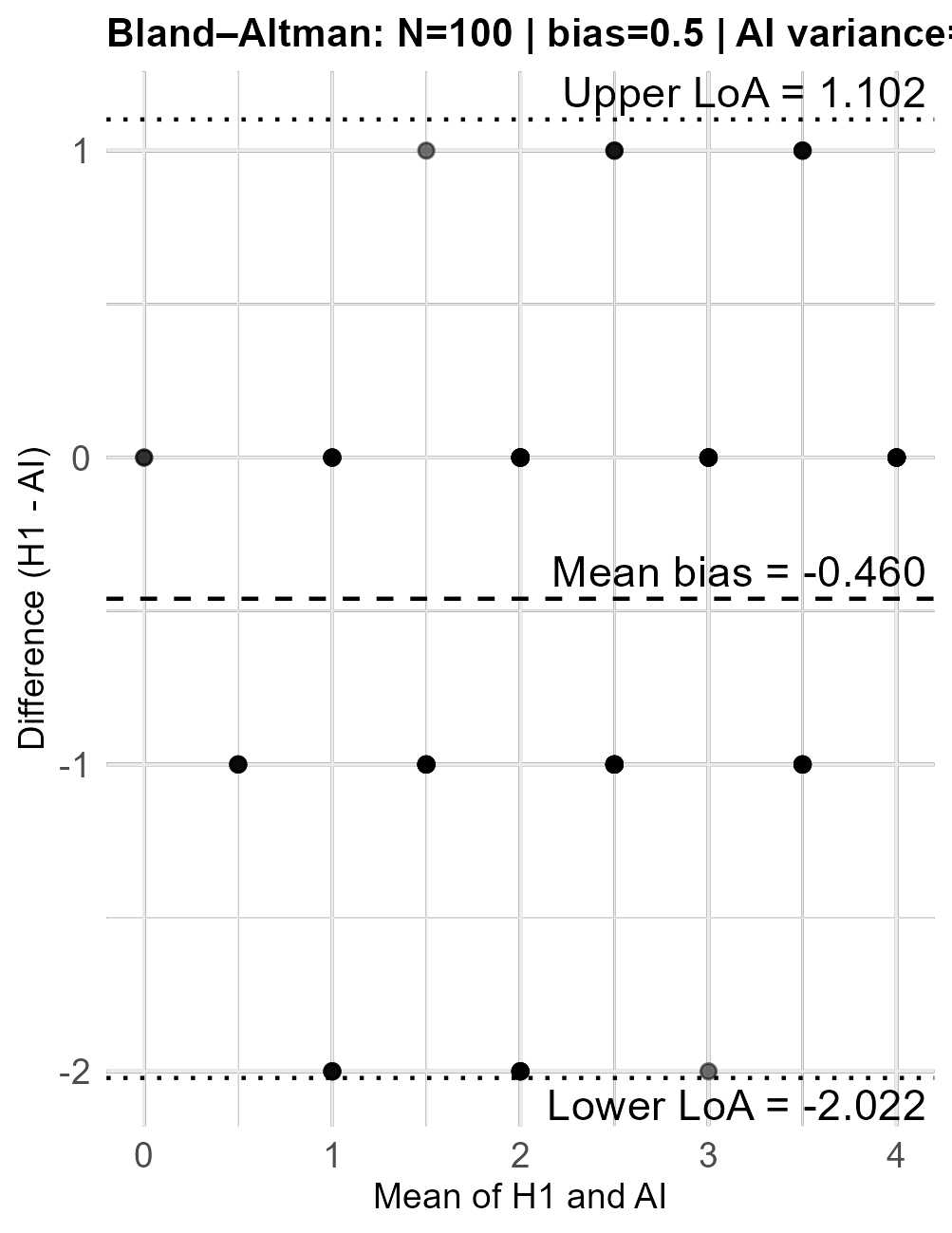

Supplement: Supplementary file 4 [file Data_Sheet_4.zip › BA_N100_bias0.5_ailow_compFALSE_imbTRUE_fairFALSE_rep1.tif]

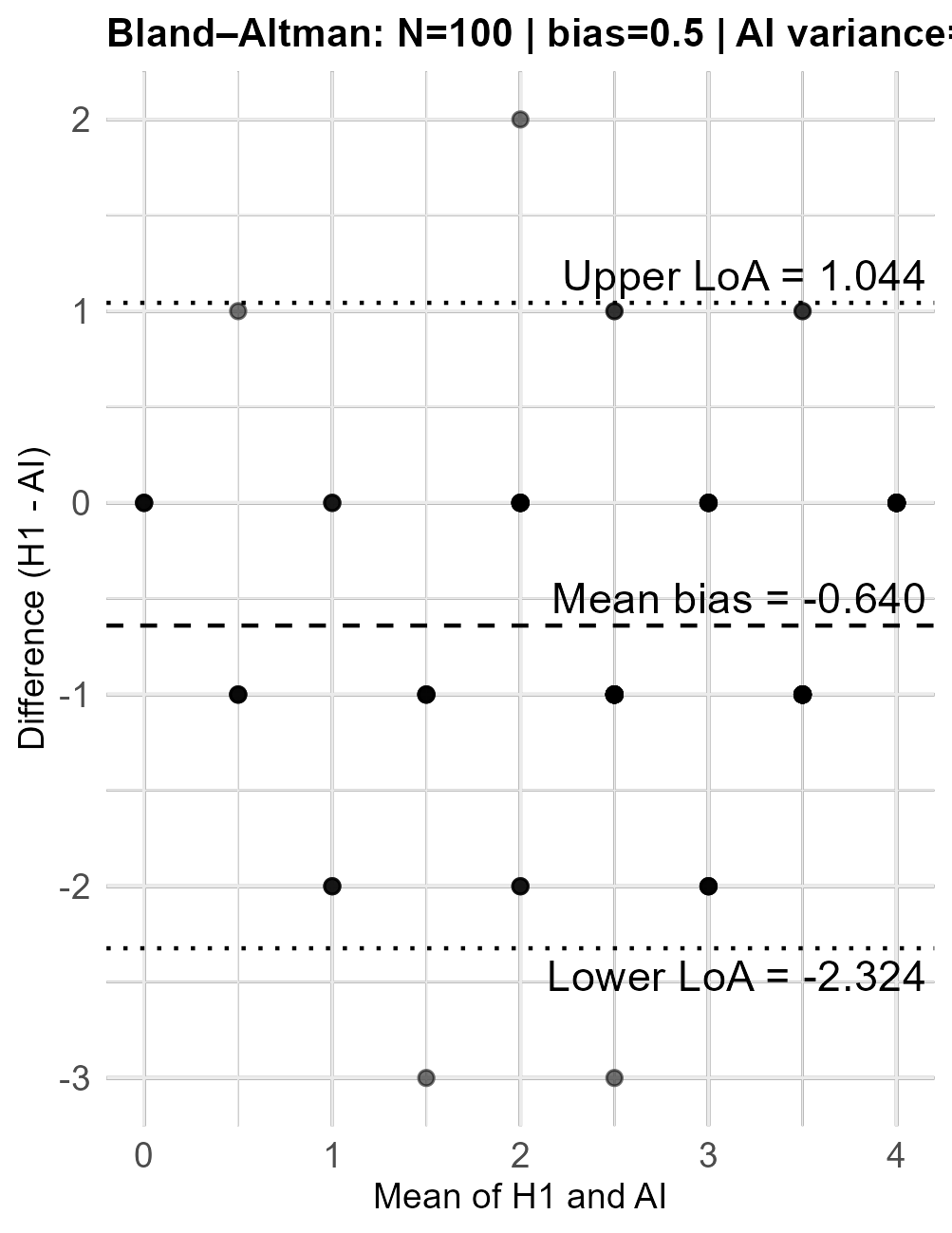

Supplement: Supplementary file 4 [file Data_Sheet_4.zip › BA_N100_bias0.5_ailow_compFALSE_imbTRUE_fairTRUE_rep1.tif]

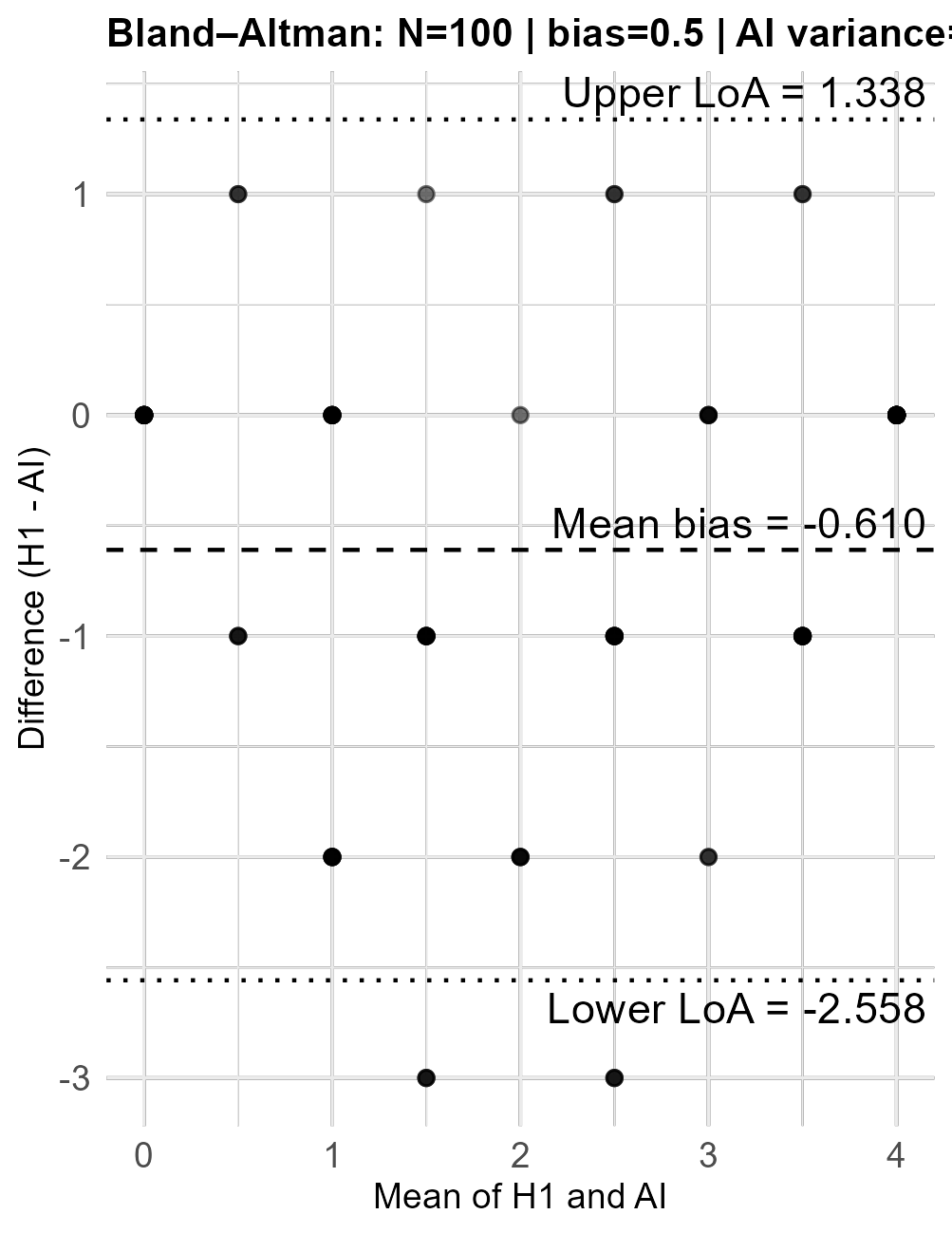

Supplement: Supplementary file 4 [file Data_Sheet_4.zip › BA_N100_bias0.5_ailow_compFALSE_imbFALSE_fairFALSE_rep1.tif]

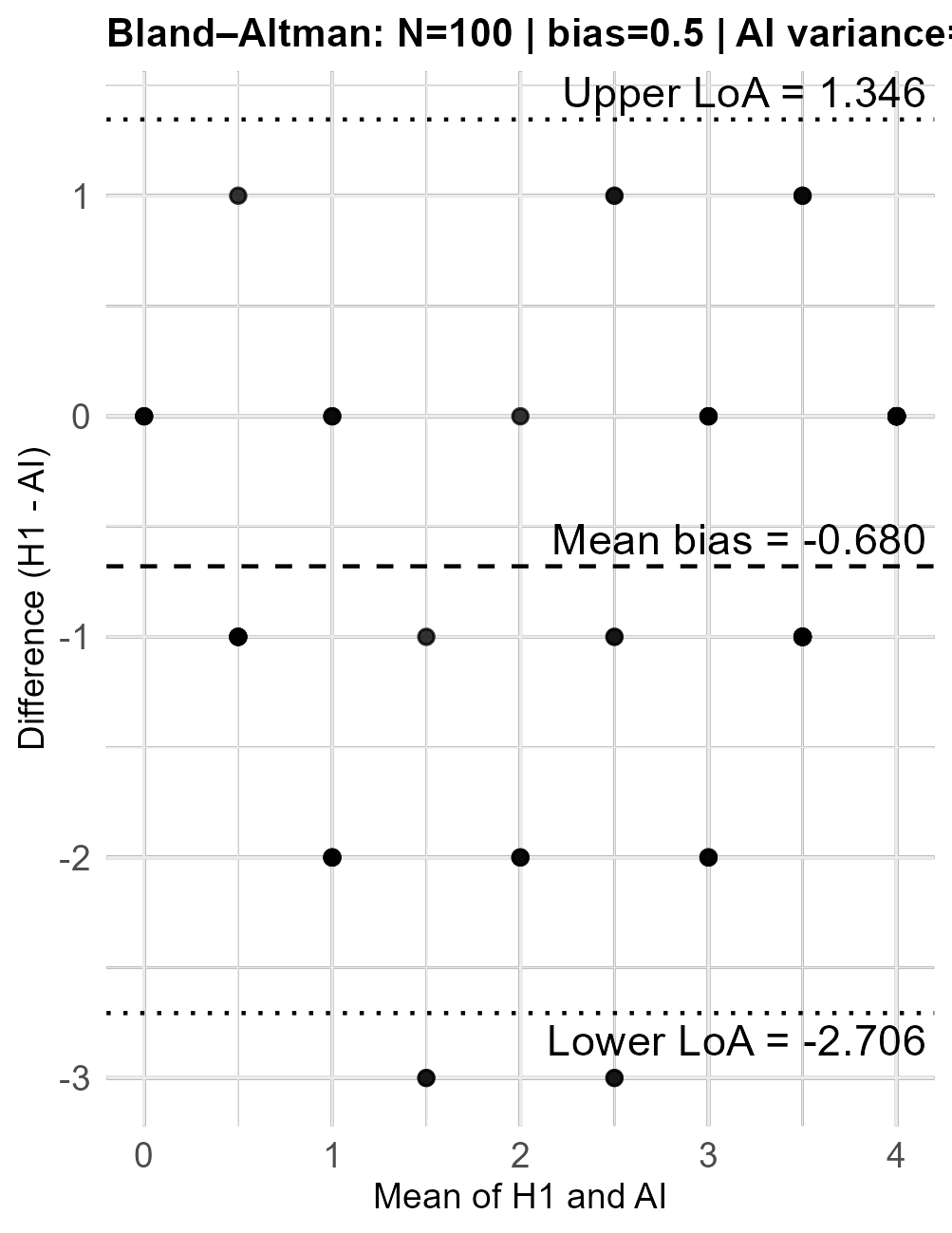

Supplement: Supplementary file 4 [file Data_Sheet_4.zip › BA_N100_bias0.5_ailow_compFALSE_imbFALSE_fairTRUE_rep1.tif]

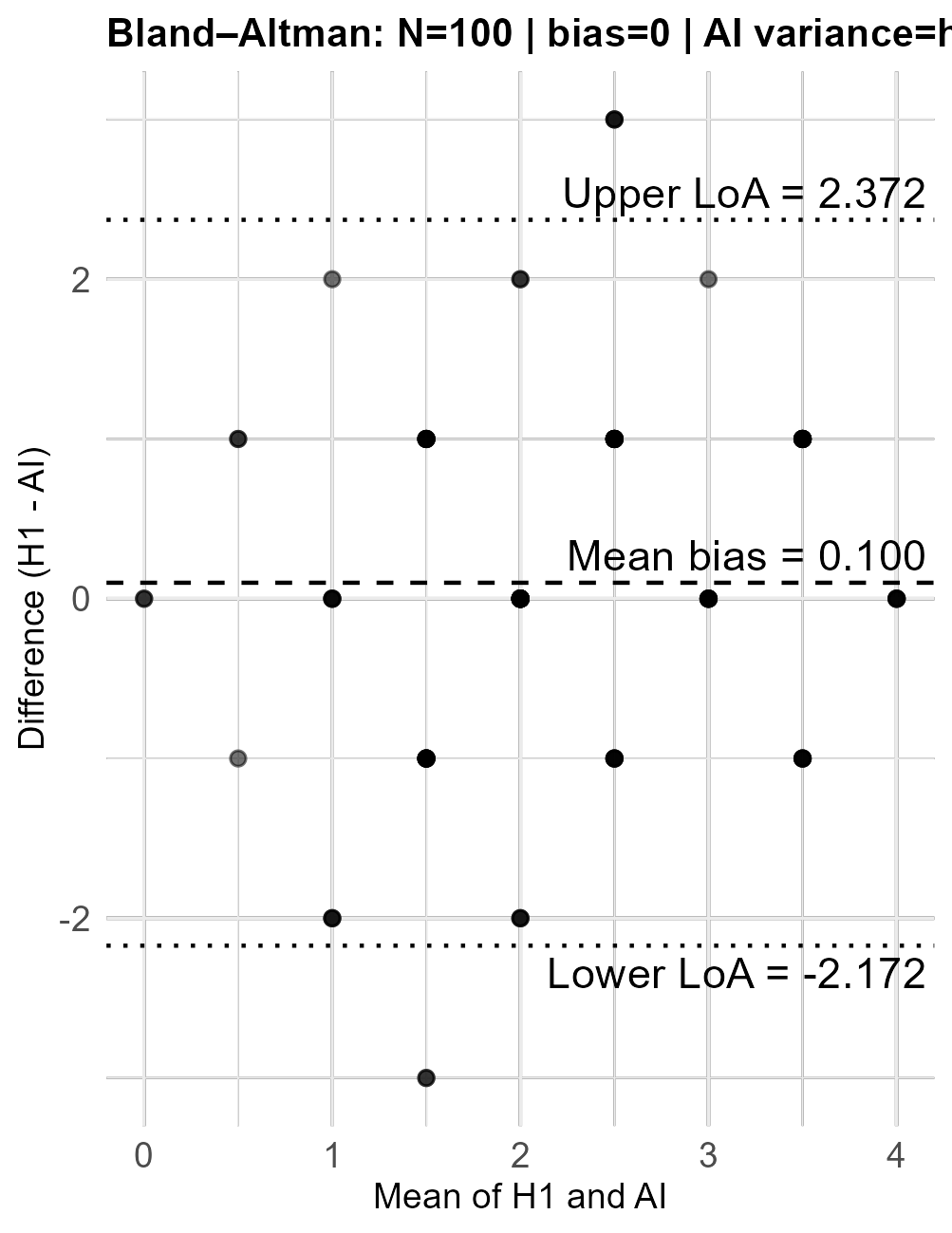

Supplement: Supplementary file 4 [file Data_Sheet_4.zip › BA_N100_bias0_aihigh_compTRUE_imbTRUE_fairFALSE_rep1.tif]

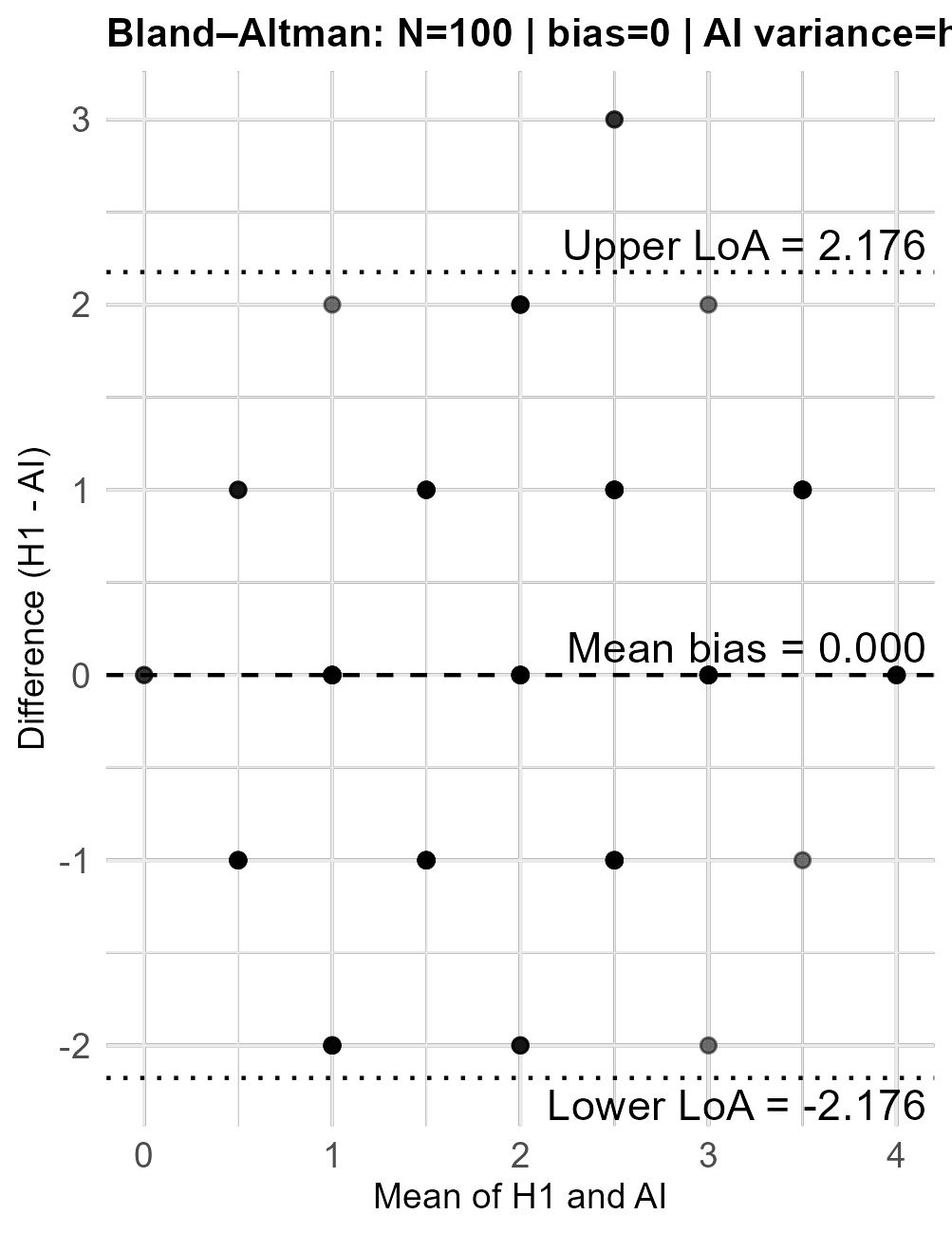

Supplement: Supplementary file 4 [file Data_Sheet_4.zip › BA_N100_bias0_aihigh_compTRUE_imbTRUE_fairTRUE_rep1.tif]

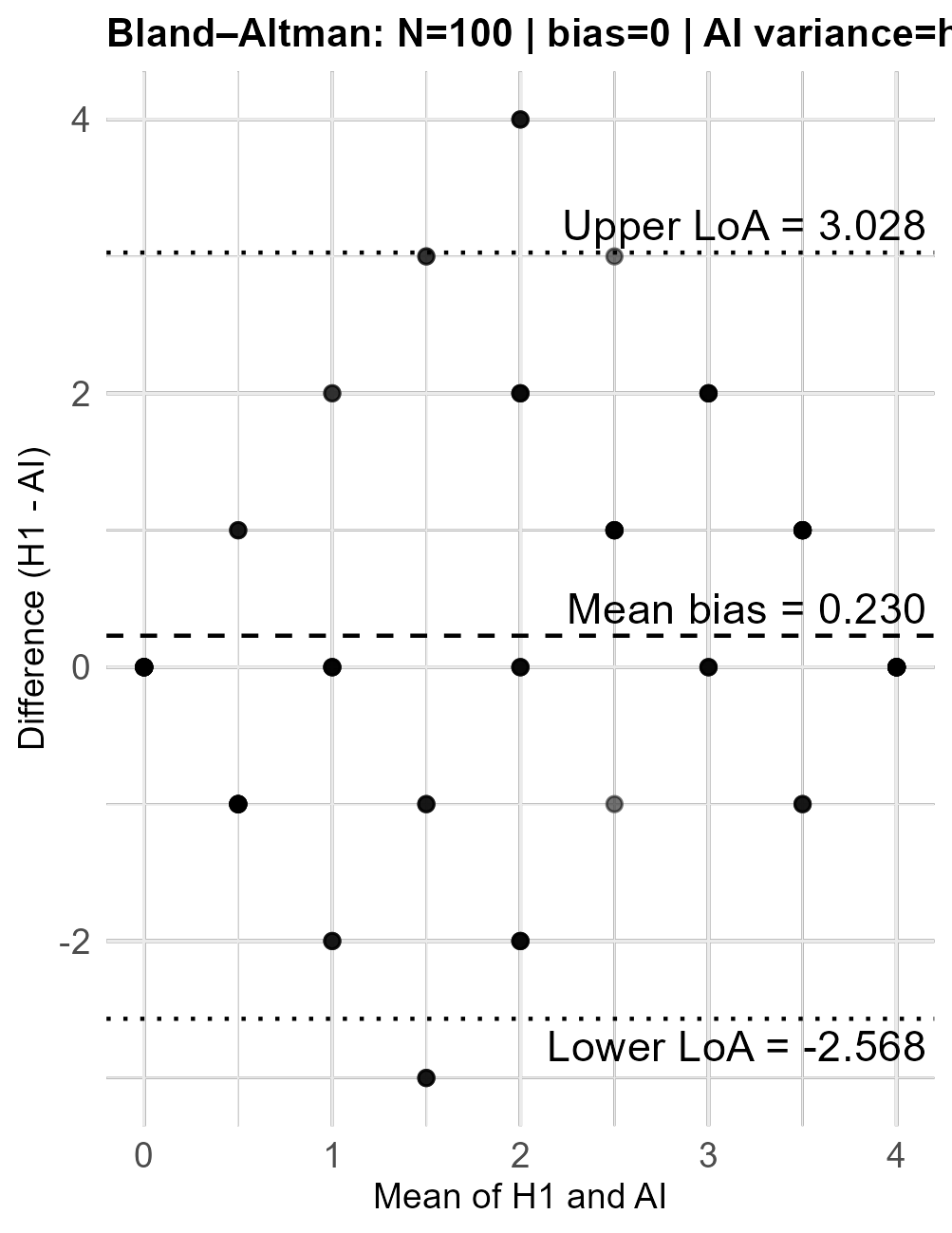

Supplement: Supplementary file 4 [file Data_Sheet_4.zip › BA_N100_bias0_aihigh_compTRUE_imbFALSE_fairFALSE_rep1.tif]

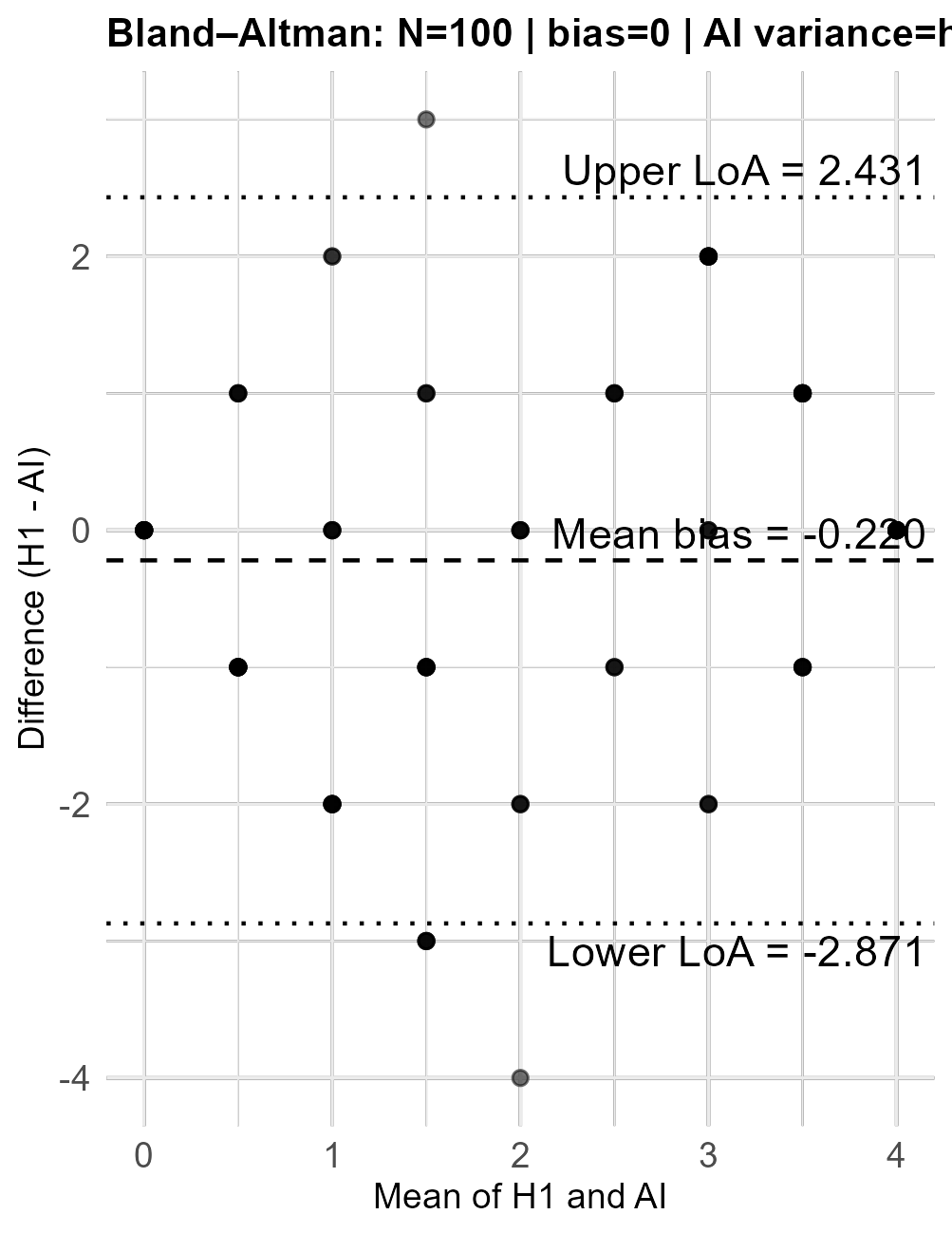

Supplement: Supplementary file 4 [file Data_Sheet_4.zip › BA_N100_bias0_aihigh_compTRUE_imbFALSE_fairTRUE_rep1.tif]

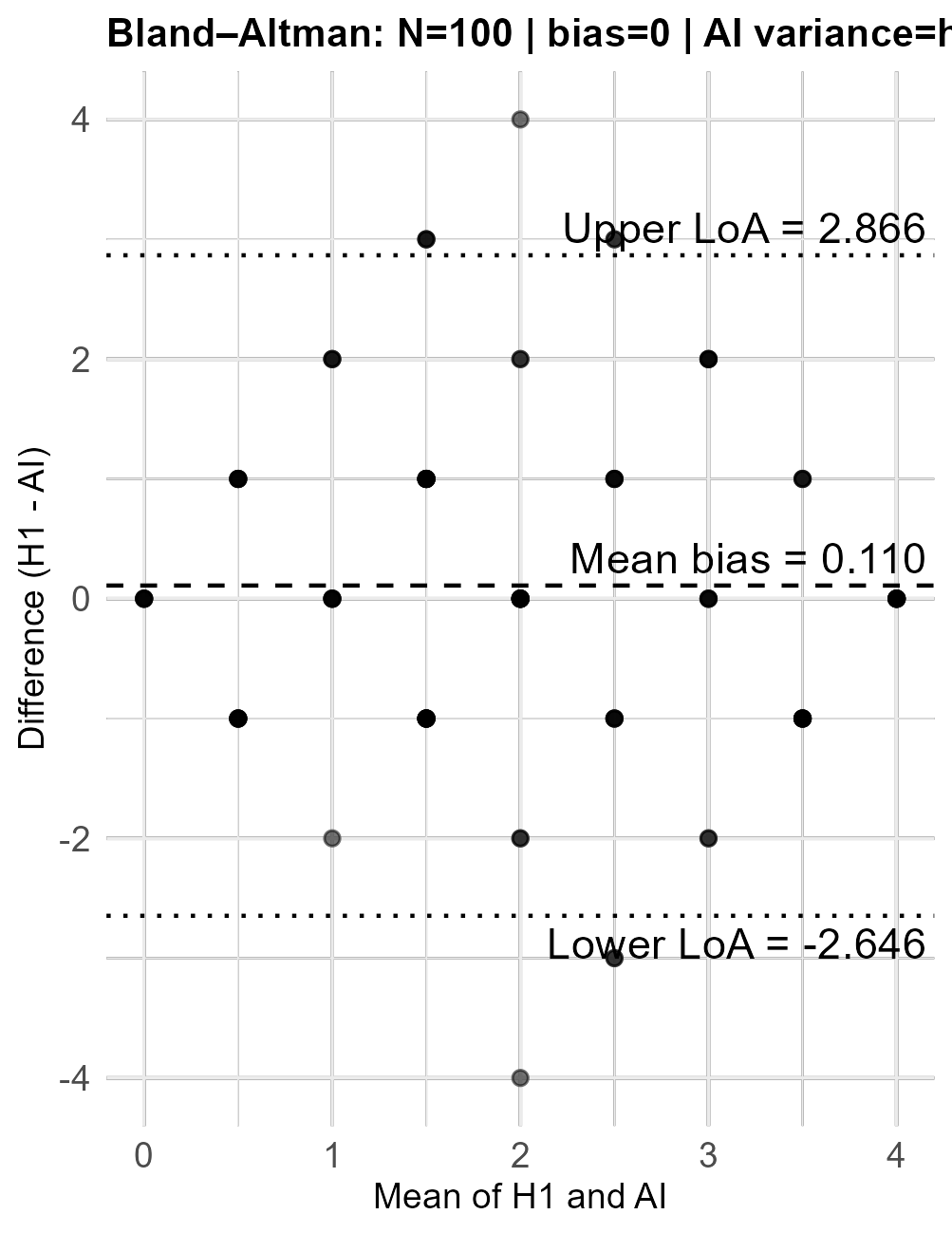

Supplement: Supplementary file 4 [file Data_Sheet_4.zip › BA_N100_bias0_aihigh_compFALSE_imbTRUE_fairFALSE_rep1.tif]

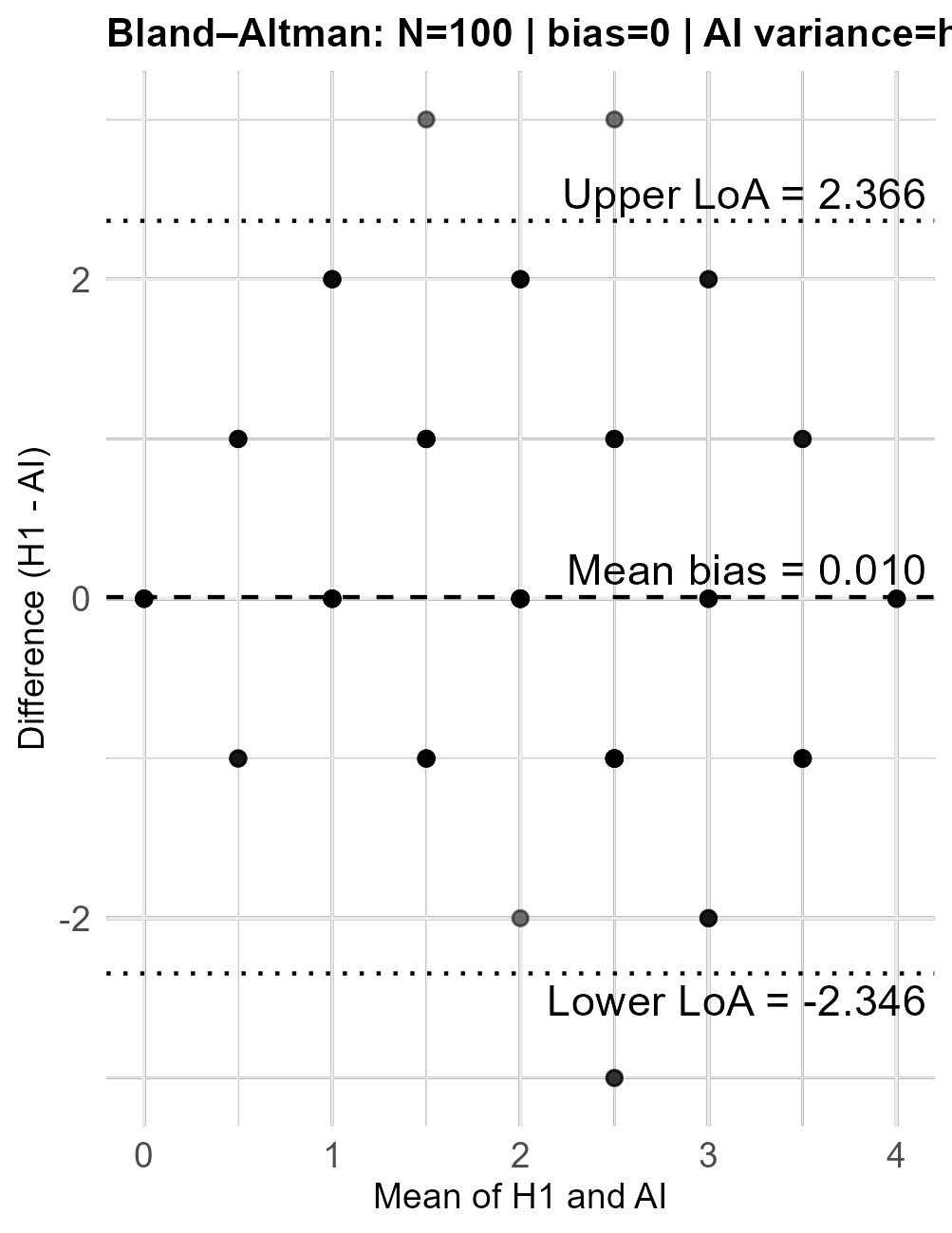

Supplement: Supplementary file 4 [file Data_Sheet_4.zip › BA_N100_bias0_aihigh_compFALSE_imbTRUE_fairTRUE_rep1.tif]

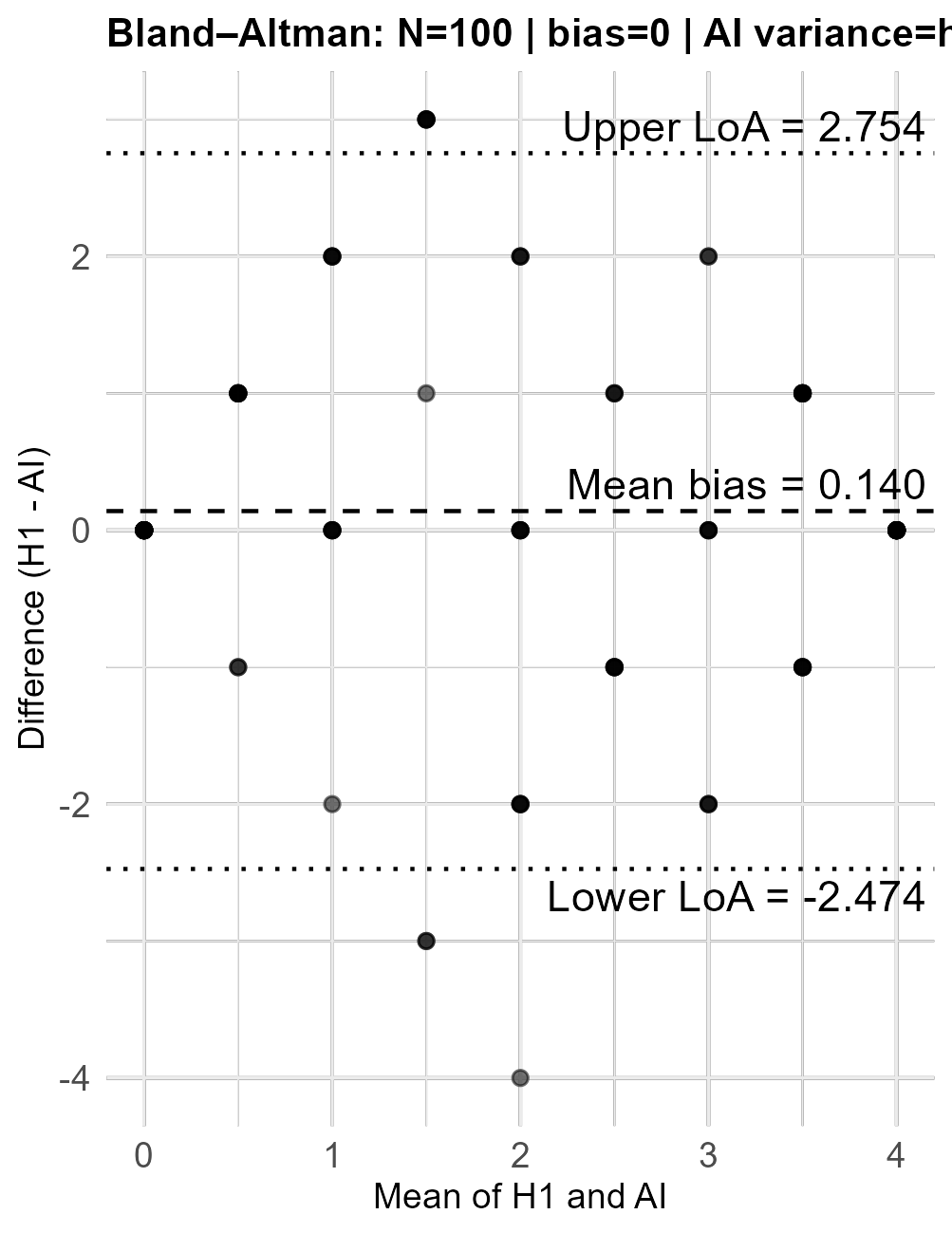

Supplement: Supplementary file 4 [file Data_Sheet_4.zip › BA_N100_bias0_aihigh_compFALSE_imbFALSE_fairFALSE_rep1.tif]

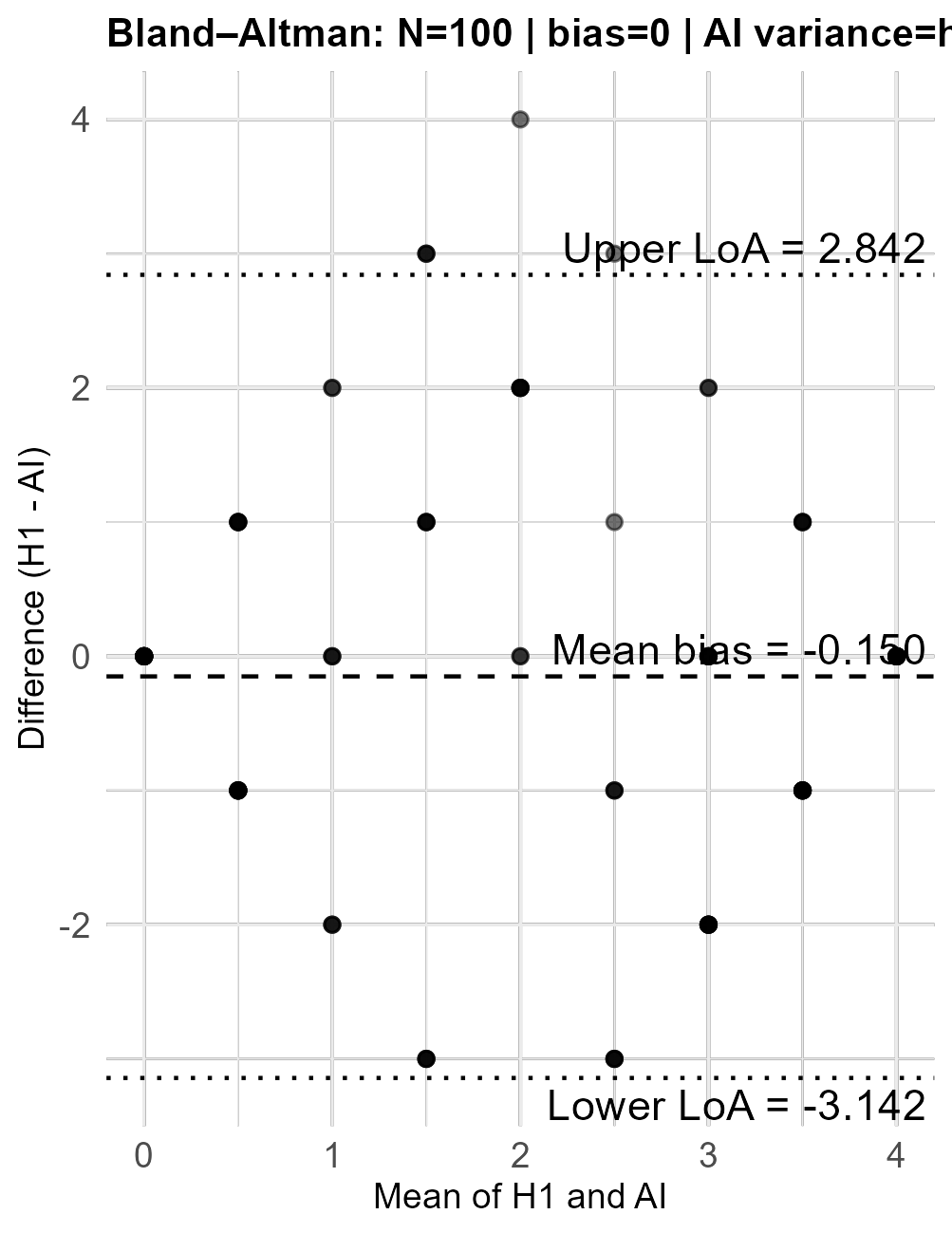

Supplement: Supplementary file 4 [file Data_Sheet_4.zip › BA_N100_bias0_aihigh_compFALSE_imbFALSE_fairTRUE_rep1.tif]

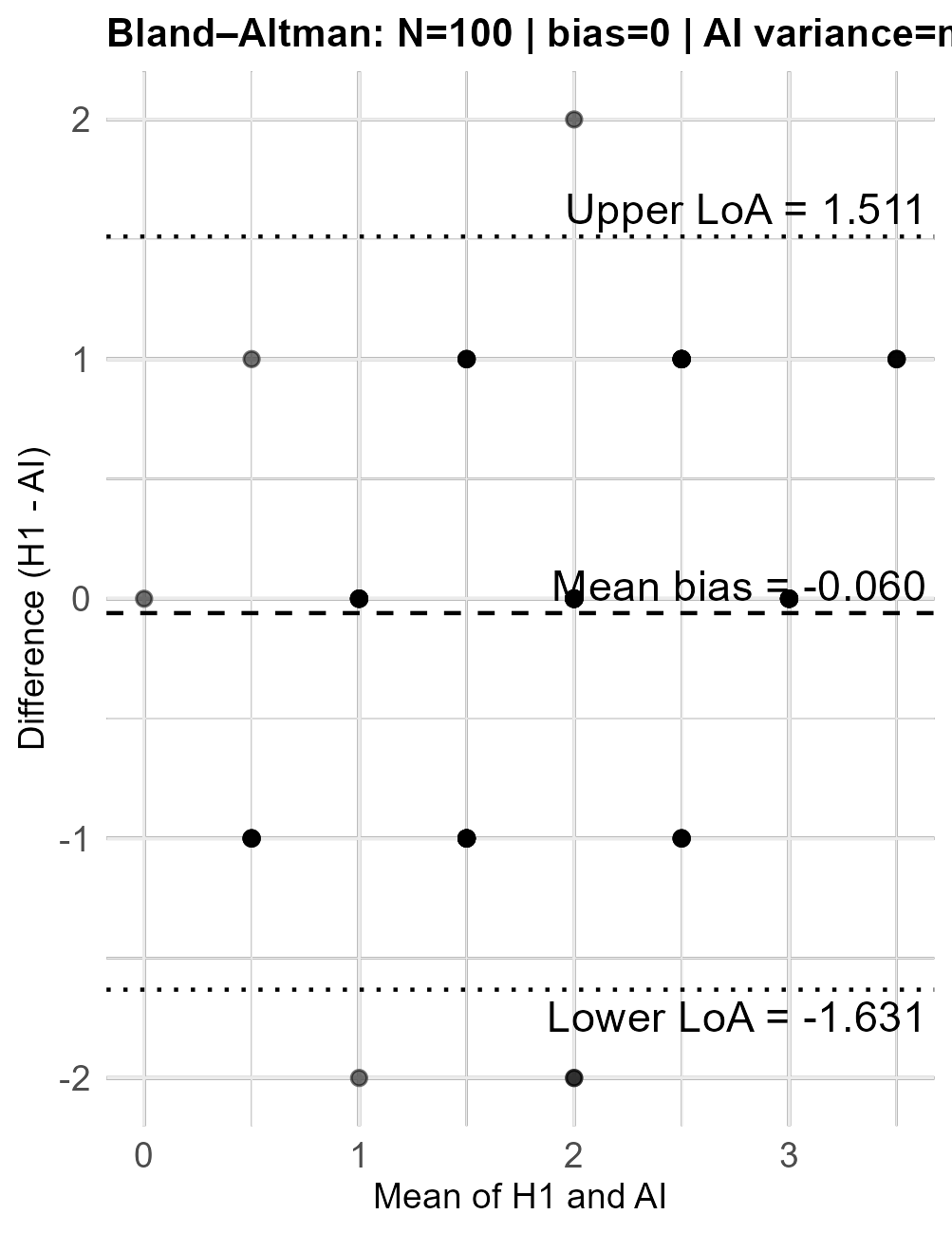

Supplement: Supplementary file 4 [file Data_Sheet_4.zip › BA_N100_bias0_aimid_compTRUE_imbTRUE_fairFALSE_rep1.tif]

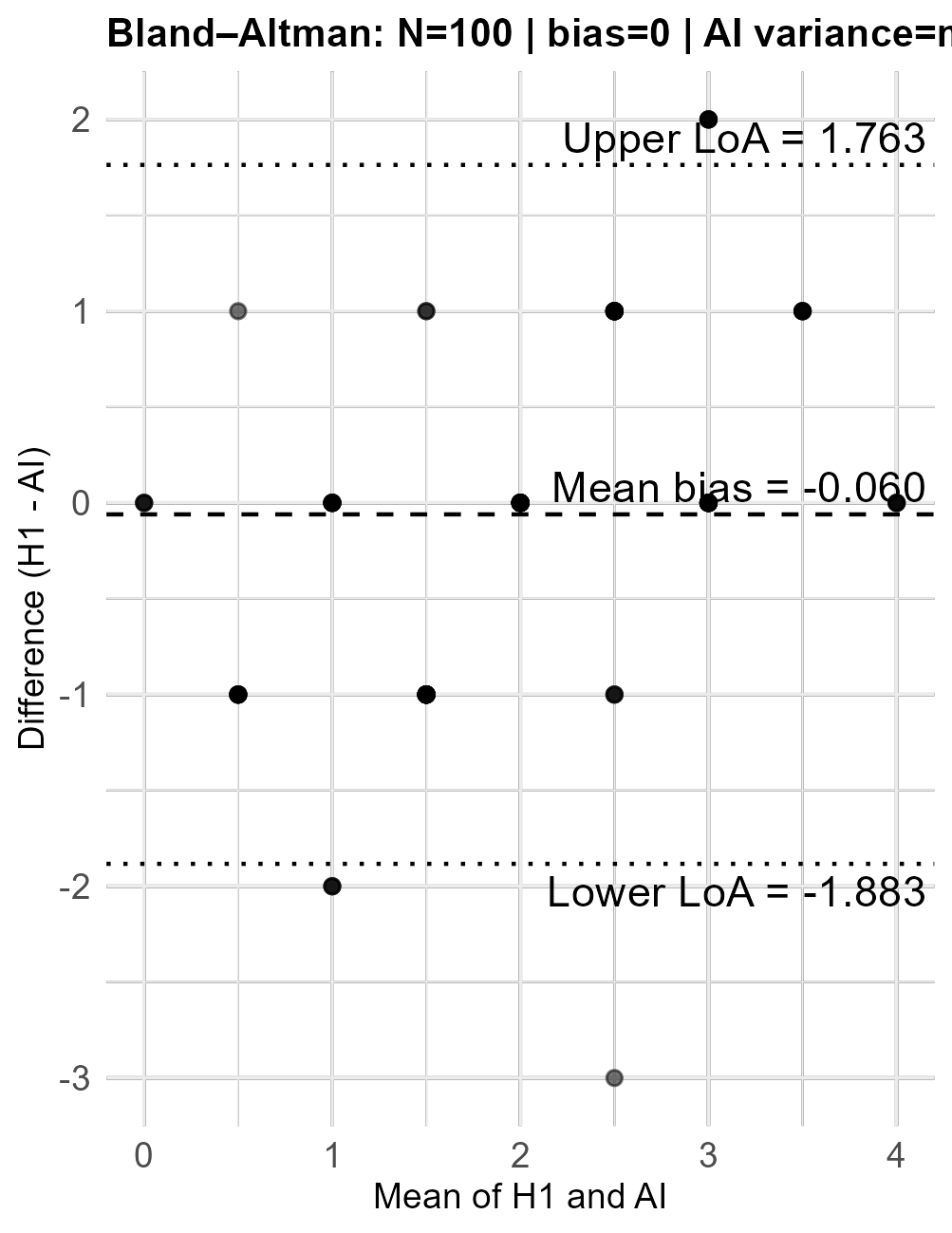

Supplement: Supplementary file 4 [file Data_Sheet_4.zip › BA_N100_bias0_aimid_compTRUE_imbTRUE_fairTRUE_rep1.tif]

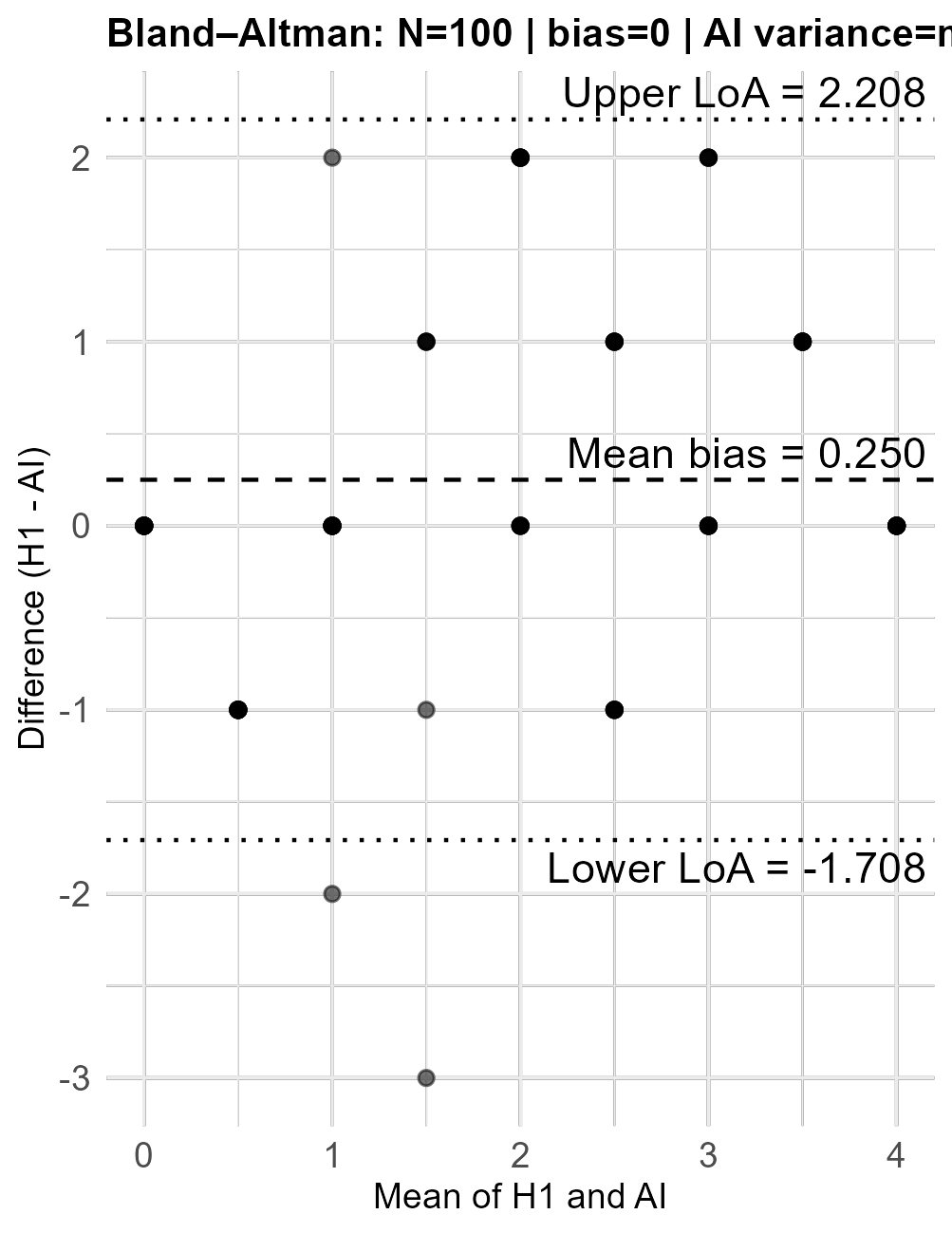

Supplement: Supplementary file 4 [file Data_Sheet_4.zip › BA_N100_bias0_aimid_compTRUE_imbFALSE_fairFALSE_rep1.tif]

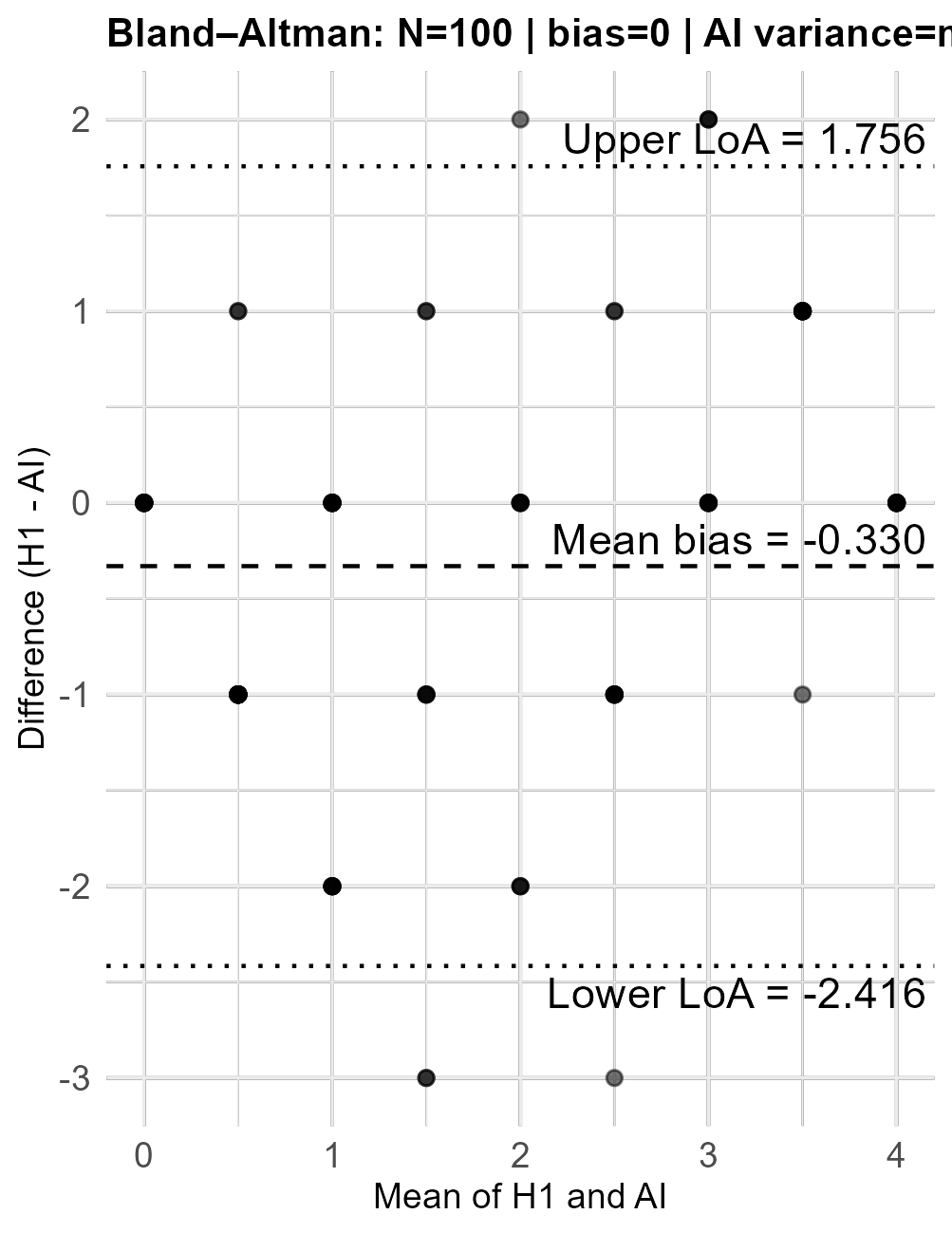

Supplement: Supplementary file 4 [file Data_Sheet_4.zip › BA_N100_bias0_aimid_compTRUE_imbFALSE_fairTRUE_rep1.tif]

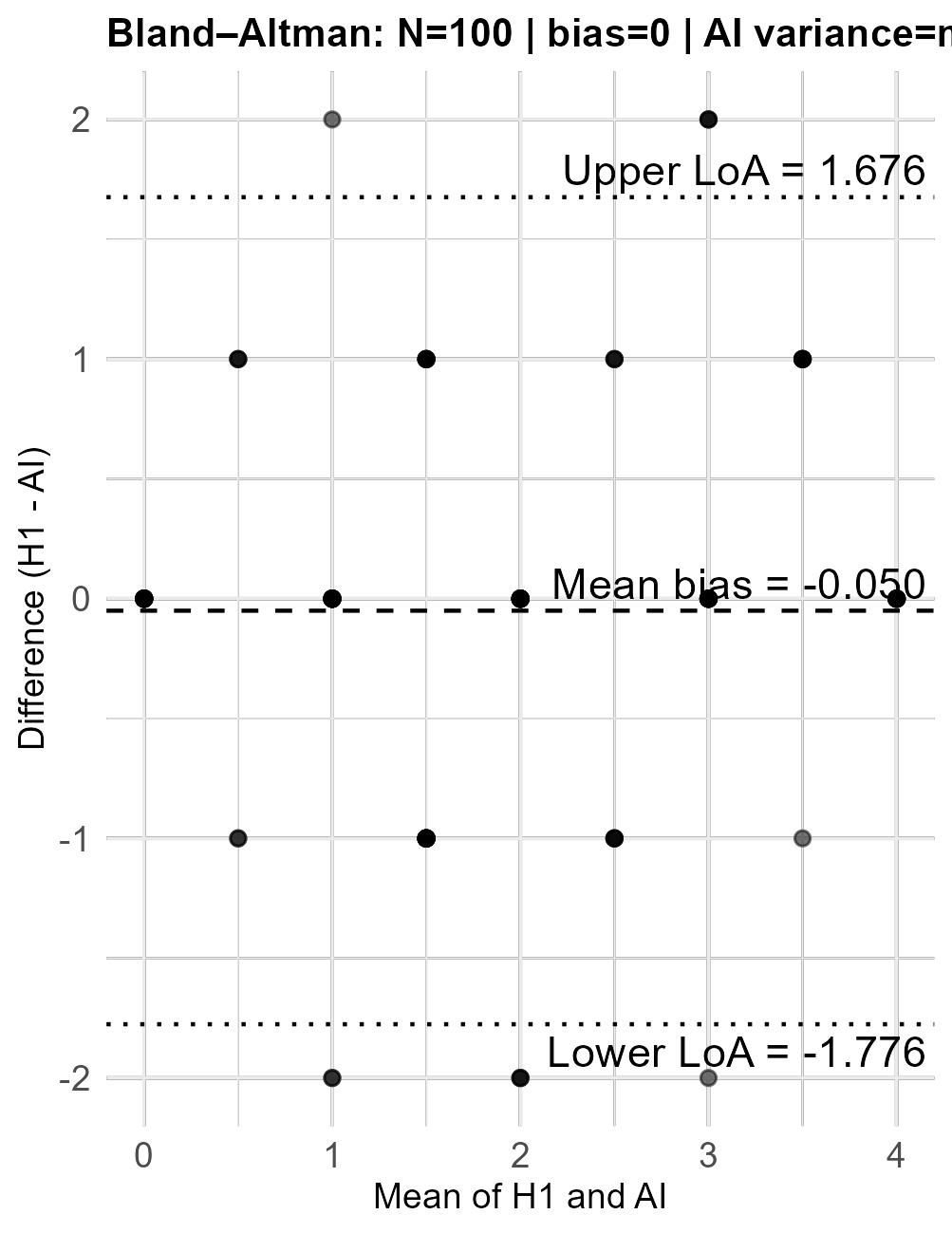

Supplement: Supplementary file 4 [file Data_Sheet_4.zip › BA_N100_bias0_aimid_compFALSE_imbTRUE_fairFALSE_rep1.tif]

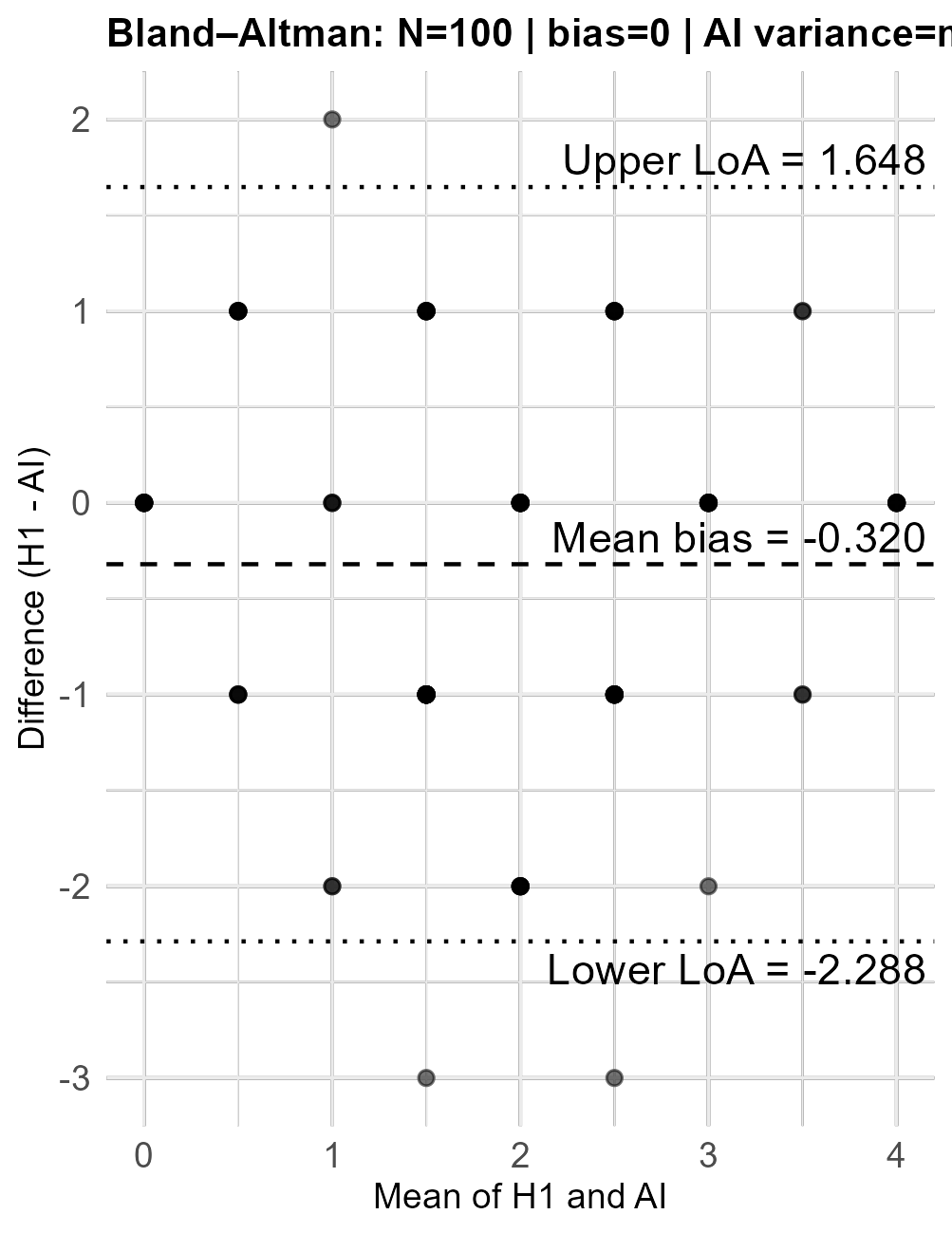

Supplement: Supplementary file 4 [file Data_Sheet_4.zip › BA_N100_bias0_aimid_compFALSE_imbTRUE_fairTRUE_rep1.tif]

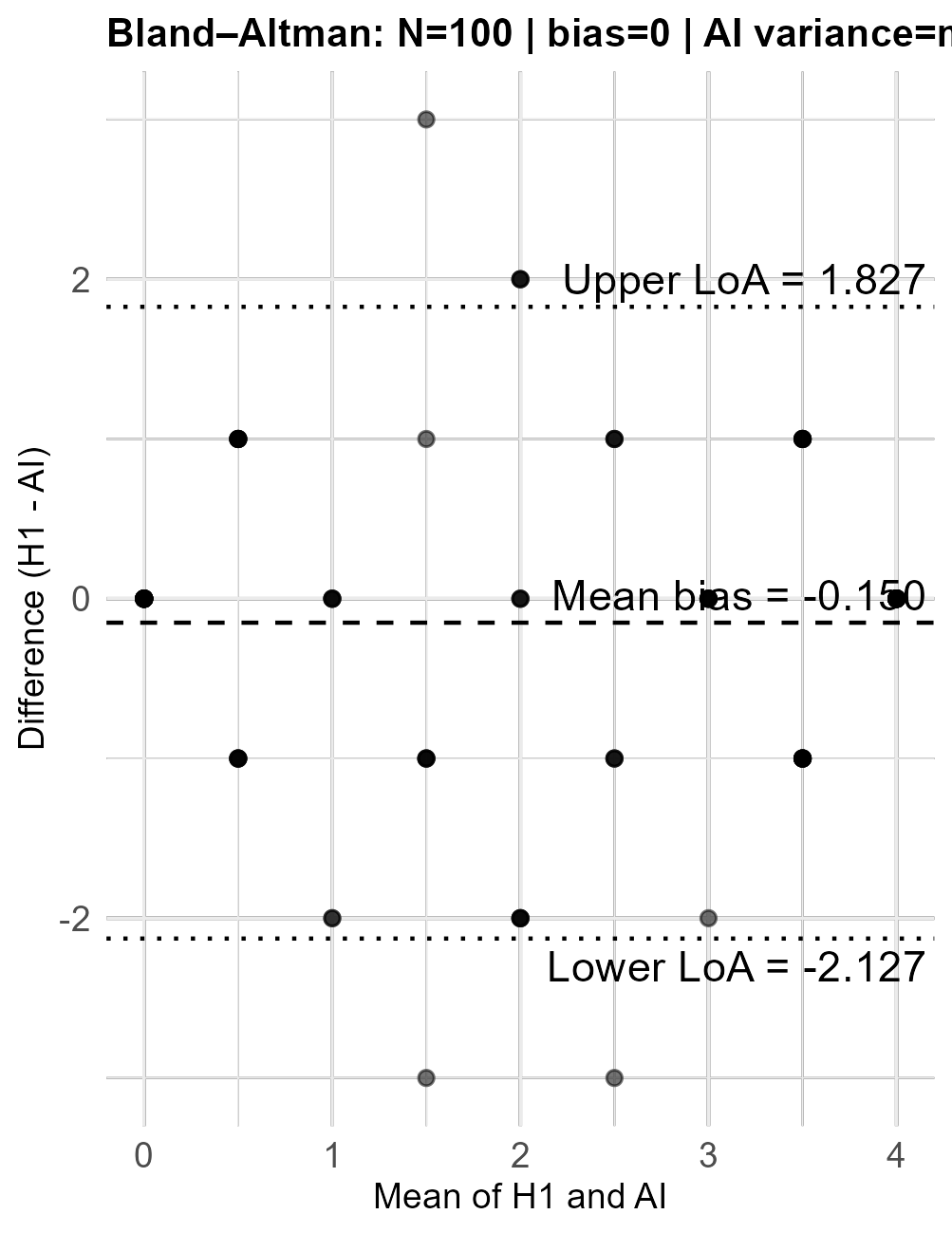

Supplement: Supplementary file 4 [file Data_Sheet_4.zip › BA_N100_bias0_aimid_compFALSE_imbFALSE_fairFALSE_rep1.tif]

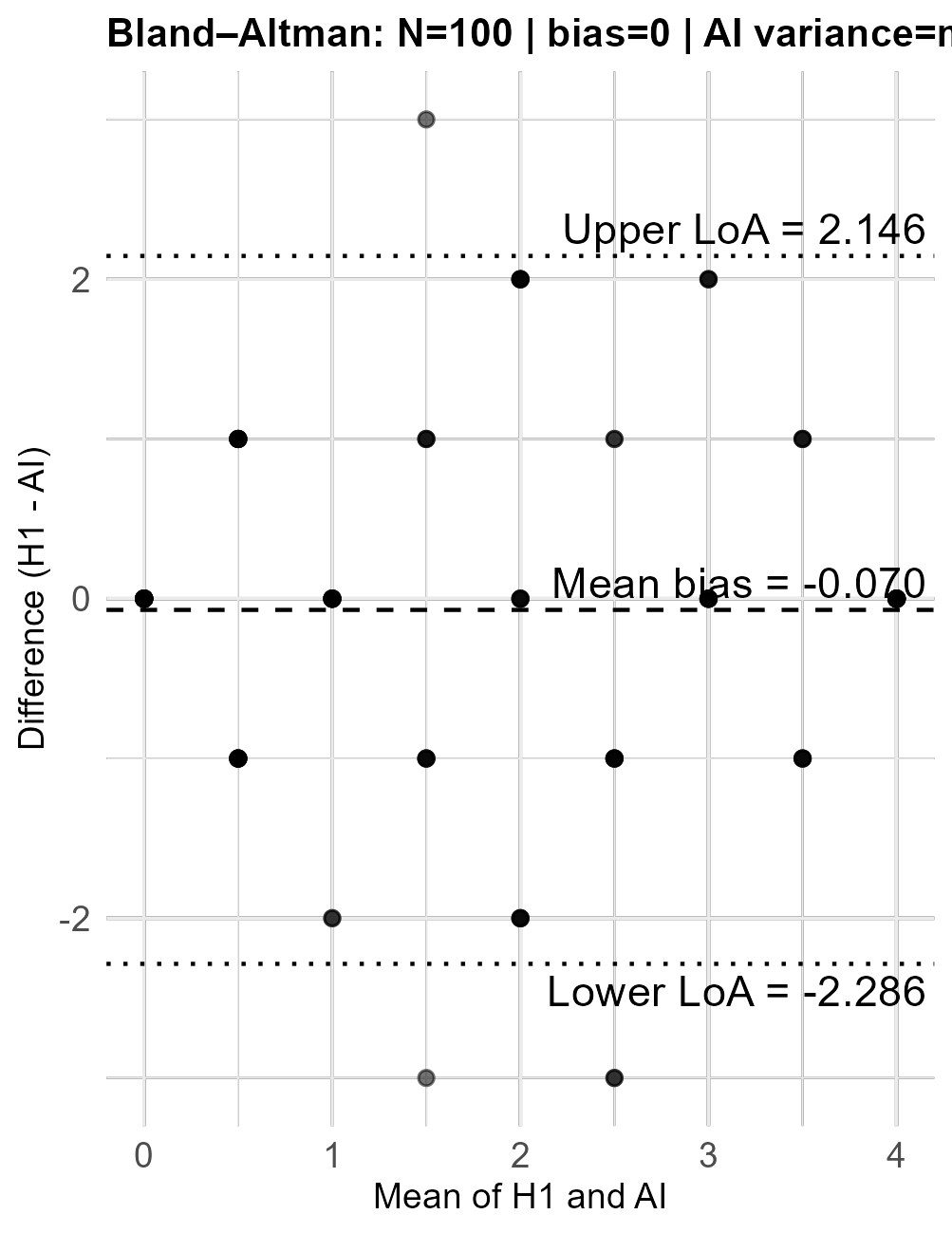

Supplement: Supplementary file 4 [file Data_Sheet_4.zip › BA_N100_bias0_aimid_compFALSE_imbFALSE_fairTRUE_rep1.tif]

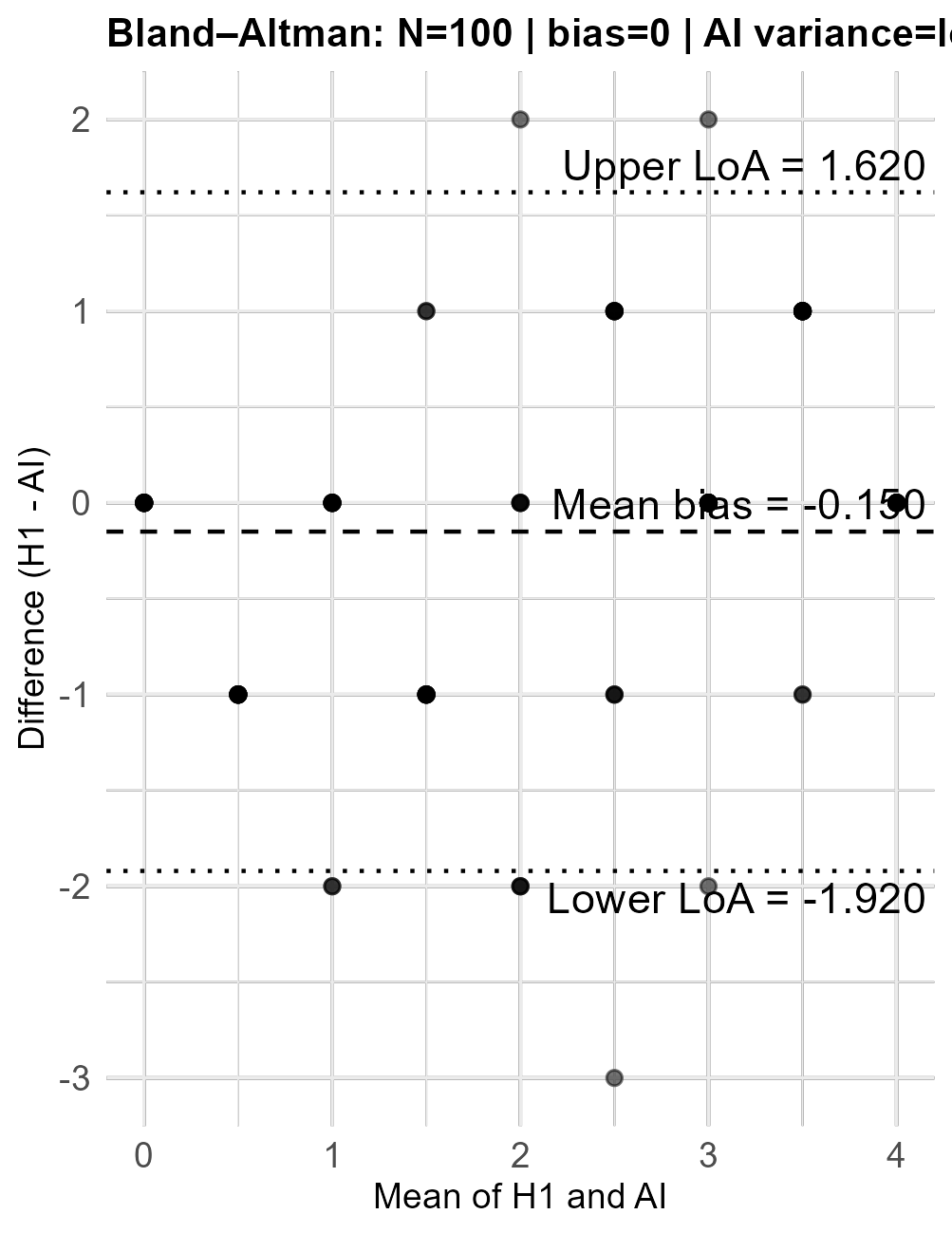

Supplement: Supplementary file 4 [file Data_Sheet_4.zip › BA_N100_bias0_ailow_compTRUE_imbFALSE_fairTRUE_rep1.tif]

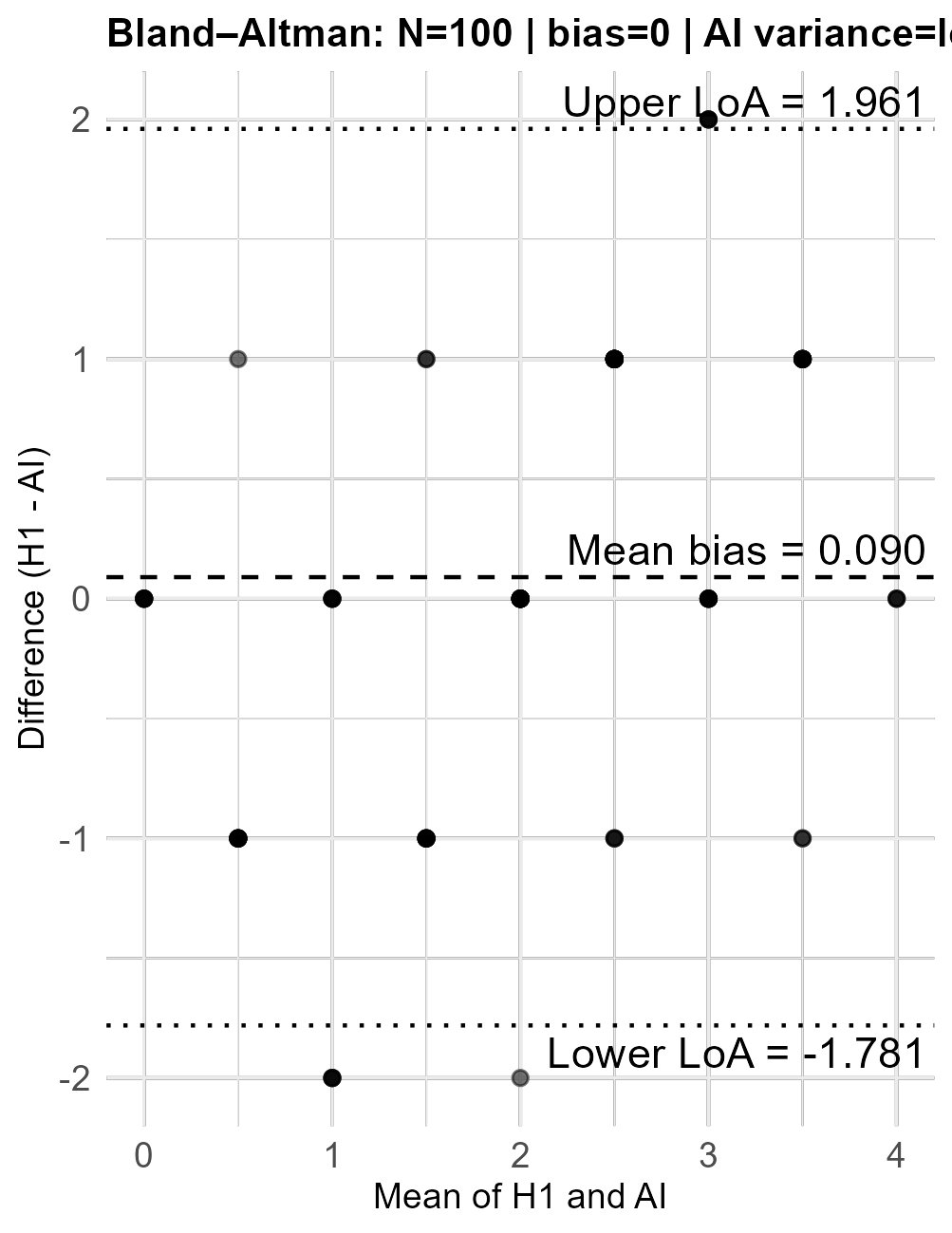

Supplement: Supplementary file 4 [file Data_Sheet_4.zip › BA_N100_bias0_ailow_compTRUE_imbTRUE_fairFALSE_rep1.tif]

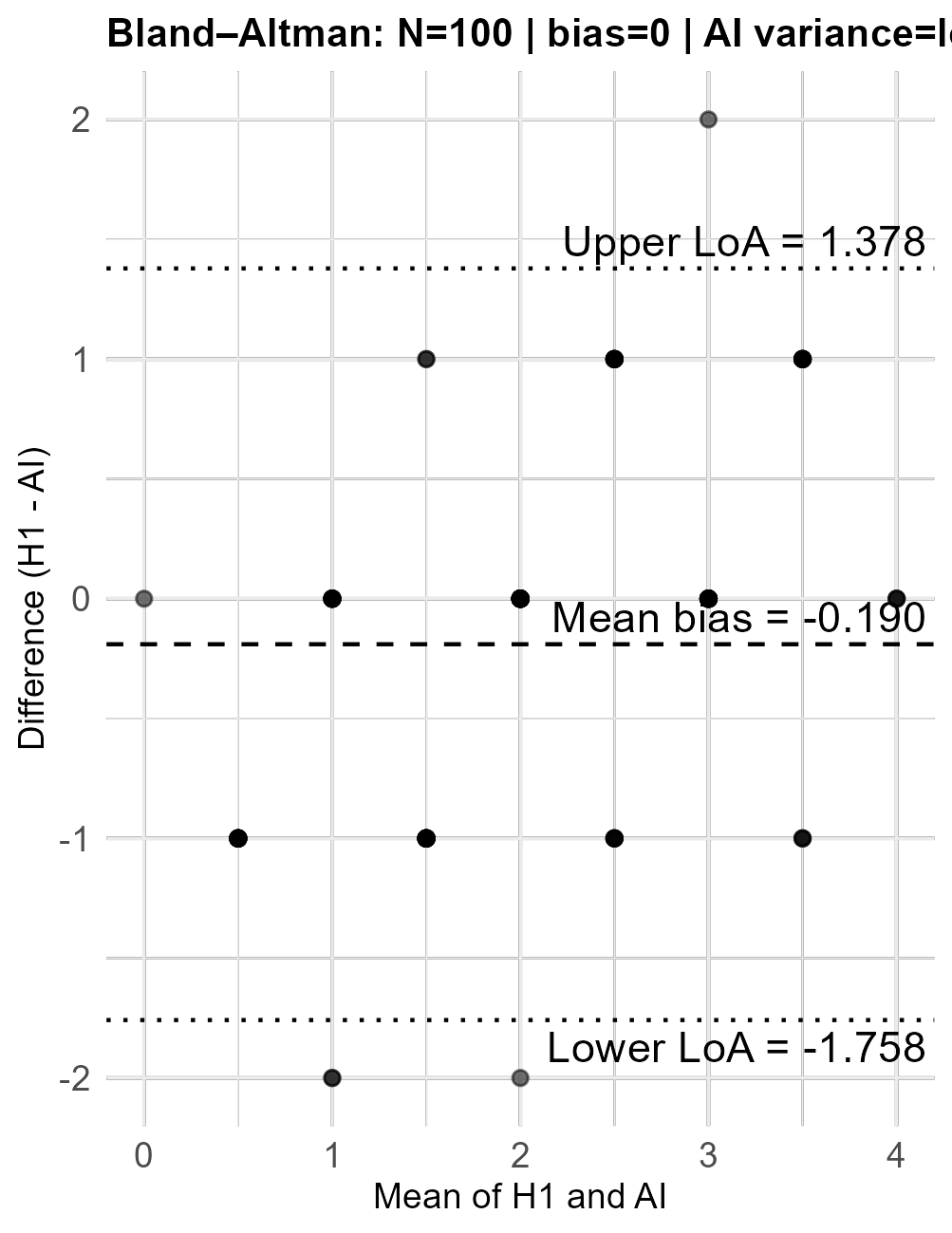

Supplement: Supplementary file 4 [file Data_Sheet_4.zip › BA_N100_bias0_ailow_compTRUE_imbTRUE_fairTRUE_rep1.tif]

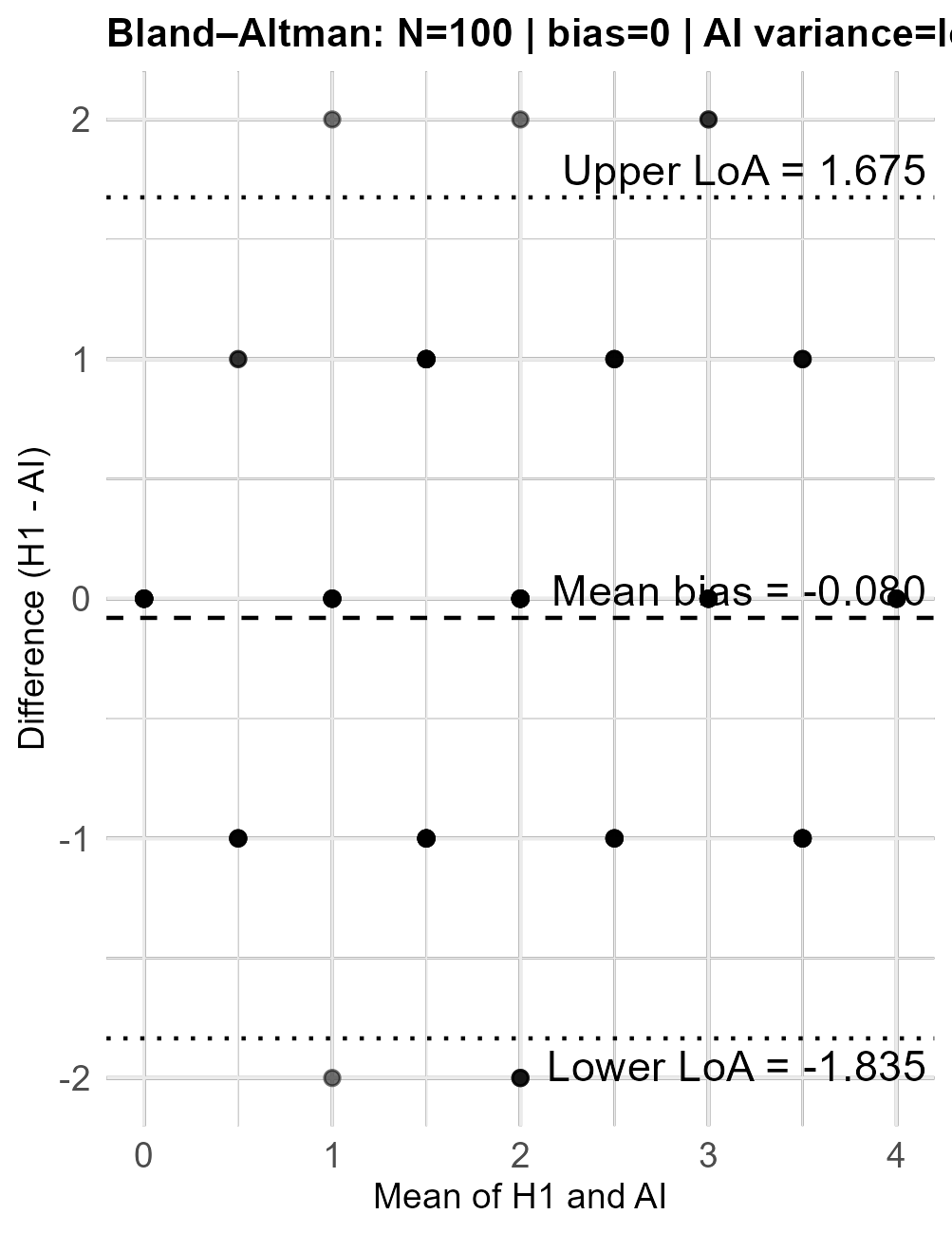

Supplement: Supplementary file 4 [file Data_Sheet_4.zip › BA_N100_bias0_ailow_compFALSE_imbTRUE_fairTRUE_rep1.tif]

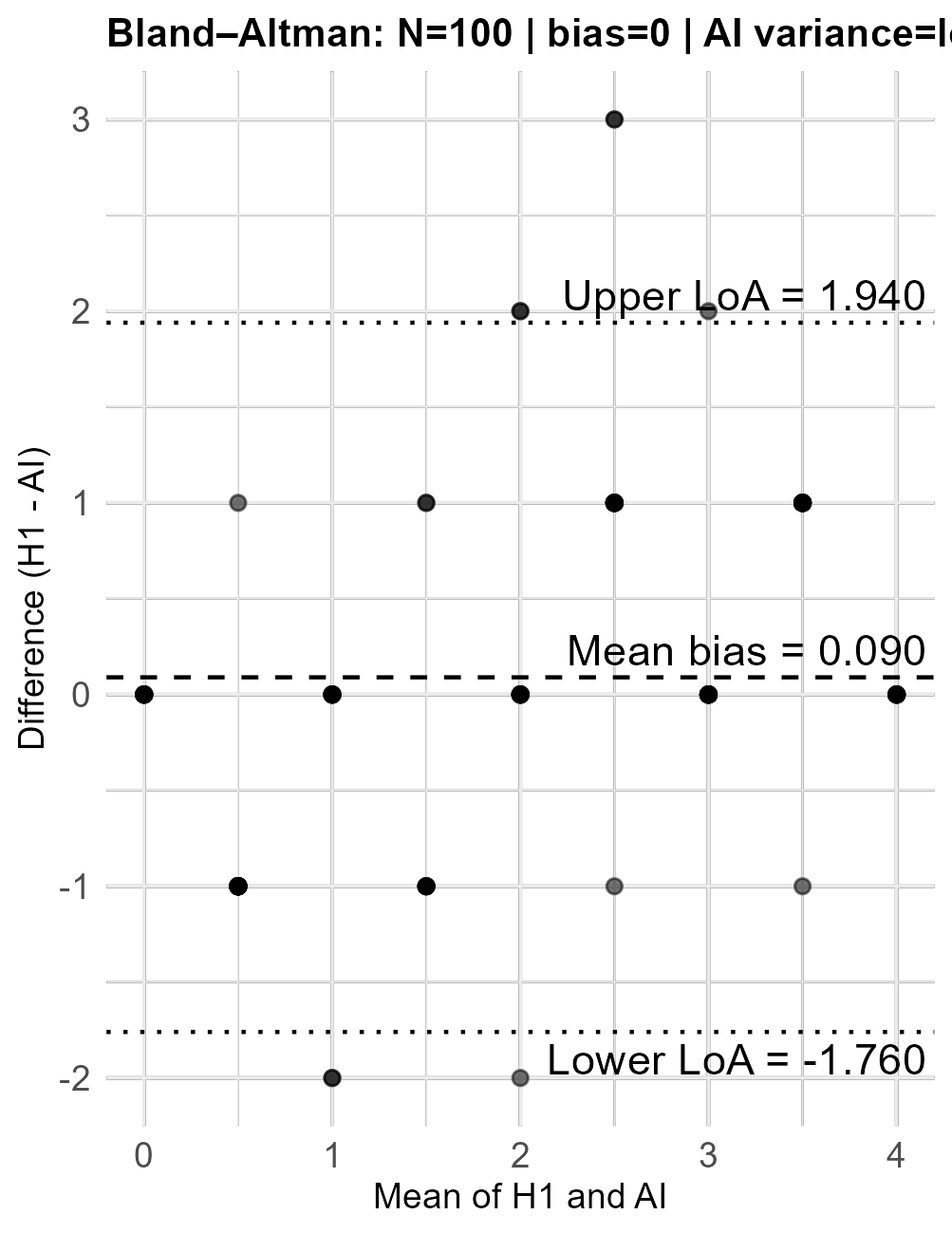

Supplement: Supplementary file 4 [file Data_Sheet_4.zip › BA_N100_bias0_ailow_compTRUE_imbFALSE_fairFALSE_rep1.tif]

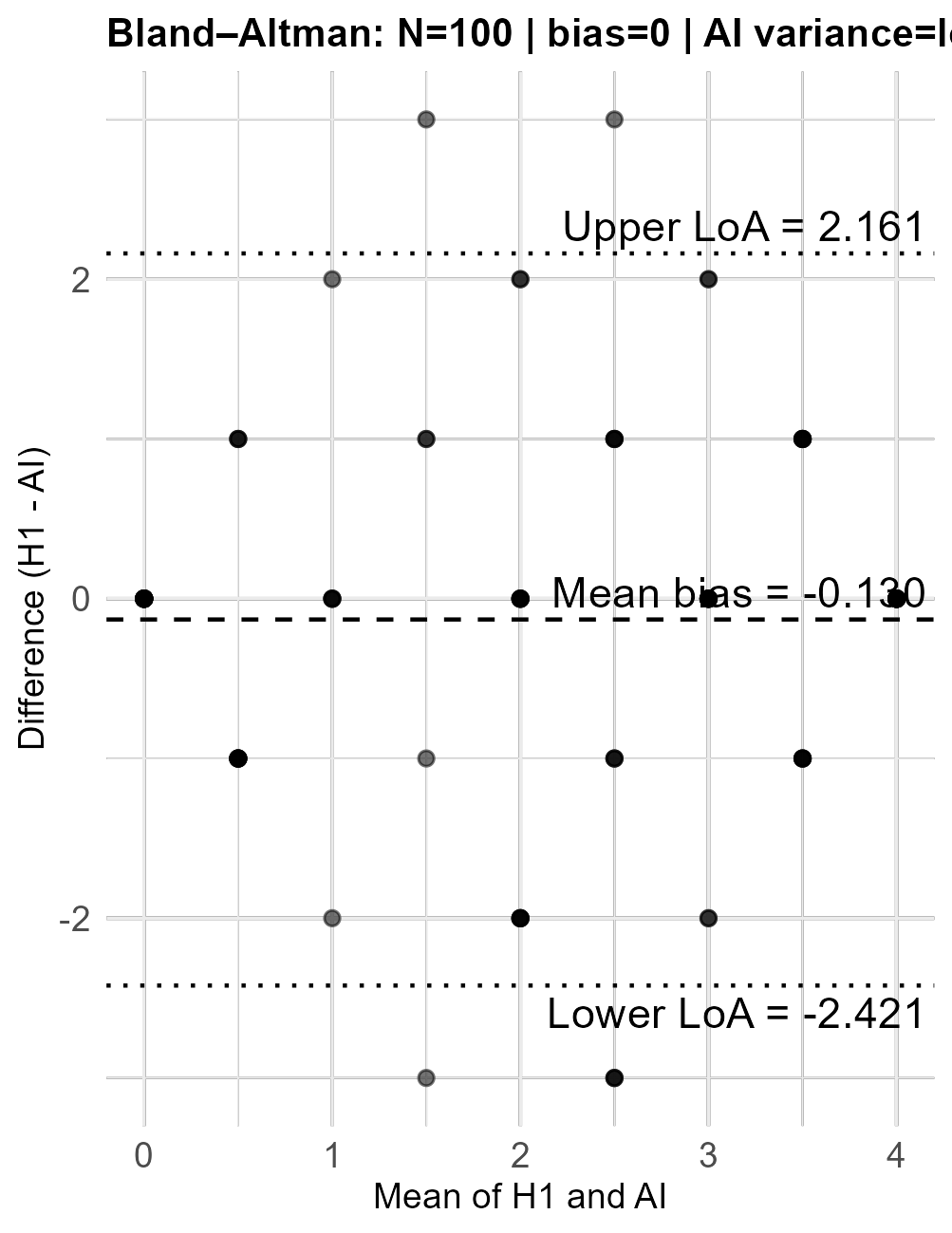

Supplement: Supplementary file 4 [file Data_Sheet_4.zip › BA_N100_bias0_ailow_compFALSE_imbFALSE_fairTRUE_rep1.tif]

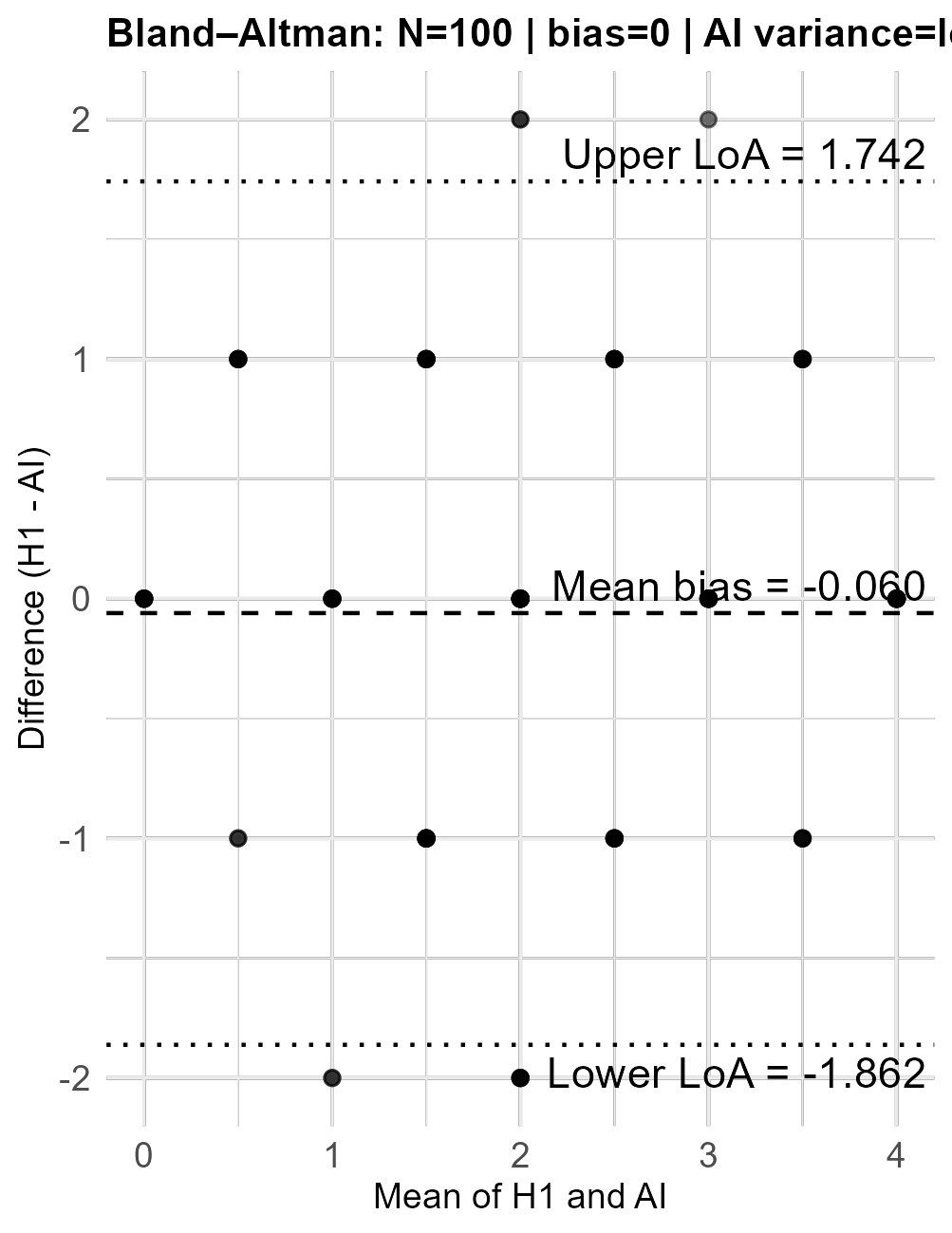

Supplement: Supplementary file 4 [file Data_Sheet_4.zip › BA_N100_bias0_ailow_compFALSE_imbTRUE_fairFALSE_rep1.tif]

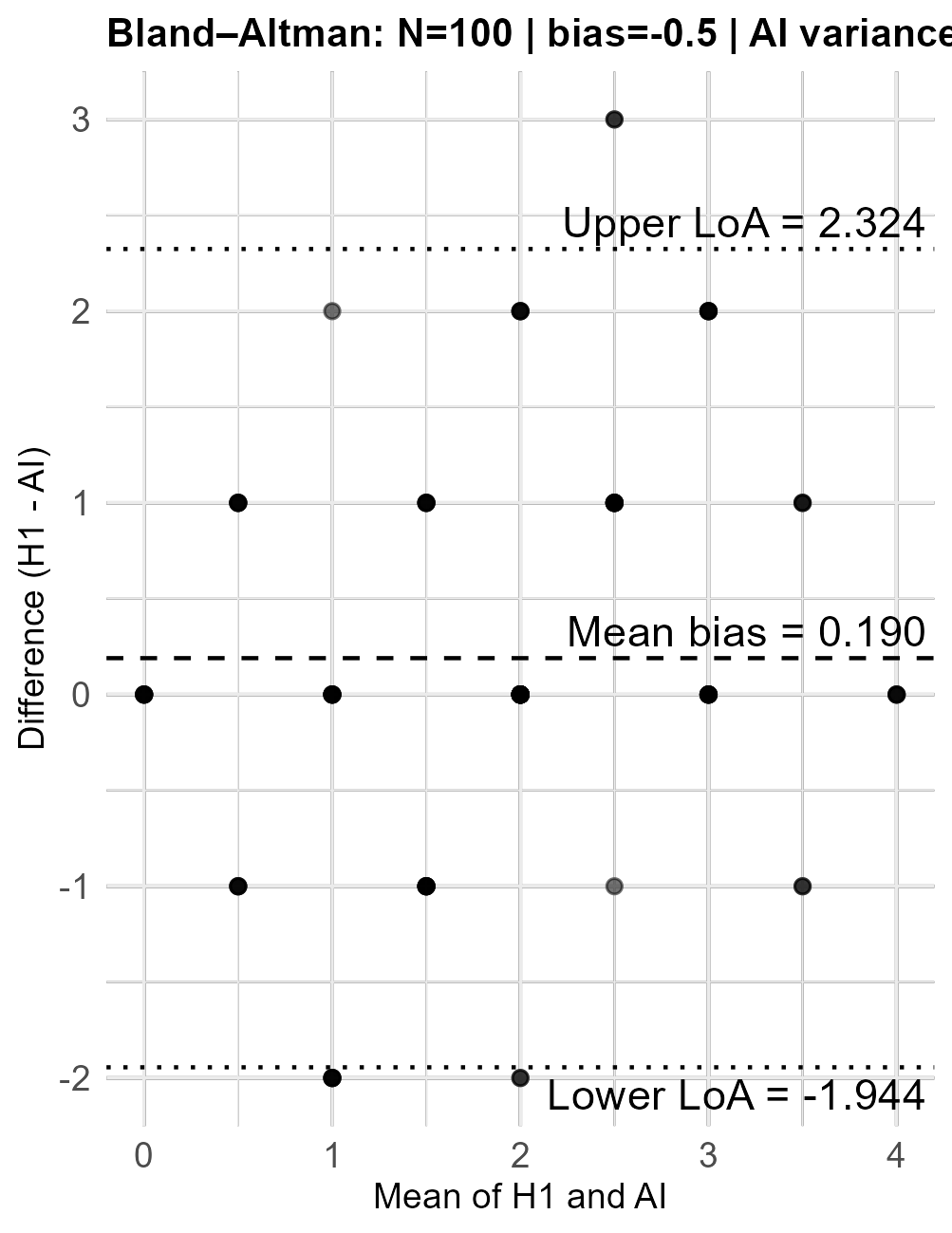

Supplement: Supplementary file 4 [file Data_Sheet_4.zip › BA_N100_bias-0.5_aihigh_compTRUE_imbTRUE_fairTRUE_rep1.tif]

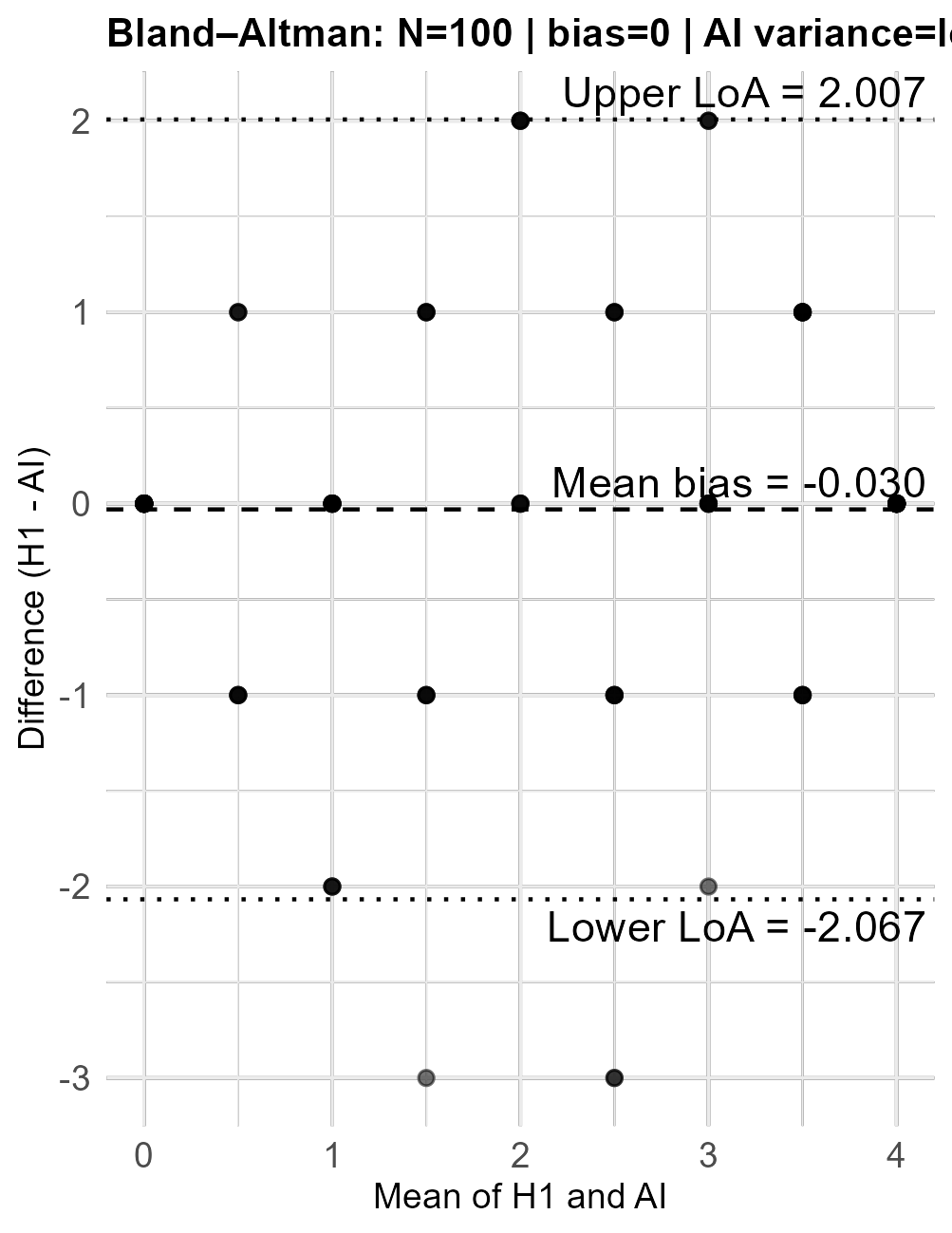

Supplement: Supplementary file 4 [file Data_Sheet_4.zip › BA_N100_bias0_ailow_compFALSE_imbFALSE_fairFALSE_rep1.tif]

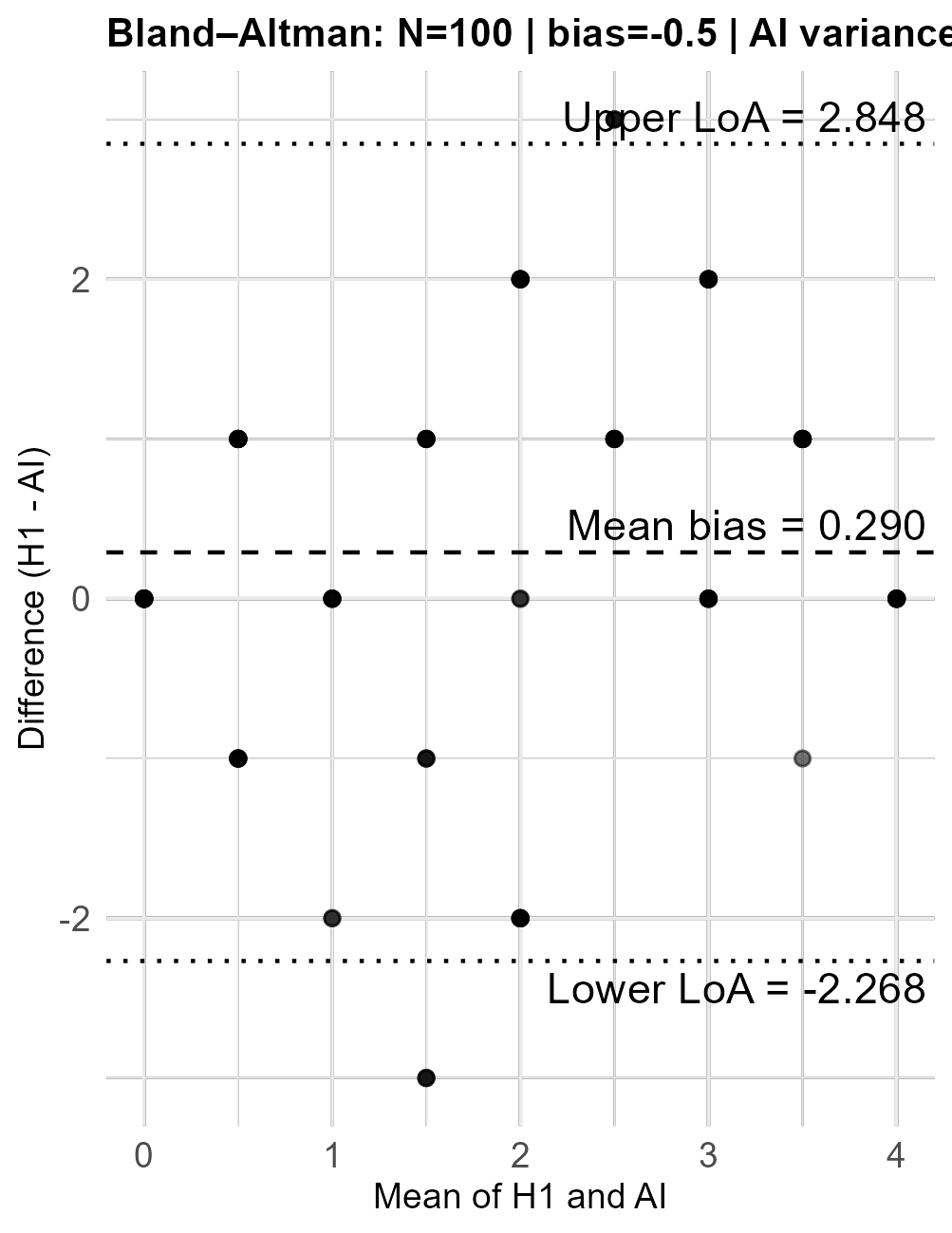

Supplement: Supplementary file 4 [file Data_Sheet_4.zip › BA_N100_bias-0.5_aihigh_compTRUE_imbFALSE_fairTRUE_rep1.tif]

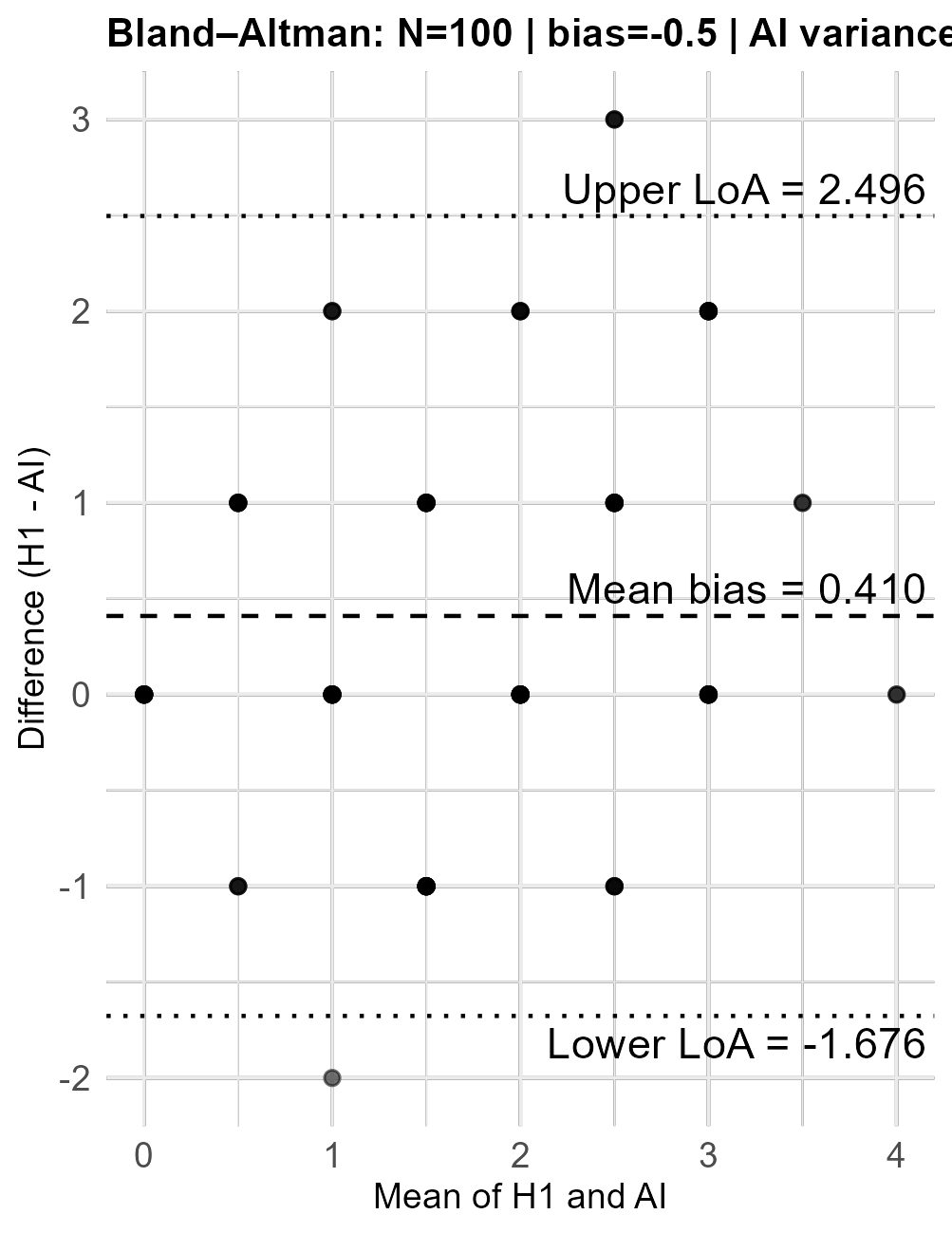

Supplement: Supplementary file 4 [file Data_Sheet_4.zip › BA_N100_bias-0.5_aihigh_compTRUE_imbTRUE_fairFALSE_rep1.tif]
